# Charge-Assisted Hydrogen-Bonding Enables Quantitative Multicomponent Self-Assembly in Strongly Polar Environments

**DOI:** 10.1021/acscentsci.6c00472

**Published:** 2026-05-15

**Authors:** Beatriz Torres-Calvo, Alberto de Juan, Isabel López-Martín, Miguel García-Iglesias, David González-Rodríguez

**Affiliations:** † Nanostructured Molecular Systems and Materials Group, Organic Chemistry Department, Universidad Autónoma de Madrid, 28049 Madrid, Spain; ‡ Institute for Advanced Research in Chemical Sciences (IAdChem), 16722 Universidad Autónoma de Madrid, 28049 Madrid, Spain; § QUIPRE Department, Nanomedicine-IDIVAL, 16761 Universidad de Cantabria, Santander, 39005, Spain

## Abstract

Multicomponent discrete assemblies, composed of a defined number of molecular building blocks, represent valuable supramolecular constructs with broad functional potential. Their formation relies on cooperativity, on one hand, and on directional noncovalent interactions, on the other. Many of these interactions have a marked electrostatic component, like H-bonding, and cannot survive in strongly competing polar solvents. A notable exception is the coordination bond, which offers enough strength and selectivity to enable assembly in such polar environments. In this work, we introduce a unique, versatile alternative based on amidinium–carboxylate salt bridges. Concretely, a [2+4] hexacomponent assembly, comprising 2 cofacial tetracarboxylate porphyrins linked through 4 diamidine spacers that exhibits remarkably high kinetic and thermodynamic stability is quantitatively formed in DMSO-rich environments, as a result of the charge-assisted hydrogen-bonding interactions employed and the strong chelate cooperativities attained.

## Graphical abstract

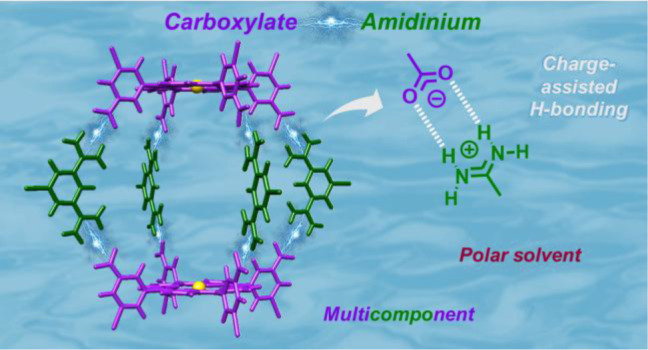

## Introduction

Assemblies comprising a discrete number of precisely organized molecules, thus defining a distinct structure, constitute challenging, highly valued synthetic supramolecular targets, with potential applications in catalysis,^1^ sensing,^2^ or drug delivery.^3^ Two are the key ingredients required to build such discrete assemblies with quantitative fidelity from a finite number of components. The first one is *cooperativity*. The reason why multitopic molecules prefer to be organized in discrete cyclic assemblies instead of ill-defined polymeric structures is because intramolecular interactions arise in the former that may enjoy strong chelate cooperativities.^4^ The second one is the *intermolecular interaction* itself. Here, the supramolecular chemist can choose among diverse noncovalent bonds, each of them having their own features.^5^ The binding interaction selected will also determine the *environment* in which these discrete structures will be created. Synthetic multicomponent assemblies are preferably formed in apolar organic solvents because these solvents do not interfere with the directional noncovalent bonds holding the structure, which typically have a pronounced electrostatic component, like H-bonding,^6,7^ for which strongly polar solvents fiercely compete. There is, however, a unique and prolific exception to this rule: the coordination bond, which can be sufficiently robust and selective to persist in polar environments like H_2_O, MeOH, DMF, or even DMSO.^8,9^ Biological systems, in contrast, maintain directional electrostatic interactions in polar media by “burying” them in hydrophobic pockets^10,11^ and by cooperatively combining them around localized functional groups.

An example of the latter are the “*salt bridges*” that can be found in arginine-aspartate pairs,^12,13^ RNA stem loops,^14^ DNA complexes,^15^ and in the active sites of manifold enzymes. Single-point ionic or H-bonding interactions cannot provide enough binding strength to maintain a supramolecular structure in polar environments, but their synergic combination, in the form of carboxylate/phosphate–amidinium/guanidinium “bridges” is known to provide high complementarity, directionality, and binding strength in diverse media (Figure 1).^16^ The association between carboxylic acid and amidine units can provide a double H-bonded *DA:AD* interface with association constants that are exceptionally large, due to the complementary electrostatic charges generated.^17–19^ As a result, this interaction has been employed in apolar aromatic and chlorinated solvents to study proton-coupled electron transfer processes,^19,20^ and to produce a wide diversity of supramolecular systems like helicates, catenanes or cylindrical capsules.^21–26^ Polar solvents, on the other hand, favor proton transfer to generate the carboxylate:amidinium *AA:DD* ion pair, in which the H-bonds are reinforced by the oppositely charged interface. Such “charge-assisted H-bonding” in highly polar media has been exploited to construct heterodimeric structures from calixarene,^27–29^ anthracene and related^28,29^ scaffolds, or to induce the formation of crystals,^30–33^ monolayers,^31^ and H-bonded organic frameworks.^32,33^ However, despite the strong potential of salt bridges to build discrete H-bonded multicomponent systems (*i.e*. composed of more than 2 components) in strongly polar media with complete fidelity,^34^ the realization of this goal needs to be proven.

**Figure 1:**
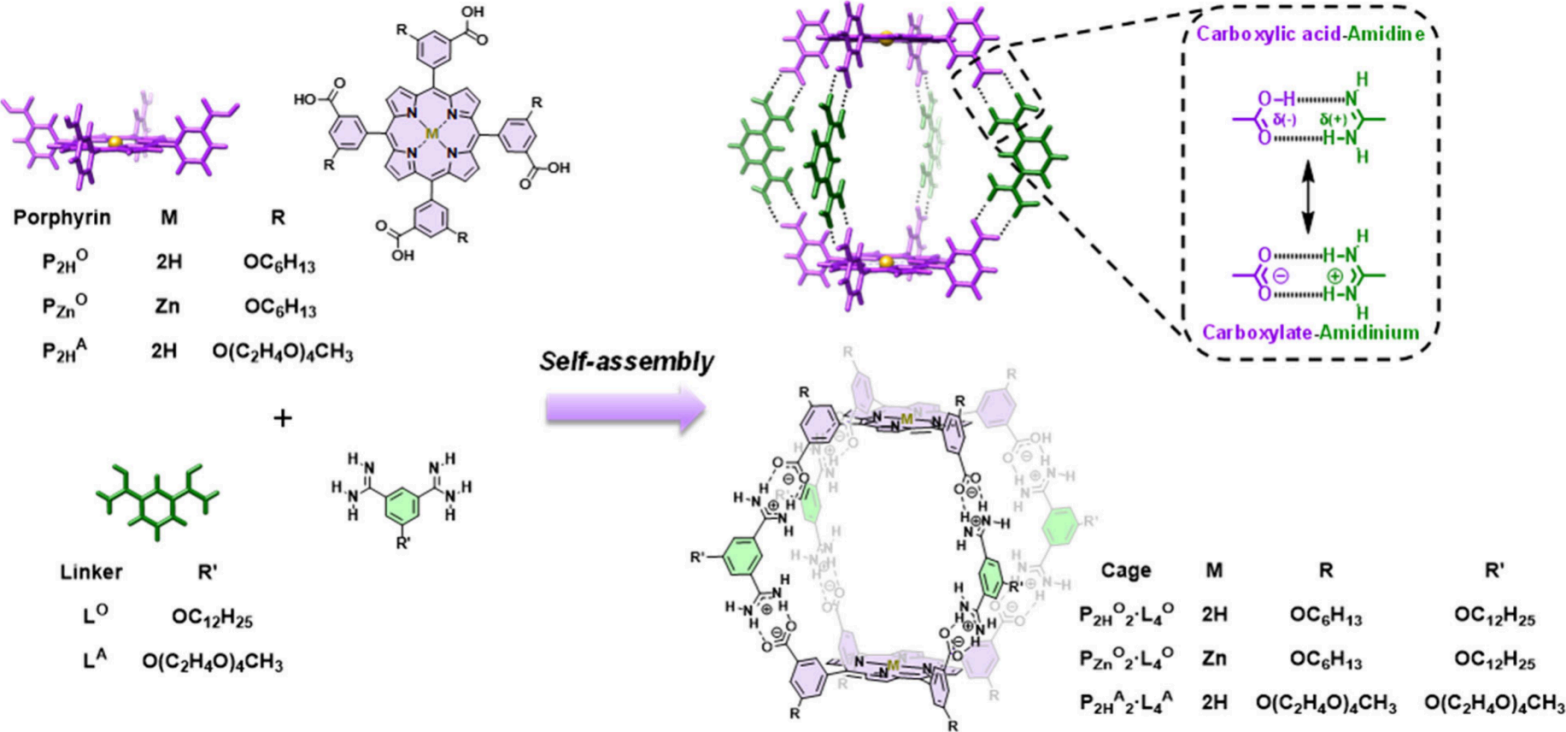
Chemical structures and models of the two families of **P** and **L** molecules involved in the self-assembly process to form the supramolecular **P**_**2**_ **·L**_**4**_ species. The inset shows two extreme situations with only partial or total proton transfer at the carboxylic acid:amidine H-bonded interface.

Here we show a unique example of a discrete multicomponent assembly that is quantitatively formed in strongly polar solvents by noncovalent forces that are different to the coordination bond. Concretely, a system comprising 2 cofacially disposed Zn^II^ porphyrins, an arrangement widely employed in the construction of three-dimensional assemblies for various applications,^35–39^ fused by 4 linkers is built through charge-assisted H-bonding interactions. This 6-component ensemble displays a strong chelate cooperativity and thus exceptionally high kinetic and thermodynamic stabilities, even in highly competing DMSO-rich environments.

## Results and Discussion

The synthesis and characterization of the two components in our supramolecular assemblies (Figure 1), tetracarboxylic acid porphyrins (**P**_**2H**_ or **P**_**Zn**_) and diamidine linkers (**L**), is detailed in the Supporting Information (S.I.) accompanying this paper. The rigid structure of both components, as well as the 120° *m*-connections of the carboxylic acid groups with respect to the **P** *meso*-positions and between the amidine groups in the **L** units, is meant to provide unstrained cyclic entities through the establishment of 8 linear salt-bridge interactions, leading to the [2+4] container depicted in Figure 1. Each of these **P** and **L** components is either substituted by apolar alkyl tails (**P** ^**O**^ **/ L** ^**O**^) or by oligo­(ethylene glycol) chains (**P** ^**A**^ **/ L** ^**A**^). The **P** and **L** compounds are relatively soluble in a wide diversity of solvents, but their 1:2 mixture exhibited quite remarkable insolubility in both apolar (toluene, CHCl_3_, CH_2_Cl_2_, CHCl_2_CHCl_2_, THF) and polar (acetone, DMF, DMSO, MeOH) environments. Some of the mixtures of these solvents, however, afforded adequate solubility, and thus intermolecular interactions were studied there. For instance, 9:1 to 1:9 mixtures of THF-D_8_ and DMSO-D_6_ supplied full solubility of the 1:2 **P** + **L** combinations below 10^–2^ M. Surpassing these solvent or concentration limits resulted in (partial) precipitation of both components together, while each of them individually remained soluble in the same conditions.

Self-assembly was first examined in a set of ^1^H NMR experiments where the proportion of each **P** and **L** component was varied from 1:0 to 0:1 (Figure 2a and S1). At exactly the stoichiometric 1:2 ratio, a single set of proton signals is detected that is different from the individual **P** and **L** components, which is compatible with a supramolecular structure exhibiting *D*_4h_-symmetry. The amidinium bound and unbound protons are respectively found between 13 and 14 ppm and 8–9 ppm, the latter exhibiting broader signals and chemical shifts that are more dependent on solvent composition. Interestingly, between the 1:0 and 1:2 ratios (excess of **P**; top spectra in Figure 2a), bound and unbound **P** units are detected in *slow* NMR exchange, which is a typical sign for the formation of highly cooperative, kinetically and thermodynamically stabilized supramolecular species. On the contrary, when analyzing the evolution of the ^1^H NMR spectra between 0:1 and 1:2 ratios (excess of **L**; bottom spectra in Figure 2a), associated and dissociated **L** units are seen in *fast* NMR exchange, and we are able to monitor a single averaged set of **L** proton signals that shift downfield as the complex is formed. Thus, under these conditions we are forming a supramolecular species from two different molecules where one of them exchanges slowly and the other one rapidly within the NMR time scale. Rising up temperature to 333 K or cooling down to 263 K (Figure S2) does not change qualitatively this scenario, which is on the other hand reasonable, since in order to detach the **P** unit from the complex, 4 salt-bridges need to be dissociated, whereas to release a **L** unit, only 2 of them need to be broken. It is important to highlight that the observation of a unique set of NMR signals at exactly a 1:2 **P**:**L** ratio that is in slow exchange with the constituents is a solid proof for the formation of a single (multi)­cyclic self-assembled structure with *D*_4h_ symmetry and a 1:2 **P**:**L** stoichiometry,^40^ and allows to verify that is formed quantitatively in solution, or at least within the NMR detection limits. This situation contrasts with previous studies that targeted the formation of related assemblies,^27^ where the interaction between amidinium and carboxylate counterparts could be confirmed in DMSO.

**Figure 2:**
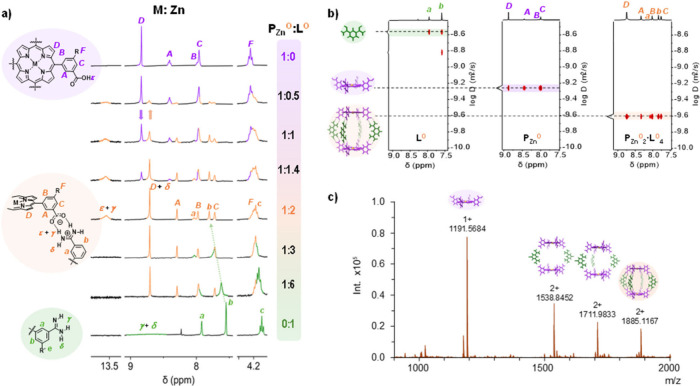
Selected regions of **a**) the ^1^H NMR spectra of mixtures of **P**_**Zn**_^**O**^ and **L** ^**O**^ in different proportions, and **b**) the ^1^H DOSY NMR spectra of **L** ^**O**^ (6.9 mM), **P**_**Zn**_^**O**^ (6.9 mM), and **P**_**Zn**_^**O**^_**2**_ **·L** ^**O**^_**4**_ (2.3 mM). All experiments were performed in a THF-D_8_/DMSO-D_6_ (75:25) mixture at 298 K. The signals marked in purple and green correspond to the free **P** and **L** subunits, respectively, while those in orange correspond to the self-assembled subunits (**P**_**2**_ **·L**_**4**_). **c**) HR-MS ESI-Q-TOF spectrum of **P** ^**O**^_**2**_ **·L** ^**O**^_**4**_ recorded at 273 K in a THF/DMSO 10:1 mixture.

In order to evaluate the dimensions of the assembly formed in solution by amidinium:carboxylate interactions, DOSY NMR experiments were next performed (see Figure S3). Figure 2b compares the diffusion coefficients obtained for the 0:1, 1:0 and 1:2 samples of **P** and **L** molecules. Two observations must be remarked from these experiments that support the formation of the proposed [2+4] complex. First, both **P** and **L** components diffuse together in the 1:2 mixtures as a single supramolecular entity. Second, they do it with a diffusion coefficient that is compatible with a structure having a considerably larger hydrodynamic radius than the individual components, which was calculated as 1.1 nm using the Stokes–Einstein equation for a spherical object. These dimensions compare reasonably well with the 1.4 nm radius obtained in computational models, taking into account that our spherical assembly is not solid, but has a void solvent-accessible lumen. Remarkably, the DOSY NMR spectrum of a 1:1 **P**+**L** sample revealed different diffusion coefficients for the bound and unbound **P** units in slow NMR exchange (Figure S5). On the other hand, ESI-MS experiments revealed the parent [**P**_**2**_ **·L**_**4**_]^2+^ ion peak, with a matching isotopic distribution with respect to the theoretical value, along with other ions attributed to the sequential loss of **L** units (Figure 2c and S6).

The **P**_**2**_ **·L**_**4**_ complex exhibited high stability in the mentioned 9:1 to 1:9 mixtures of THF-D_8_ and DMSO-D_6_ (Figure S7–8), and resisted temperature (up to 330 K; Figure S2) and concentration (down to 10^–5^ M, our ^1^H NMR limits; Figure S9) changes without significant disintegration (section S5). The absorption or emission of the **P** component did not display marked changes in the bound and unbound state, so dissociation could not be studied by these more sensitive techniques at lower concentrations (Figure S10). Instead, in order to dissociate the **P**_**2**_ **·L**_**4**_ complex and calculate the underlying association constant (*K*_T_; Figure 3a), we performed competition experiments with increasing amounts of benzoic acid (**B**). As shown in Figure 3b and S11–12, the addition of a few equivalents (<4 equivs) of this monotopic ligand, able to compete with the **P** component for the amidinium sites, does not result in notable changes in the ^1^H NMR spectra. However, the addition of larger amounts of **B** results in the release of the **P** unit due to the formation of the 2:1 **B**_**2**_ **·L** complex (Figure S11–12). Full destruction of the **P**_**2**_ **·L**_**4**_ assembly required >175 equivs of **B**, which underlines the highly cooperative nature of our multicomponent complex. From the equilibrium constant determined in this competition (*K*_C_) and the association constant of the **B**_**2**_ **·L** complex (*K*_a2_; Figure S13–14), calculated in separate experiments, we determined the formation constant of the **P**_**2**_ **·L**_**4**_ complex as *K*_T_ = *K*_a2_^4^/*K*_C_ = 1.6·10^30^ M^–5^ (see Figure 3a and S15 for further details). Along the formation of the **P**_**2**_ **·L**_**4**_ assembly (Figure 3c), 3 cycles are generated by “intramolecular” amidinium:carboxylate interactions, each of them associated with an effective molarity (*EM*) value. Hence, *K*_T_ can be related to the individual amidinium:carboxylate binding constant (*K*_a_), calculated with reference amidine and carboxylate compounds, as *K*_T_ = 32·*K*_a_^8^· $\overset{®}{EM}$ ,^3^ where 32 is the statistical factor (see Figure S16–17) and $\overset{®}{EM}$ is the mean value of the 3 cooperative cyclization process leading to **P**_**2**_ **·L**_**4**_, which was estimated as $\overset{®}{EM}$ = 0.157 M. This value is quite extraordinary for a supramolecular complex,^41,42^ especially considering that it is formed from 4 molecules of one kind and 2 molecules of another kind, and clearly explains, together with the relatively high association constant between carboxylate and amidinium motifs, why **P**_**2**_ **·L**_**4**_ displays such remarkable thermodynamic and kinetic stability.

**Figure 3:**
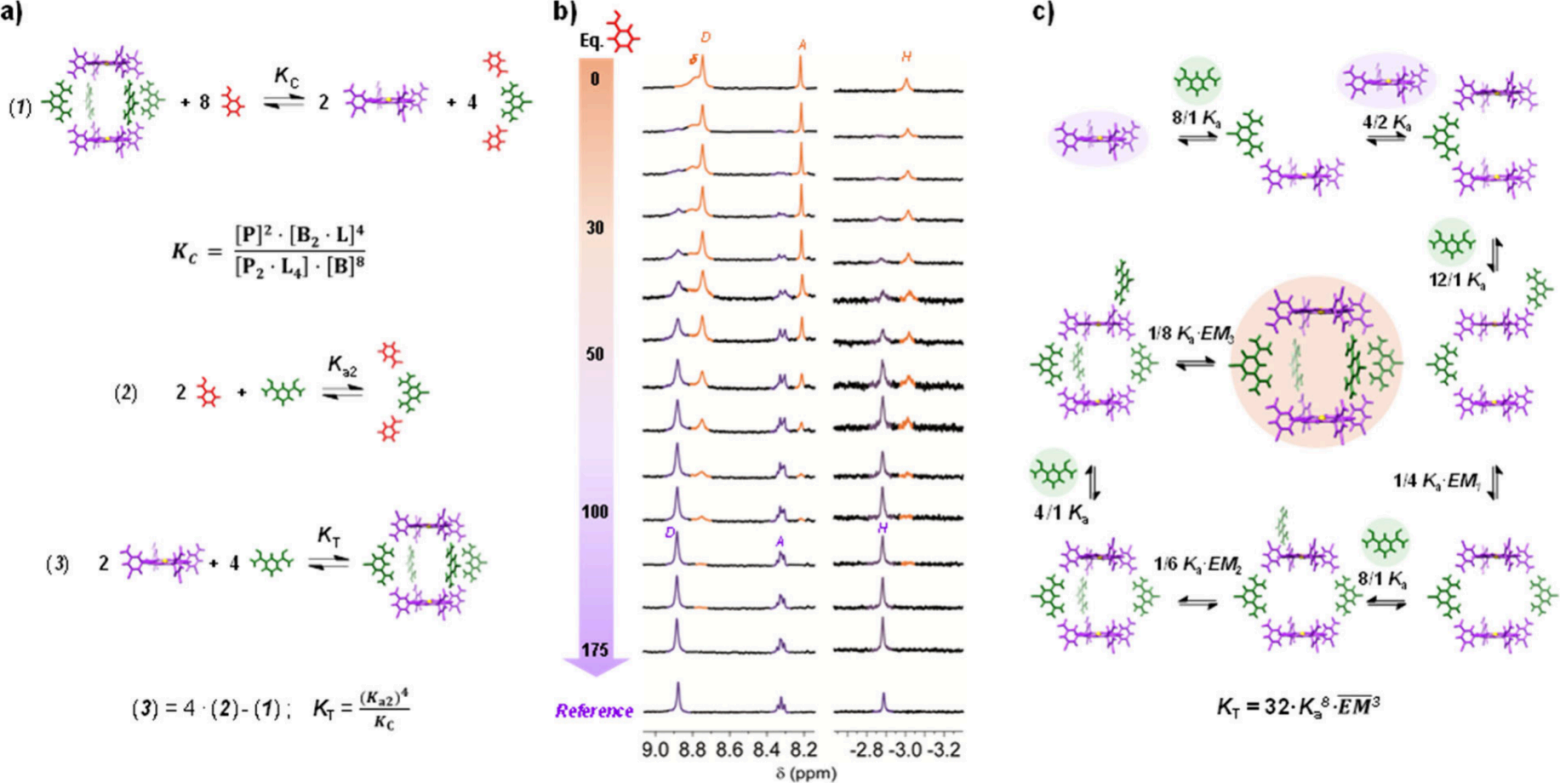
**a**) Schemes of the most relevant supramolecular equilibria ((1)-(3)) and equations to calculate *K*_T_. **b**) ^1^H NMR spectra obtained along the titration of **P**_**2H**_^**O**^_**2**_ **·L** ^**O**^_**4**_ with increasing amounts of **B** at a constant **P**_**2**_ **·L**_**4**_ concentration of 1.0 × 10^–4^ M in a THF-D_8_/DMSO-D_6_ (25:75) mixture at 298 K. **c**) Scheme of a plausible evolution along the formation of **P**_**2**_ **·L**_**4**_, including the binding constants, statistical factors and *EM* values for each individual step, as well as the global equation. Note that the proton assignment in Figure 3b is identical to that shown in Figure S12.

Attempts were also made to demonstrate the formation of these **P**_**2**_ **·L**_**4**_ assemblies in polar protic (methanol) and aqueous environments (Figure S18–20), which made us redesign our system with the **P** ^**A**^ and **L** ^**A**^ building blocks (Figure 1), equipped with oligo­(ethylene glycol) chains. The 1:2 **P**:**L** mixtures were insoluble in these protic solvents, but (partial) solubility could be achieved in mixtures with THF (see Figures S18–20). Although a similar supramolecular scenario as that observed in THF:DMSO mixtures (Figure S21) could be preliminary hinted, the strong hydrophobic effects induced by water produced broad NMR features (see Figure S20) that hampered an analogous detailed analysis. On the other hand, upon addition of small amounts of water to the preformed **P**_**2H**_^**A**^_**2**_ **·L** ^**A**^_**4**_ assembly in DMSO-rich samples resulted in dissociation of the components in slow NMR exchange (Figure S22).

Finally, a set of experiments was devised to demonstrate the ability of our **P**_**2**_ **·L**_**4**_ assemblies to perform as molecular containers and encapsulate molecules in their internal cavity. Our selected set of guests comprised dinitrogen ligands that can bind to the cofacially arranged Zn^II^ centers. Despite the strongly coordinating ability of our solvent systems, which are able to saturate Zn^II^ metal centers, dinitrogen molecules with a N···N distance matching the Zn^II^···Zn^II^ separation exhibited relatively strong association to the **P**_**2**_ **·L**_**4**_ complex. That is the case of the di­(pyridin-4-yl)­buta-1,3-diyne guest, for which a modest equilibrium constant of *K*_a_ = 2.2·10^2^ M^–1^ was calculated for the interaction with the **P**_**2**_ **·L**_**4**_ complex in THF-D_8_/DMSO-D_6_ 75:25 (Figure S23). It is important to mention that, under the same conditions, the monotopic **P**_**Zn**_ and di­(pyridin-4-yl)­buta-1,3-diyne, on one hand, or the shorter nonmatching 4,4′-bipyridine guest and **P**_**2**_ **·L**_**4**_, on the other, did not show any evidence of association. This highlights the importance of the cooperative binding to both Zn^II^ metal centers to reach strong association in this highly competing environment.

## Conclusions

In short, we described herein the formation of a unique multicomponent complex that is assembled in strongly polar environments by means of noncovalent interactions that are different to the coordination bond. The synergic interplay between ionic and H-bonding interactions in amidinium:carboxylate “salt bridges”, as well as the remarkable chelate cooperativities attained, enabled the formation of a [2+4] cofacially arranged bisporphyrin container. Our work demonstrates that charge-assisted hydrogen bonding can be harnessed to achieve robust and quantitative multicomponent self-assembly under experimental conditions where normal hydrogen-bonds should not be operative at all. We are convinced that the rational use of these charge-assisted H-bonds, maybe in cooperation with other compatible noncovalent forces, will afford a higher degree of versatility and functionality to realize the construction of complex supramolecular systems in polar environments.

## Supplementary Material



## Notes

Experimental details, procedures and compound characterization data: ^1^H and ^13^C NMR, DOSY NMR, as well as by HR-MS, UV–vis and emission spectroscopies, as well as additional supramolecular information. (PDF)
