# Supplementary material for: Charge-Assisted Hydrogen-Bonding Enables Quantitative Multicomponent Self-Assembly in Strongly Polar Environments
Source: ACS Cent Sci. 2026 May 15;12(6):841–6. doi: 10.1021/acscentsci.6c00472 (PMC13306597; doi:10.1021/acscentsci.6c00472)
Supplement: Supplementary file 1 [file oc6c00472_si_001.pdf]

SUPPORTING INFORMATION

Charge-assisted Hydrogen-bonding enables Quantitative Multicomponent Self-assembly in Strongly Polar Environments

Beatriz Torres-Calvo,[a] Alberto de Juan,[a,b] Isabel López-Martín,[a] Miguel García-Iglesias,[c] and David González-Rodríguez*[a,b]

[a] Nanostructured Molecular Systems and Materials group, Organic Chemistry Department, Universidad Autónoma de Madrid, 28049 Madrid, Spain

[b] Institute for Advanced Research in Chemical Sciences (IAdChem), Universidad Autónoma de Madrid, 28049 Madrid, Spain

[c] QUIPRE Department, Nanomedicine-IDIVAL, Universidad de Cantabria, Avd. de Los Castros, 46, 39005 Santander, Spain

SUPPORTING INFORMATION

TABLE OF CONTENTS

General Methods S3

Synthetic Procedures S4

Synthesis of **P^O** and **P^A** S5

Synthetic route to the O compound set. S6

Synthetic route to the A compound set. S16

Synthesis of **L^O** and **L^A** S26

Synthetic route to the O compound set. S27

Synthetic route to the A compound set. S31

S1. ^{1}H NMR Titration Experiments S35

S2. Temperature-dependent ^{1}H NMR experiments at different **P_{Zn}^O:L^O** ratios S37

S3. ^{1}H DOSY NMR experiments at different **P_{Zn}^O:L^O** ratios S38

S4. High-resolution mass spectrometry analysis of **$P_{2H}^O{}_2 \cdot L^O{}_4$** S41

S5. ^{1}H NMR experiments at different solvent compositions and concentrations S42

S6. Absorption and emission experiments S44

S7. Calculation of thermodynamic parameters: K_{a2}, K_C, K_T and *EM* S45

S8. Self-assembly in protic solvents and aqueous environments S51

S9. Host-Guest chemistry S56

Supplementary References S58

General Methods

High Resolution-Mass Spectrometry (**HRMS**) MALDI-TOF spectra were obtained from a BRUKER ULTRAFLEX III instrument equipped with a nitrogen laser operating at 337 nm, and APCI and ESI spectra were obtained from a BRUKER MAXIS II instrument.

NMR spectra were recorded with a *BRUKER AV-II* 300 MHz, *BRUKER AV* 500 MHz or a *BRUKER DRX* 500 MHz instruments. The temperature was actively controlled at 298 K. Chemical shifts are reported in ppm relative to the residual signals of the deuterated solvents as internal standards [$CDCl_3$ calibrated at 7.26 (^1H) and 77.2 ppm (^{13}C), DMSO-D_6 calibrated at 2.50 (^1H) and 39.5 ppm (^{13}C), D_2O calibrated at 4.79 (^1H)]. For mixtures of solvents, chemical shifts were referenced to tetramethylsilane (TMS) set at 0.00 ppm (^1H). For the ^1H DOSY NMR experiments, a series of diffusion ordered spectra were collected from the samples using the LEDbp pulse sequence. The pulse fields were incremented in 16 steps from 5 % to 95 % of the maximum gradient strength in a linear ramp.

Manual column chromatography purifications were carried out on silica gel *Merck-60* (230-400 mesh, 60 Å), and TLC on aluminum sheets precoated with silica gel 60 F254 (Merck). **Automatic column chromatography** purifications were carried out on CombiFlash® Rf 150-Teledyne Isco.

UV-Visible experiments were conducted using a *JASCO V-660* apparatus. **Emission spectra** were recorded in a *JASCO FP-8600* equipment using excitation and emission bandwidths of 5 nm in both cases, and a 0.1 s response. In both instruments the temperature was controlled using a *JASCO* Peltier thermostatted cell holder at 298 K, adjustable temperature slope, and accuracy of ± 0.1 K.

Chemical structures were generated with *ChemDraw* 23.1.1, [1] and **molecular models** were created using either *Hyperchem* (version 8.0.3 for Windows) [2] or *Chimera* (version 1.16).[3] Molecular structures were first optimized through semiempirical PM3 methods and then assembled together at the correct distances and angles, with specific dihedral angles constrained.

Titration experiments

To obtain the different **P**:**L** ratios (Section S1-S6, and S8), stock solutions of **P** and **L** at the same concentration were mixed according to the desired proportion. For the determination of thermodynamic parameters, experiments were carried out twice at constant host concentration. This was achieved by progressively adding aliquots of a solution containing the same host concentration and a large excess of guest molecules (7-200 equivalents) to a host solution, until the monitored signals stabilized (see Sections S7 and S9). The experimental data were fitted to 1:1 or 1:2 equilibrium models using the free software available at www.supramolecular.org (see S7 and S9 for further details).

Starting materials

Chemicals were purchased from commercial suppliers and used without further purification, except pyrrole which was distilled prior to be used.

Synthetic Procedures

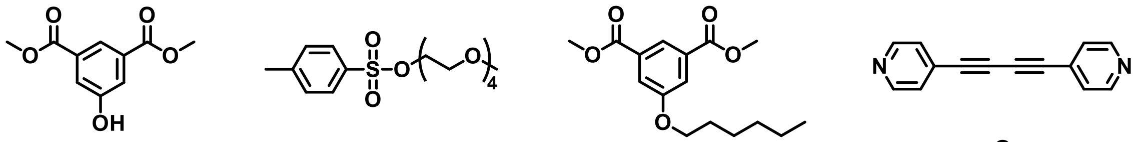

1 **2** **3**^{O} **G**

Scheme S1. Compounds previously described.

The synthetic procedures and characterization data of compounds **1**,[4] **2**,[5] **3**^{O},[6] and **G**[7] have been already reported.

Synthesis of P^O and P^A

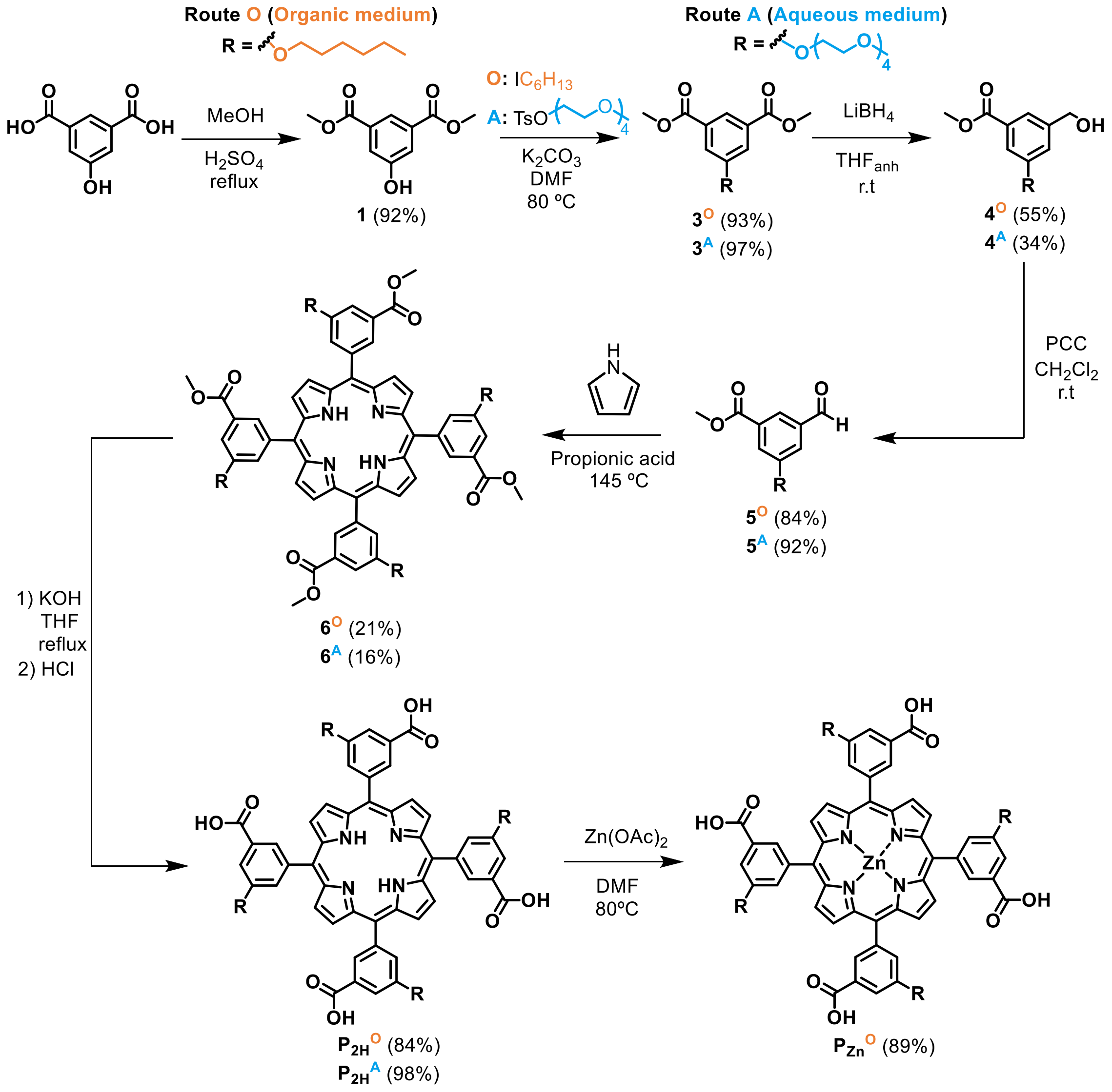

Scheme S2. Synthetic routes **O** (organic) and **A** (aqueous) to prepare the corresponding tetracarboxylic acid porphyrin.

Synthetic route to the O compound set.

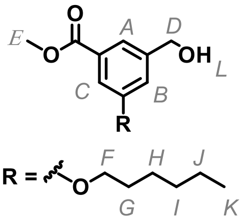

Compound 4[O]. A solution of compound **3**[O] (11.71 g, 39.78 mmol, 1 eq.) in anhydrous THF (88 mL) was prepared under an argon atmosphere. A 2 M solution of $LiBH_4$ in THF (11.9 mL, 23.87 mmol, 0.6 eq.) was then injected dropwise. After stirring for 40 hours, the reaction was quenched at 0 °C by adding EtOAc (50 mL), H_2O (50 mL), and 2 M HCl (20 mL). The mixture was subsequently extracted with EtOAc (3 x 100 mL). The combined organic phases were washed with 2 M HCl (1 x 50 mL) and H_2O (1 x 50 mL). The organic phase was dried over $MgSO_4$, filtered, and concentrated under reduced pressure. The crude product was purified by silica gel column chromatography (cyclohexane/EtOAc 85:15), affording a yellow oil. Yield: 55% (5.86 g, 21.99 mmol).

^{1}H NMR (300 MHz, $CDCl_3$) δ (ppm) = 7.60 (m, 1H, H^A), 7.46 (m, 1H, H^C), 7.12 (m, 1H, H^B), 4.70 (s, 2H, H^D), 4.00 (t, J = 6.5 Hz, 2H, H^F), 3.91 (s, 3H, H^E), 1.79 (m, 2H, H^G), 1.47 (m, 2H, H^H), 1.40-1.30 (m, 4H, H^{I-J}), 0.90 (t, J = 6.8 Hz, 3H, H^K).

^{13}C NMR (75 MHz, $CDCl_3$) δ (ppm) = 167.1, 159.5, 142.8, 131.6, 120.1, 118.2, 114.1, 68.5, 64.8, 52.3, 31.7, 29.3, 25.8, 22.7, 14.1.

HRMS (**APCI+**): Calculated for $C_{15}H_{23}O_4$ $[M+H]^+$ = 267.1591, found at m/z = 267.1594.

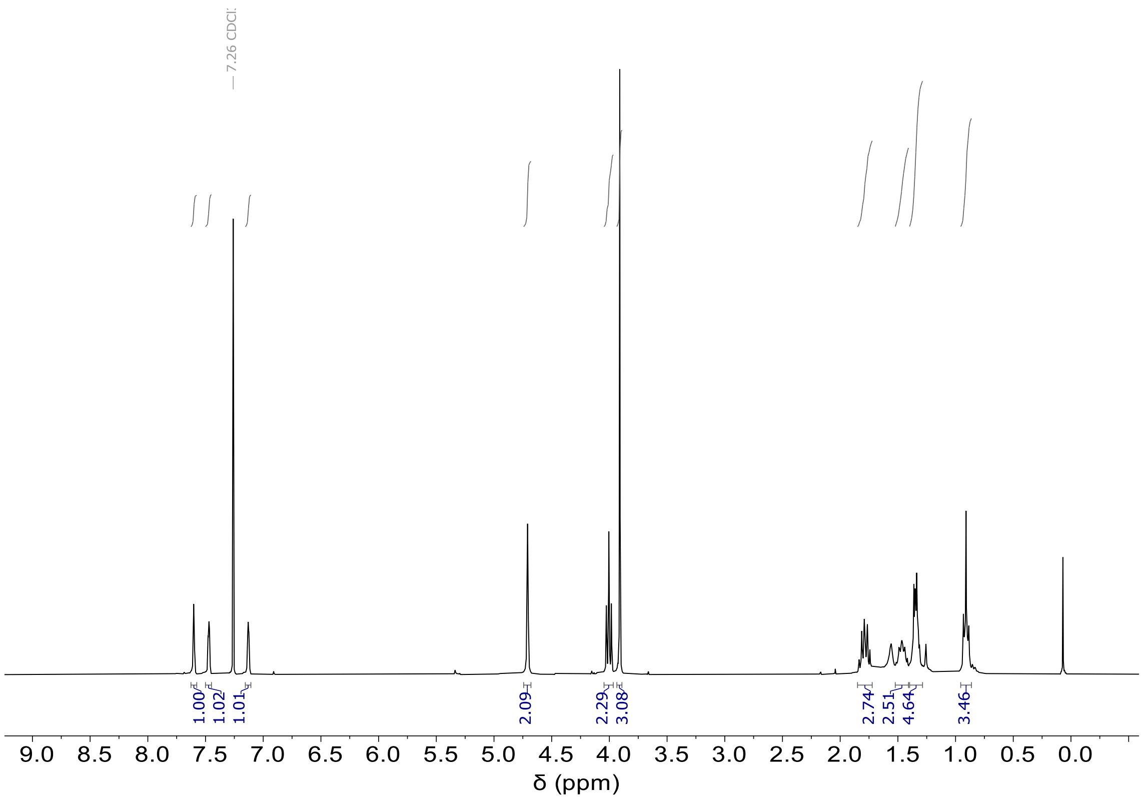

^{1}H NMR spectra of compound **4°** in $CDCl_3$ (298K, 300 MHz).

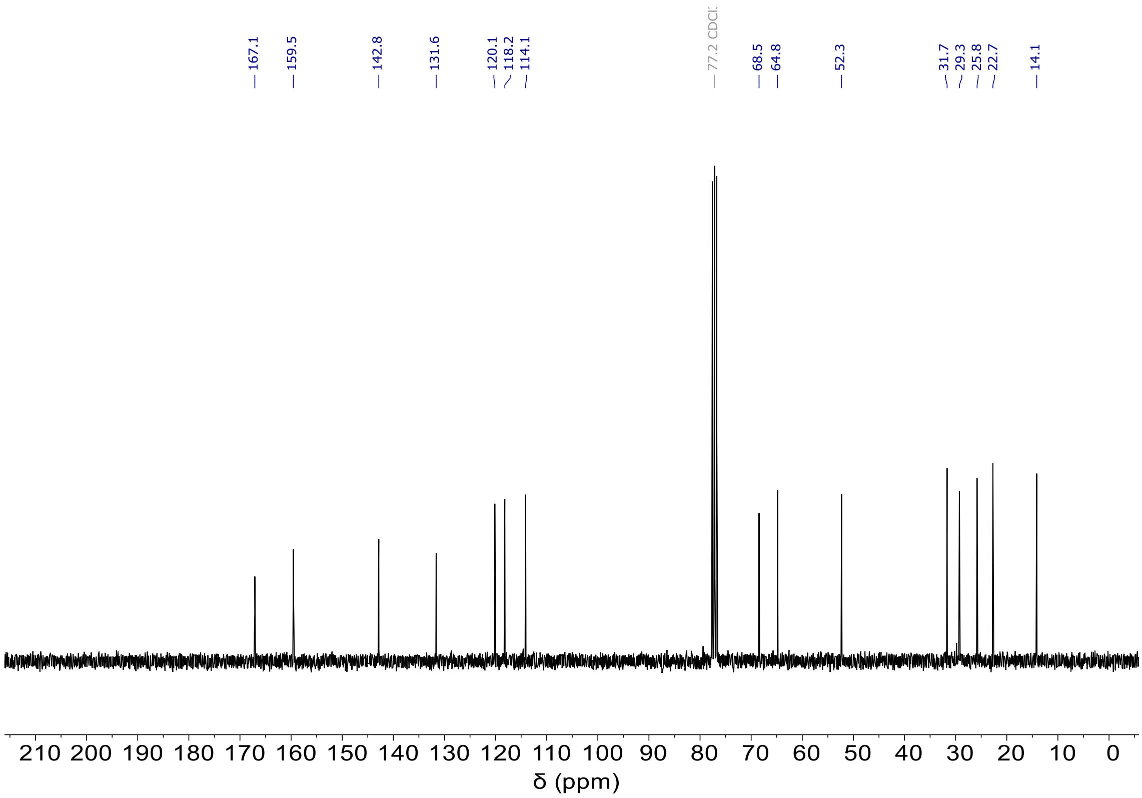

^{13}C NMR spectra of compound **4°** in $CDCl_3$ (298K, 75MHz).

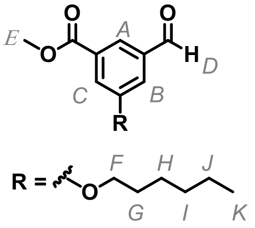

Compound 5[O]. To a solution of **4**[O] (5.86 g, 21.99 mmol, 1 eq.) in CH_2Cl_2 (220 mL), pyridinium chlorochromate (7.11 g, 32.99 mmol, 1.5 eq.) and a spoon of Celite were added. A colour change from pinkish-brown to dark brown was observed. The reaction mixture was stirred at room temperature for one hour. The resulting suspension was filtered through a silica plug and washed with $CHCl_3$. The filtrate was purified by silica gel column chromatography (cyclohexane/EtOAc 80:20), affording a yellowish oil. Yield: 85% (4.92 g, 18.61 mmol).

^{1}H NMR (300 MHz, $CDCl_3$) δ (ppm) = 10.01 (s, 1H, H^{D}), 8.09 (dd, J = 1.4, 1.2 Hz, 1H, H^{A}), 7.81 (dd, J = 2.3, 1.4 Hz, 1H, H^{C}), 7.56 (dd, J = 2.3, 1.2 Hz, 1H, H^{B}), 4.05 (t, J = 6.5 Hz, 2H, H^{F}), 3.96 (s, 3H, H^{E}), 1.82 (m, 2H, H^{G}), 1.48 (m, 2H, H^{H}), 1.40-1.30 (m, 4H, H^{I-J}), 0.91 (t, J = 6.6, 3H, H^{K}).

^{13}C NMR (75 MHz, $CDCl_3$) δ (ppm) = 191.5, 166.1, 160.0, 138.0, 132.5, 124.3, 122.1, 117.5, 68.9, 52.7, 31.7, 29.2, 25.8, 22.7, 14.2.

HRMS (**ESI+**): Calculated for $C_{15}H_{21}O_4$ $[M+H]^+$ = 265.1434, found at m/z = 265.1432.

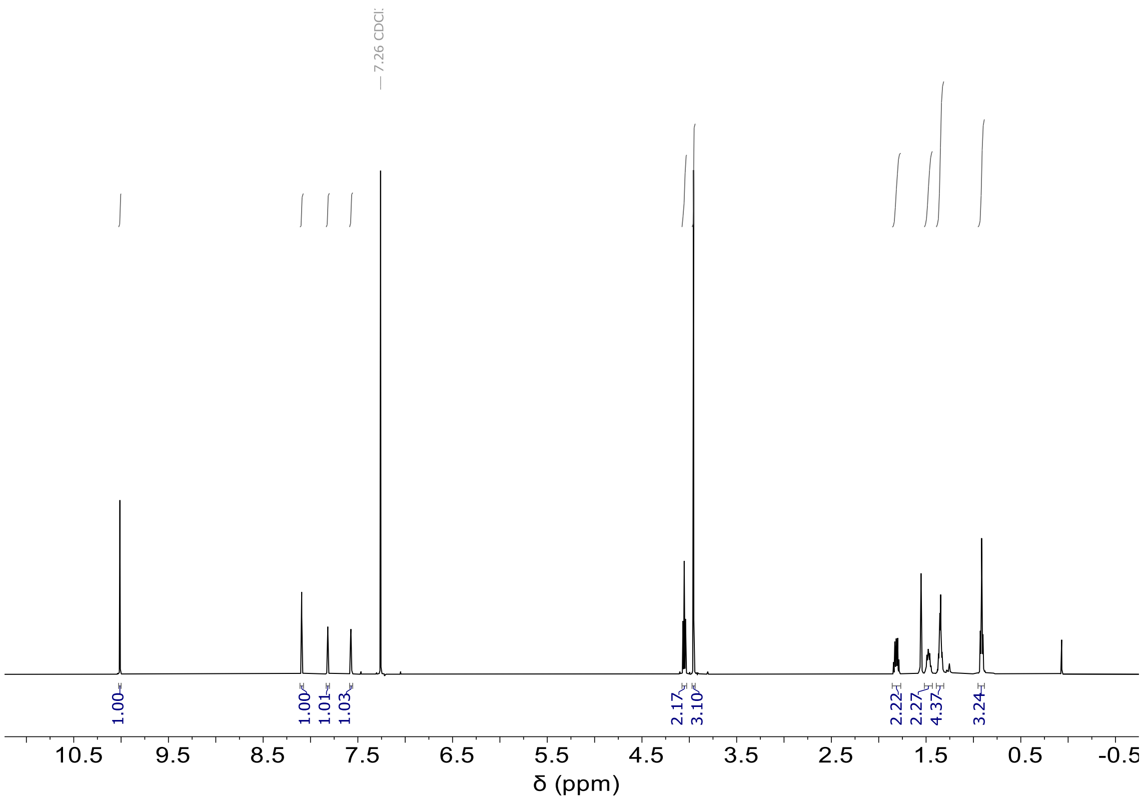

^{1}H NMR spectra of compound **5**^{O} in $CDCl_3$ (298K, 300 MHz).

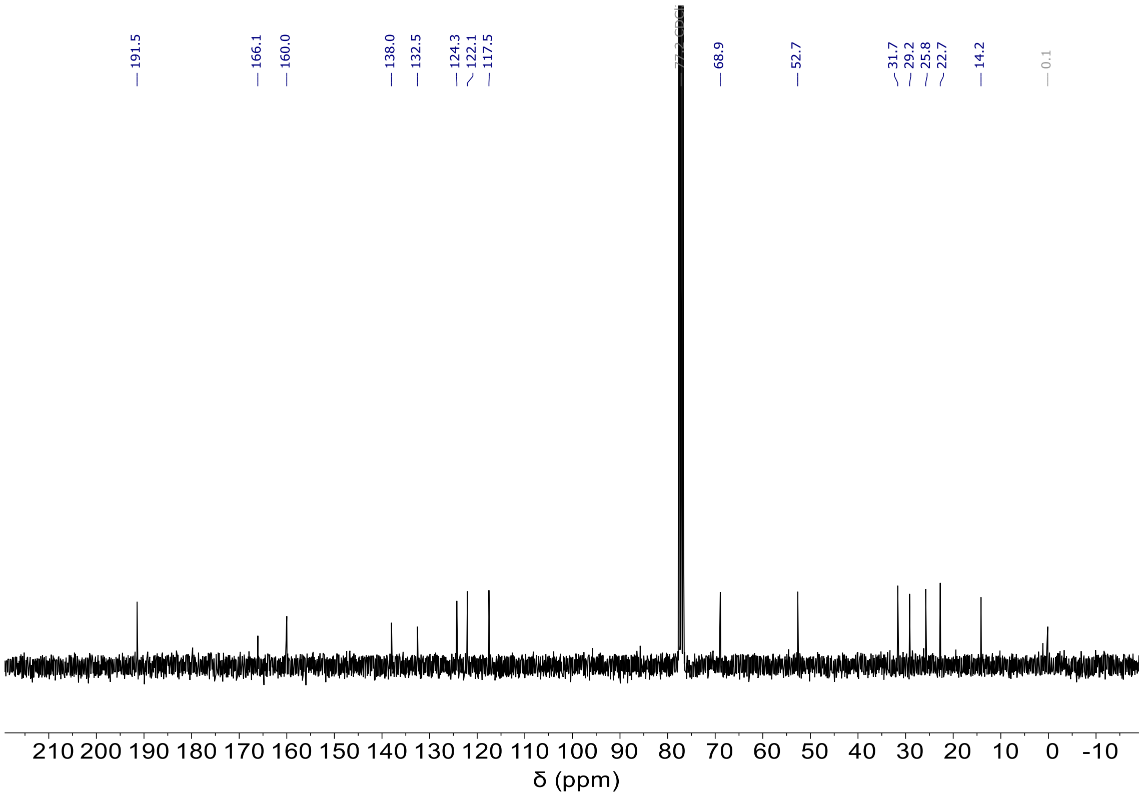

^{13}C NMR spectra of compound **5**^{O} in $CDCl_3$ (298K, 75 MHz).

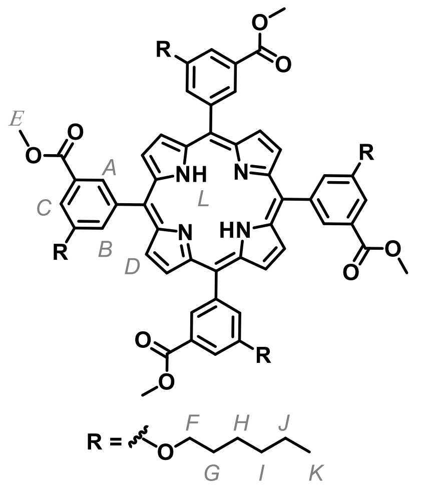

Compound 6[O]. Aldehyde **5**[O] (2.43 g, 9.21 mmol, 1 eq.) was dissolved in propionic acid (31 mL) under an inert atmosphere. Upon heating at 145 ºC, pyrrole (0.64 mL, 9.21 mmol, 1 eq.) was added. The reaction mixture was stirred at this temperature for three hours, protected from light. Then, the heat source and the inert atmosphere were removed, and stirring was continued overnight at room temperature. The reaction mixture was washed in EtOAc with a saturated $NaHCO_3$ solution (3 × 40 mL). Na_2SO_4 was used as a drying agent, then the mixture was filtered, and the solvent was removed under reduced pressure. The crude was purified twice by silica gel column chromatography (cyclohexane/EtOAc 20:1 to 10:1, followed by cyclohexane/$CHCl_3$ 1:1 to 1:2), affording a purple solid. Yield: 21% (0.60 g, 0.48 mmol).

^{1}H NMR (300 MHz, $CDCl_3$) δ (ppm) = 8.85 (s, 8H, H^D), 8.47 (m, 4H, H^A), 8.07-7.92 (m, 8H, $H^{B\text{-}C}$), 4.22 (t, J = 6.5 Hz, 8H, H^F), 3.97 (s, 12H, H^E), 1.90 (m, 8H, H^G), 1.60-1.19 (m, 24H, $H^{H\text{-}J}$), 0.89 (t, J = 7.0, 12 H, H^K), -2.84 (s, 2H, H^L).

^{13}C NMR (125 MHz, $CDCl_3$) δ (ppm) = 167.4, 157.8, 143.5, 131.5 (pyrr), 129.9, 128.4, 128.0, 126.0, 119.2, 114.7, 68.9, 52.6, 31.8, 29.4, 25.9, 22.8, 14.2.

HRMS (**ESI+**): Calculated for $C_{76}H_{87}N_4O_{12}$ $[M+H]^+$= 1247.6315, found at *m/z* = 1247.6298.

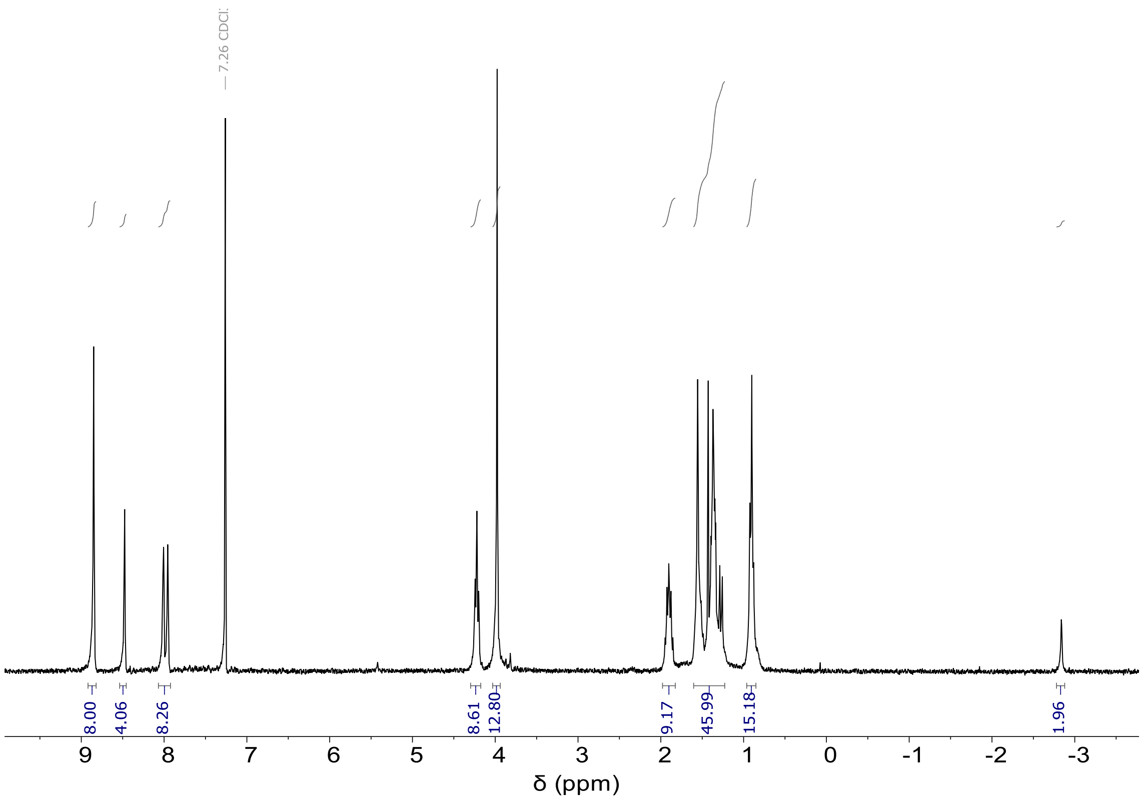

^{1}H NMR spectra of compound **6**[O] in $CDCl_3$ (298K, 300 MHz).

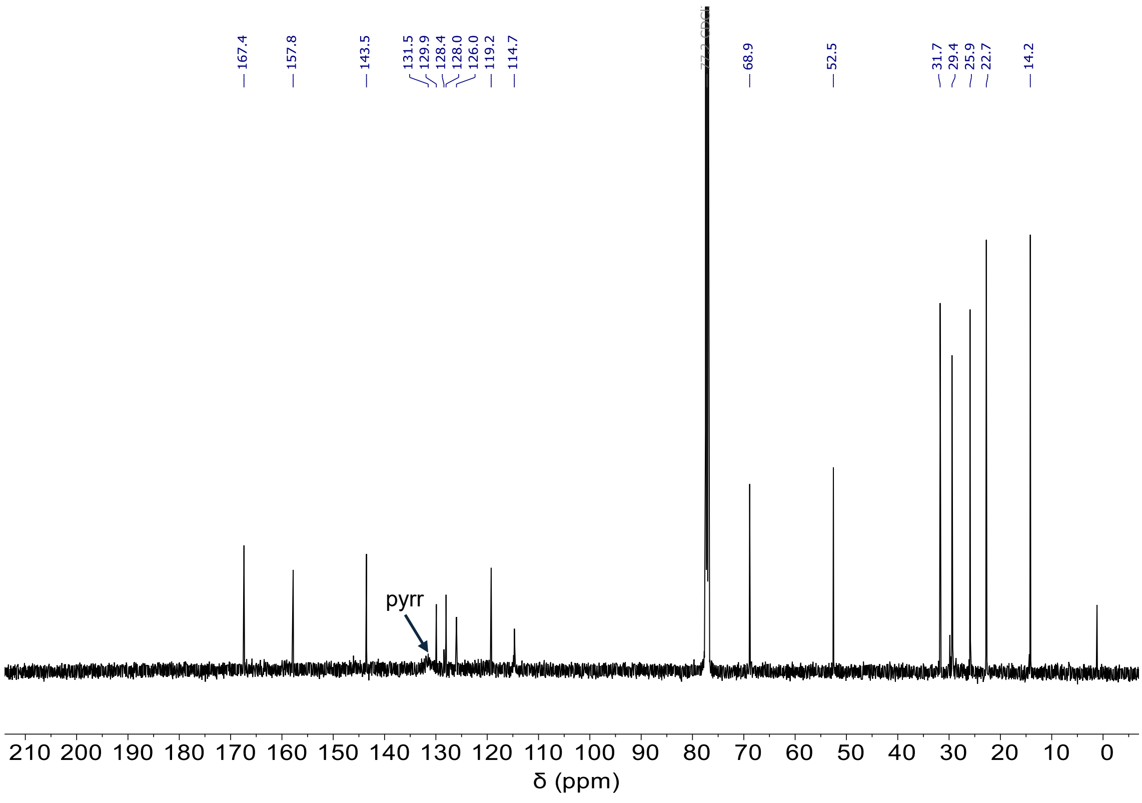

^{13}C NMR spectra of compound **6**[O] in $CDCl_3$ (298K, 125 MHz).

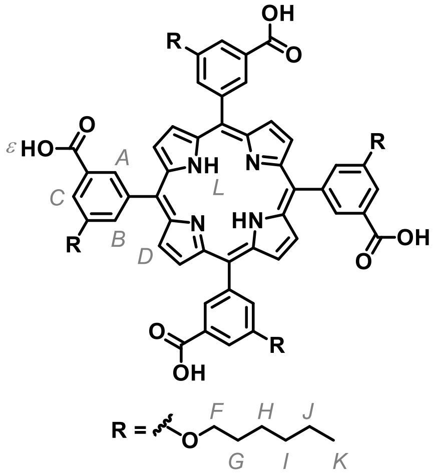

P$_{2H}$^{O}. To a solution of tetraester porphyrin **6**^{O} (0.60 g, 0.48 mmol, 1 eq.) in THF (86 mL), a solution of KOH (2.70 g, 48.09 mmol, 100 eq.) in H_2O (1.8 mL) was added. The mixture was heated to reflux overnight under magnetic stirring, yielding a green solution and a purple precipitate the following day. The solvent was removed under reduced pressure. The resulting solid was treated with 2 M HCl until the pH was adjusted to approximately 4, and extracted with CH_2Cl_2 until a colorless aqueous phase was obtained. Na_2SO_4 was used as a drying agent, the solution was filtered, and the solvent was removed under reduced pressure, affording a pure purple solid. Yield: 85% (0.48 g, 0.41 mmol).

^{1}H NMR (500 MHz, DMSO-D_6) δ (ppm) = 13.31 (broad s, 4H, H^{ε}), 8.88 (s, 8H, H^D), 8.28 (m, 4H, H^A), 8.03 (m, 4H, H^B), 7.89 (s, 4H, H^C), 4.22 (t, J = 6.6 Hz, 8H, H^F), 1.79 (m, 8H, H^G), 1.45 (m, 8H, H^H), 1.35-1.23 (m, 16H, $H^{I\text{-}J}$), 0.84 (t, J = 6.9 Hz, 12H, H^K), -2.96 (s, 2H, H^L).

^{13}C NMR (75 MHz, DMSO-D_6) δ (ppm) = 167.3, 157.3, 142.6, 131.6 (pyrr), 130.8, 127.4, 127.3, 124.8, 119.0, 114.7, 68.1, 31.0, 28.6, 25.1, 22.0, 13.8.

HRMS (**MALDI**, matrix: DCTB): Calculated for $C_{72}H_{78}N_4O_{12}$ $[M]^+$ = 1190.5611, found at m/z = 1190.5585.

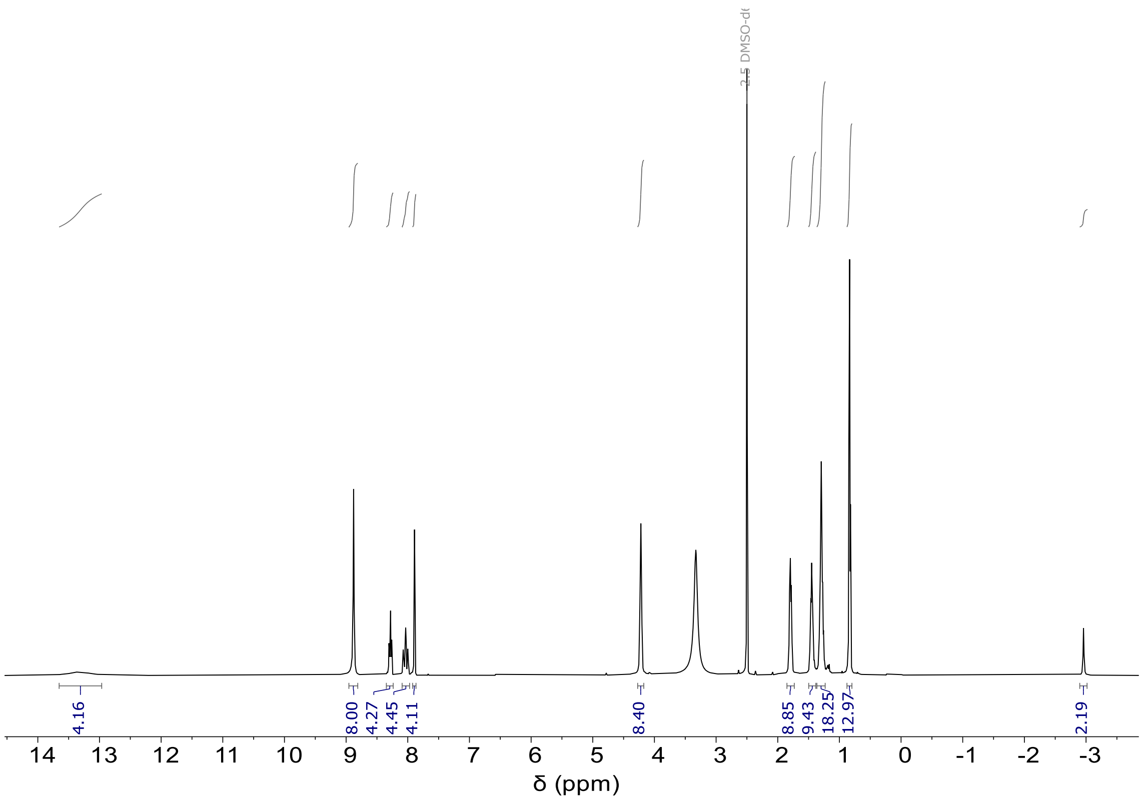

^{1}H NMR spectra of compound $\mathbf{P_{2H}}^{O}$ in DMSO-D$_6$ (298K, 500 MHz).

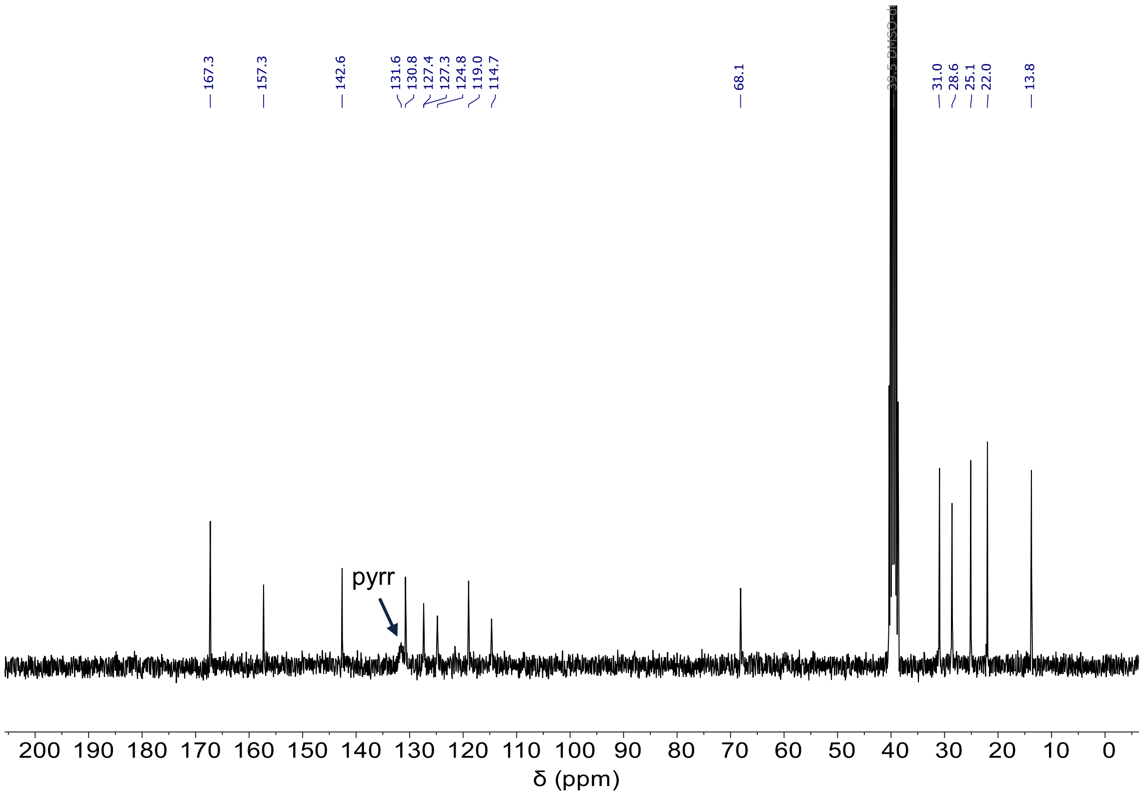

^{13}C NMR spectra of compound $\mathbf{P_{2H}}^{O}$ in DMSO-D$_6$ (298K, 75 MHz).

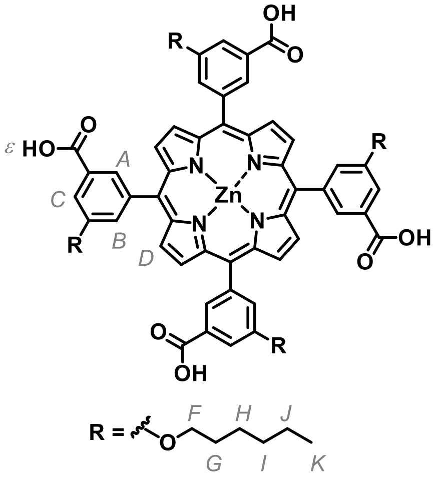

P$_{Zn}$^{O}. To a solution of compound **P$_{2H}$^{O}** (0.17 g, 0.14 mmol, 1 eq.) in DMF (12 mL), zinc acetate (0.13 g, 0.70 mmol, 5 eq.) was added. The mixture was stirred under magnetic agitation and heated at 80 ºC overnight. The solvent was removed under reduced pressure helping with an air stream. The crude was redissolved in EtOAc and washed with slightly acidic H_2O (3× 10 mL). The organic phase was dried over Na_2SO_4, filtered, and the solvent was evaporated under reduced pressure. Finally, the solid was triturated with acetonitrile, affording a purple solid. Yield: 89% (0.16 g, 0.12 mmol).

^{1}H NMR (500 MHz, DMSO-D$_6$) δ (ppm) = 13.20 (broad s, 4H, H^{ε}), 8.81 (s, 8H, H^D), 8.27 (m, 4H, H^A), 7.95 (m, 4H, H^B), 7.88 (s, 4H, H^C), 4.25 (m, 8H, H^F), 1.83 (m, 8H, H^G), 1.56-1.08 (m, 24H, $H^{H\text{-}J}$), 0.86 (t, J = 6.9 Hz, 12H, H^K).

^{13}C NMR (125 MHz, DMSO-D$_6$) δ (ppm) = 167.4, 157.0, 149.2, 144.1, 131.8, 130.3, 127.3, 124.8, 119.3, 114.0, 68.1, 31.0, 28.7, 25.2, 22.1, 13.9.

HRMS (**MALDI**, matrix: DCTB): Calculated for $C_{72}H_{76}N_4O_{12}Zn$ $[M]^+$ = 1252.4746, found at m/z = 1252.4709.

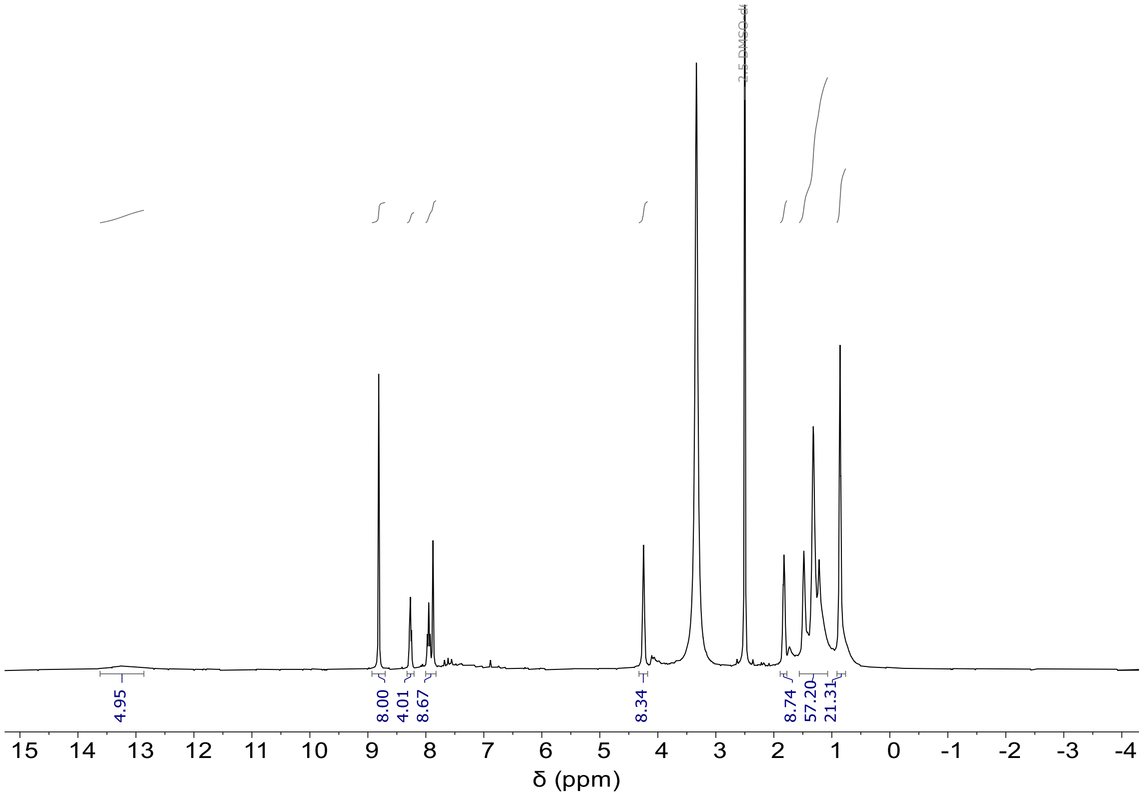

^{1}H NMR spectra of compound **P$_{Zn}$^{O}** in DMSO-D$_6$ (298K, 500 MHz).

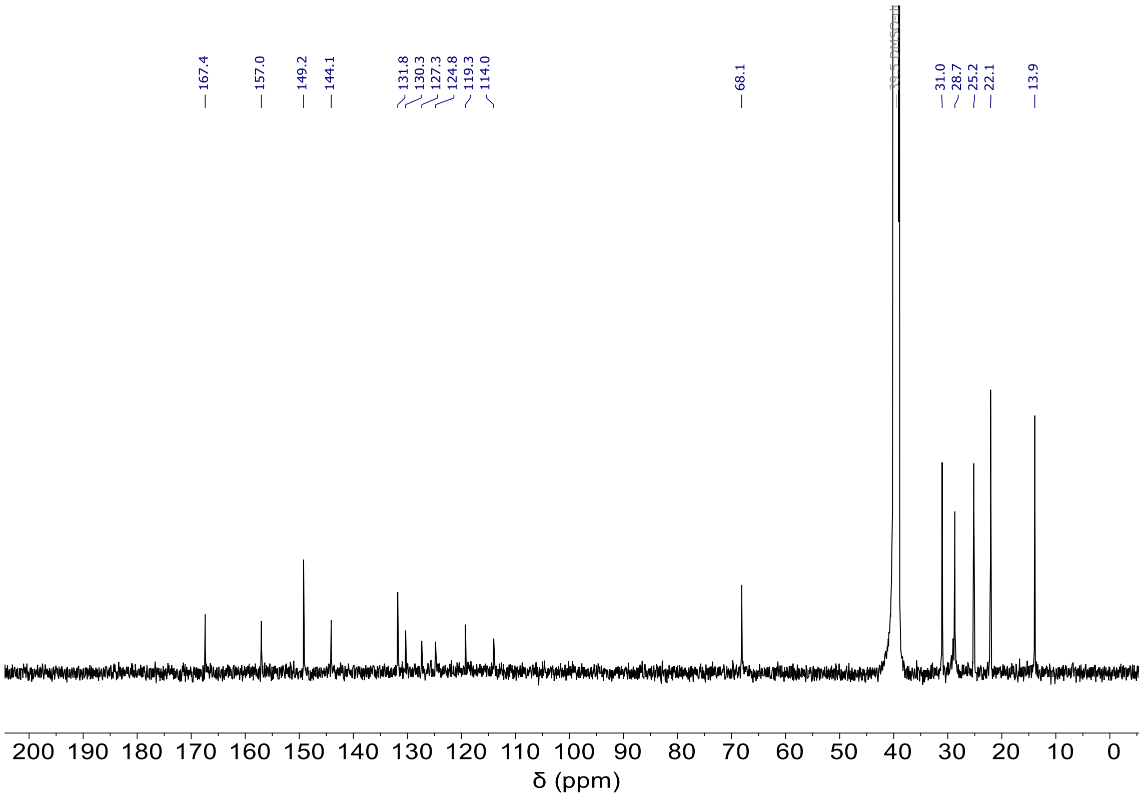

^{13}C NMR spectra of compound **P$_{Zn}$^{O}** in DMSO-D$_6$ (298K, 125 MHz).

Synthetic route to the A compound set.

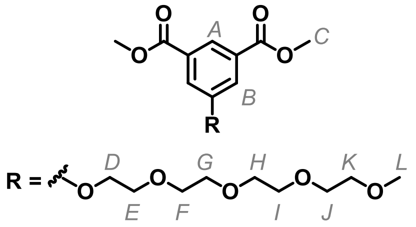

3[A]. To a solution of diester **1** (5.09 g, 24.23 mmol, 1 eq.) in DMF (60 mL), K_2CO_3 (6.70 g, 48.46 mmol, 2 eq.) and compound **2** (9.65 g, 26.66 mmol, 1.1 eq.) were added. The reaction mixture was heated at 80 ºC under stirring. The following day, the solvent was removed under reduced pressure with an air stream. The crude reaction mixture was redissolved in EtOAc and washed with H_2O (2 × 60 mL) and 5 M LiCl (5 × 50 mL). The organic phase was dried over $MgSO_4$, filtered, and the solvent was evaporated under reduced pressure, affording a yellow oil. Yield: 97% (9.36 g, 23.38 mmol).

^{1}H NMR (300 MHz, $CDCl_3$) δ (ppm) = 8.26 (t, *J* = 1.4 Hz, 1H, H^A), 7.76 (d, *J* = 1.4 Hz, 2H, H^B), 4.20 (t, *J* = 4.8 Hz, 2H, H^D), 3.93 (s, 6H, H^C), 3.88 (t, *J* = 4.8 Hz, 2H, H^E), 3.77-3.61 (m, 10H, H^{F-J}), 3.57-3.51 (m, 2H, H^K), 3.36 (s, 3H, H^L).

^{13}C NMR (75 MHz, $CDCl_3$) δ (ppm) = 166.1, 158.9, 131.7, 123.1, 119.9, 71.9, 70.9, 70.64, 70.64, 70.61, 70.5, 69.5, 68.1, 59.0, 52.4.

HRMS (**APCI+**): Calculated for $C_{19}H_{29}O_9$ $[M+H]^+$ = 401.1806, found at *m/z* = 401.1806.

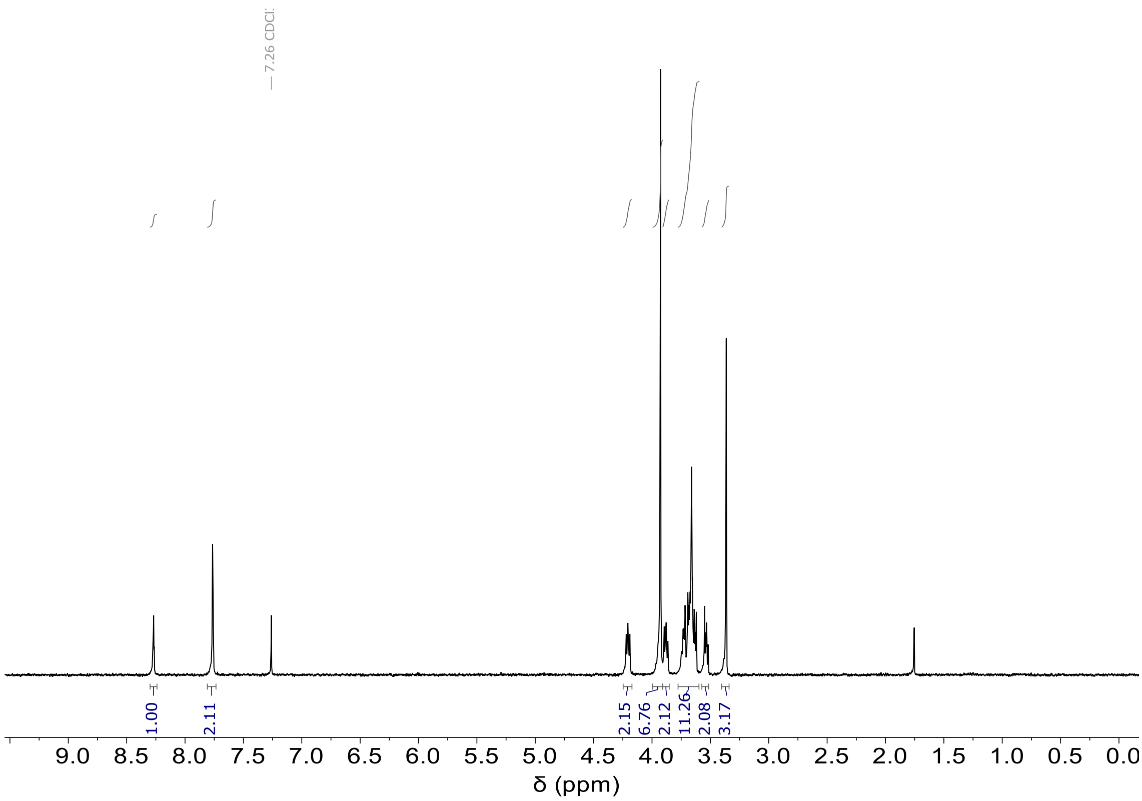

^{1}H NMR spectra of compound ***3***[A] in $CDCl_3$ (298K, 300 MHz).

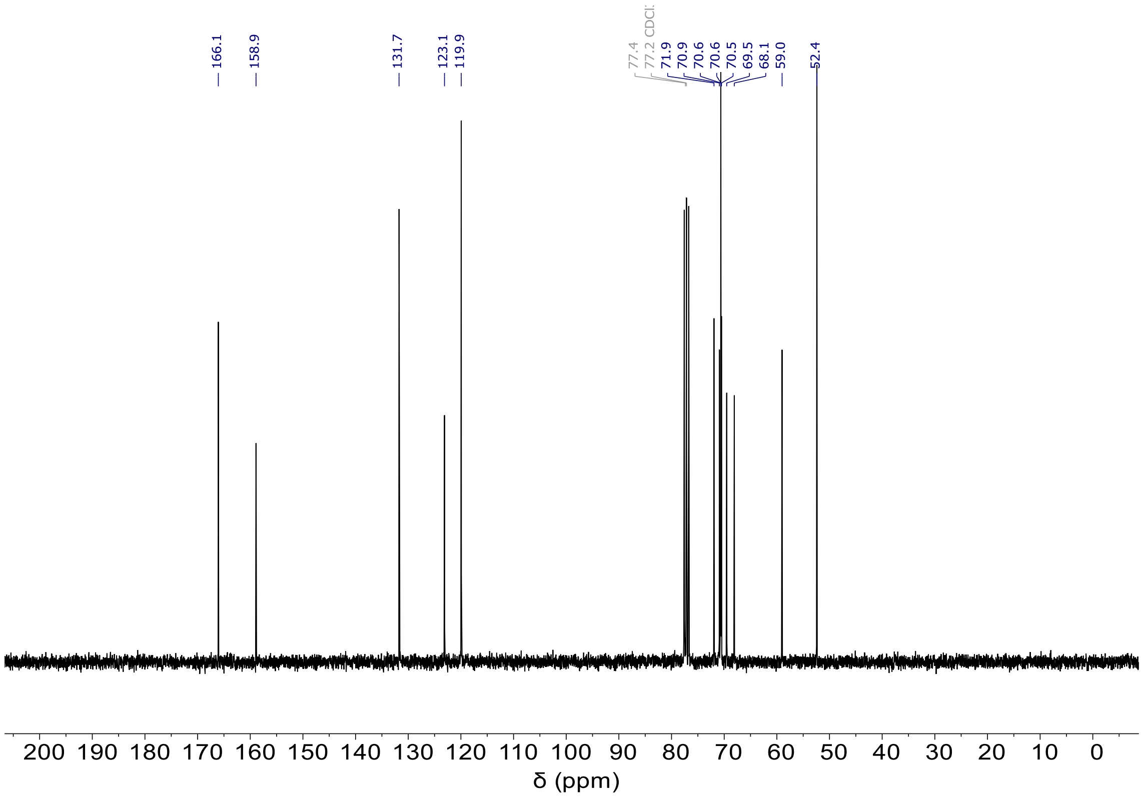

^{13}C NMR spectra of compound ***3***[A] in $CDCl_3$ (298K, 75 MHz).

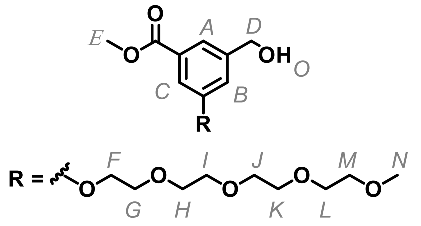

4[A]. Compound **3**[A] (3.11 g, 7.78 mmol, 1 eq.) was dissolved in anhydrous THF (17 mL) under an inert atmosphere. Then, 2 M $LiBH_4$ solution in THF (2.3 mL, 4.67 mmol, 0.6 eq.) was added. After stirring overnight, $LiBH_4$ was quenched at 0 ºC by adding EtOAc (10 mL), H_2O (10 mL), and 2 M HCl (3 mL). The mixture was extracted with EtOAc (3 × 30 mL), and the organic phase washed with 2 M HCl (1 × 25 mL) and H_2O (1 × 25 mL). The organic phase was dried over $MgSO_4$, filtered, and the solvent was removed under reduced pressure. The crude was purified by silica gel column chromatography (cyclohexane/EtOAc 20:80), affording a yellow oil. Yield: 34% (0.98 g, 2.62 mmol).

^{1}H NMR (300 MHz, $CDCl_3$) δ (ppm) = 7.61-7.59 (m, 1H, H^A), 7.49-7.46 (sa, 1H, H^C), 7.20-7.16 (m, 1H, H^B), 4.69 (d, J = 5.7 Hz, 2H, H^D), 4.18 (t, J = 5.5 Hz, 2H, H^F), 3.93-3.82 (m, 5H, $H^{E,\ G}$), 3.75-3.58 (m, 10H, $H^{H\text{-}L}$), 3.56-3.49 (m, 2H, H^M), 3.36 (s, 3H, H^N).

^{13}C NMR (75 MHz, $CDCl_3$) δ (ppm) = 166.7, 158.7, 143.3, 131.0, 120.1, 117.8, 113.6, 71.7, 70.6, 70.38, 70.36, 70.34, 70.2, 69.4, 67.5, 63.9, 58.7, 52.0.

HRMS (**APCI+**): Calculated for $C_{18}H_{29}O_8$ $[M+H]^+$ = 373.1857, found at m/z = 373.1851.

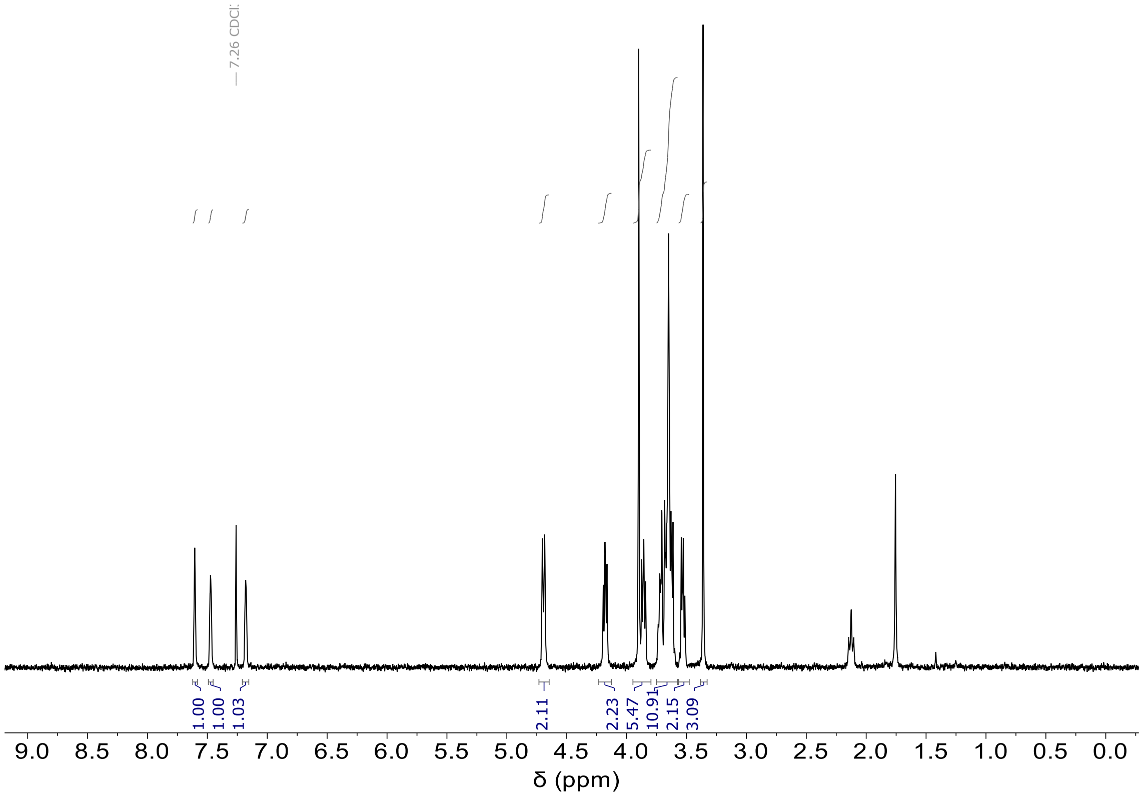

^{1}H NMR spectra of compound ***4***[A] in CDCl3 (298K, 300 MHz).

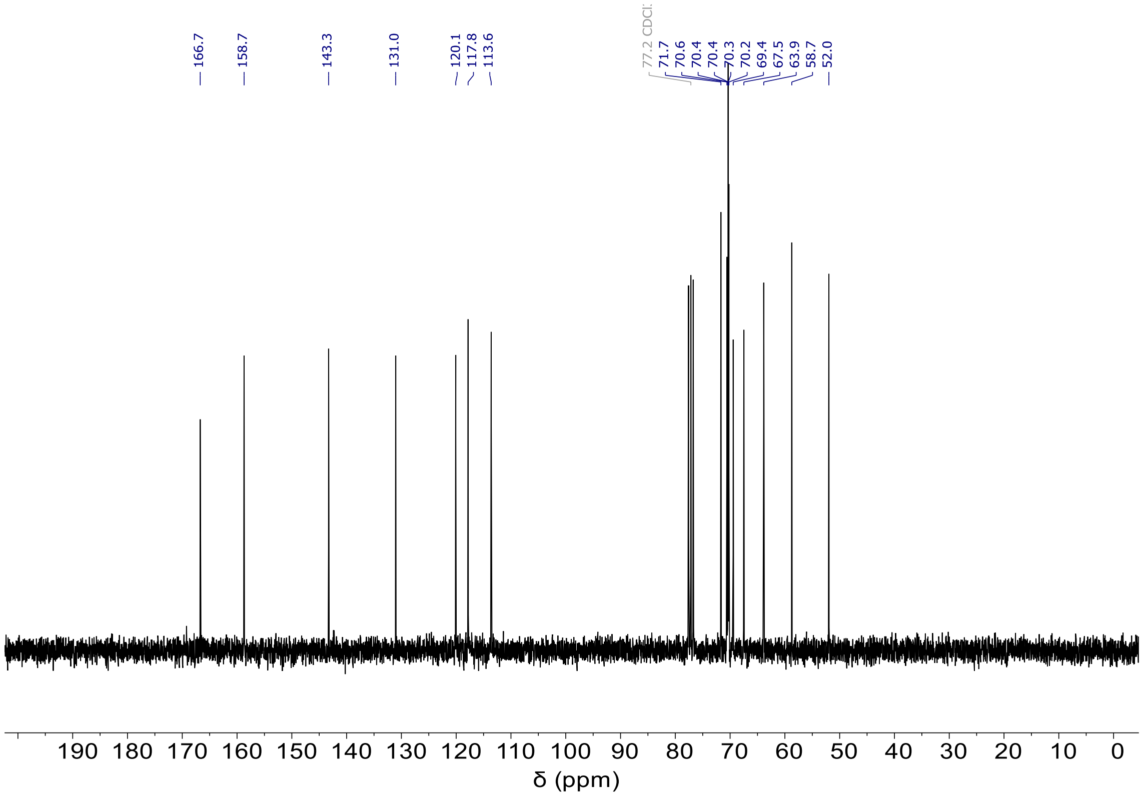

^{13}C NMR spectra of compound ***4***[A] in CDCl3 (298K, 75 MHz).

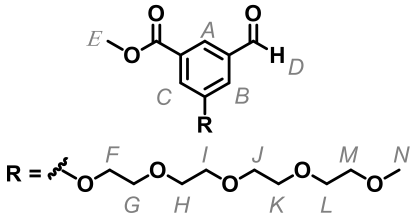

5[A]. To a solution of compound **4**[A] (2.40 g, 6.49 mmol, 1 eq.) in CH_2Cl_2 (65 mL), pyridinium chlorochromate (2.10 g, 9.73 mmol, 1.5 eq.) and a spoon of celite were added. The reaction mixture was stirred at room temperature for three hours. The resulting suspension was filtered through a silica plug using EtOAc as eluent. The filtrate was purified by silica gel column chromatography (cyclohexane/EtOAc 20:80), affording a yellowish oil. Yield: 92% (2.21 g, 5.98 mmol).

^{1}H NMR (300 MHz, $CDCl_3$) δ (ppm) = 10.01 (s, 1H, H^D), 8.11 (dd, J = 1.5, 1.3 Hz, 1H, H^A), 7.85 (dd, J = 2.6, 1.5 Hz, 1H, H^C), 7.60 (dd, J = 2.6, 1.3 Hz, 1H, H^B), 4.23 (t, J = 5.2 Hz, 2H, H^F), 3.95 (s, 3H, H^E), 3.90 (t, J = 5.2 Hz, 2H, H^G), 3.76-3.60 (m, 10H, $H^{H\text{-}L}$), 3.57-3.51 (m, 2H, H^M), 3.37 (s, 3H, H^N).

^{13}C NMR (75 MHz, $CDCl_3$) δ (ppm) = 190.9, 165.5, 159.3, 137.6, 132.2, 123.9, 121.7, 121.6, 117.5, 71.7, 70.7, 70.39, 70.35, 70.2, 69.2, 68.0, 58.7, 52.3.

HRMS (**APCI+**): Calculated for $C_{18}H_{27}O_8$ $[M+H]^+$ = 371.1700, found at m/z = 371.1702.

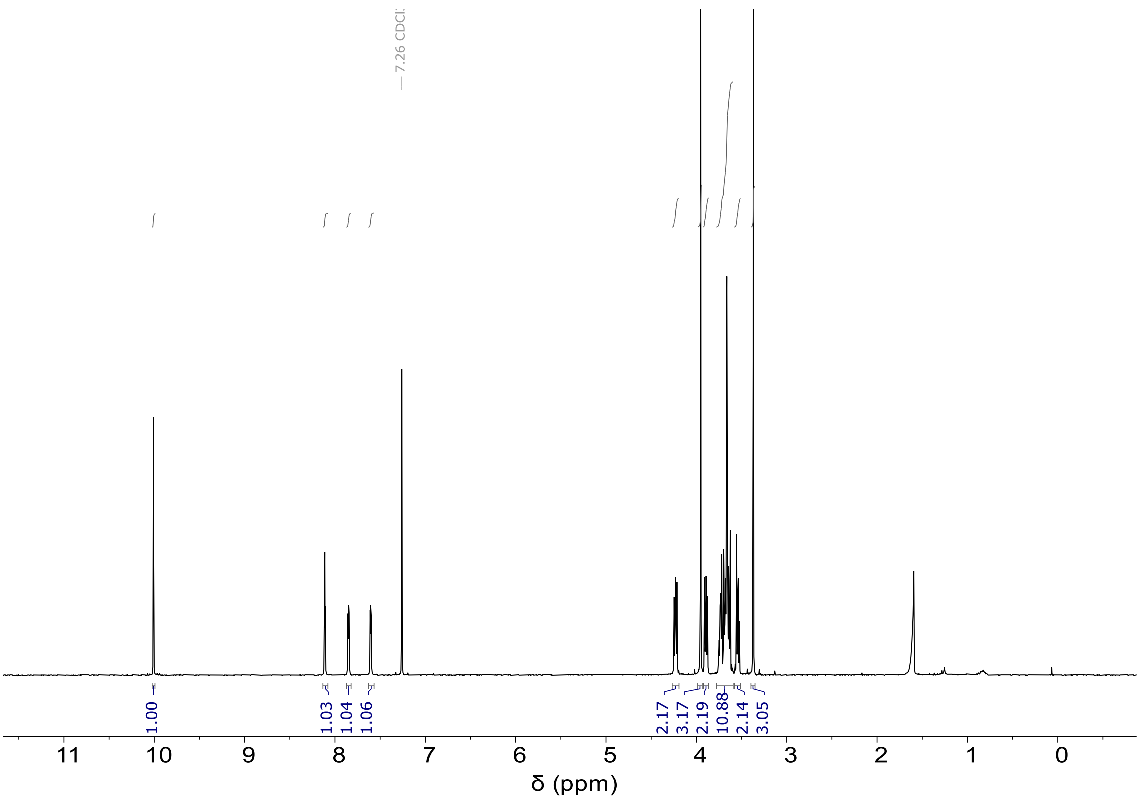

^{1}H NMR spectra of compound ***5***[A] in $CDCl_3$ (298K, 300 MHz).

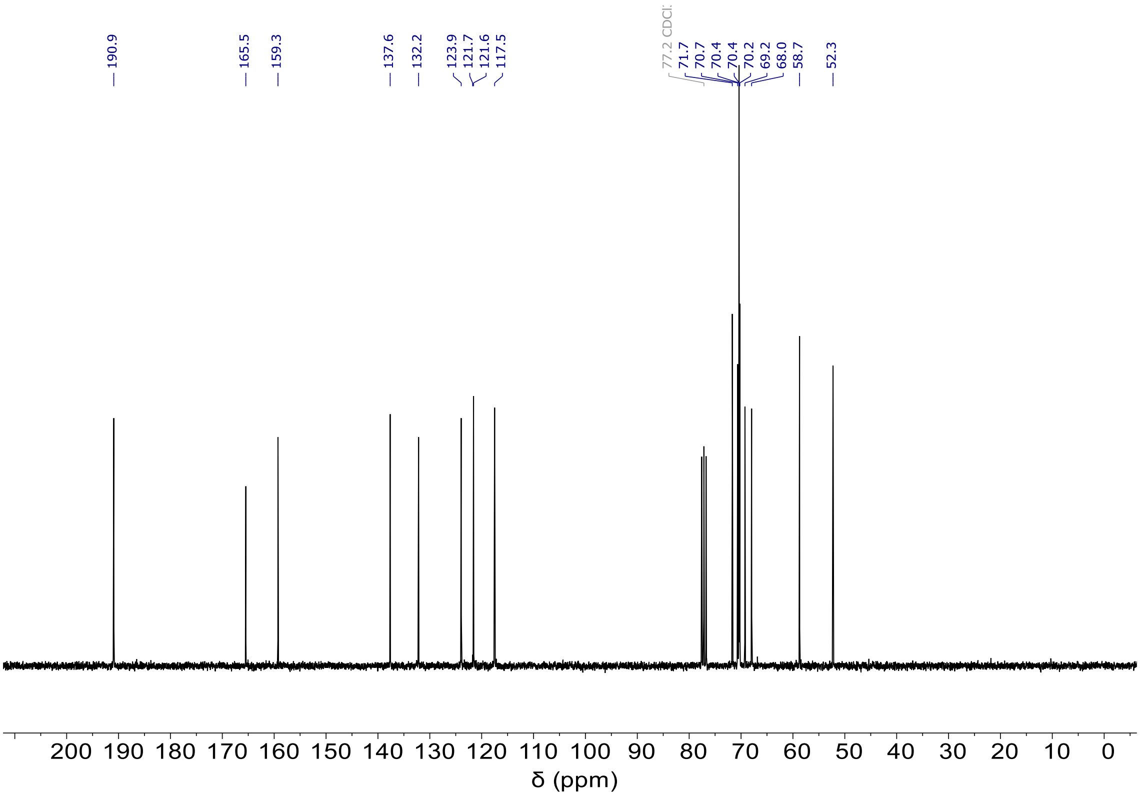

^{13}C NMR spectra of compound ***5***[A] in $CDCl_3$ (298K, 75 MHz).

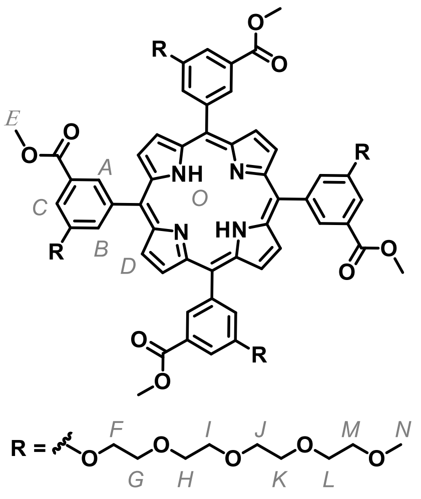

6[A]. Aldehyde **5**[A] (3.35 g, 9.04 mmol, 1 eq.) was dissolved in propionic acid (30 mL) under an inert atmosphere. Upon heating at 145 ºC, pyrrole (0.63 mL, 9.04 mmol, 1 eq.) was added. The reaction mixture was stirred at this temperature for three hours, protected from light. Then, the heat source and the inert atmosphere were removed, and stirring was continued overnight at room temperature. The reaction mixture was dissolved in EtOAc and then washed with a saturated $NaHCO_3$ solution (3 × 50 mL). The organic phase was dried over Na_2SO_4, filtered, and the solvent was removed under reduced pressure. The crude was purified twice by silica gel column chromatography ($CHCl_3$/MeOH 100:0 to 20:1 and cyclohexane/THF 1:1 to 0:100). The product was then precipitated in diisopropyl ether at 0 ºC, and the resulting suspension was filtered through a filter plate and washed with cyclohexane. Finally, the solid was filtered through a silica plug ($CHCl_3$/MeOH 100:0 to 10:1), and the solvent was removed under reduced pressure. A purple solid was obtained. Yield: 16% (0.60 g, 0.36 mmol).

^{1}H NMR (300 MHz, $CDCl_3$) δ (ppm) = 8.82 (s, 8H, H^D), 8.50-8.46 (m, 4H, H^A), 8.06-8.01 (m, 4H, H^B), 8.00-7.96 (m, 4H, H^C), 4.43-4.35 (m, 8H, H^F), 4.02-3.92 (m, 20H, $H^{E,\ G}$), 3.81-3.40 (m, 12H, $H^{H\text{-}M}$), 3.29 (s, 12H, H^N), -2.86 (s, 2H, H^O).

^{13}C NMR (75 MHz, $CDCl_3$) δ (ppm) = 167.2, 157.5, 143.5, 131.4 (pyrr), 129.9, 128.3, 126.0, 119.1, 114.7, 77.4, 72.0, 71.1, 70.8, 70.71, 70.67, 70.56, 69.9, 68.3, 59.1, 52.5.

HRMS (**MALDI**, matrix: DCTB): Calculated for $C_{88}H_{110}N_4O_{28}$ $[M]^+$ = 1671.7334, found at *m/z* = 1671.7257.

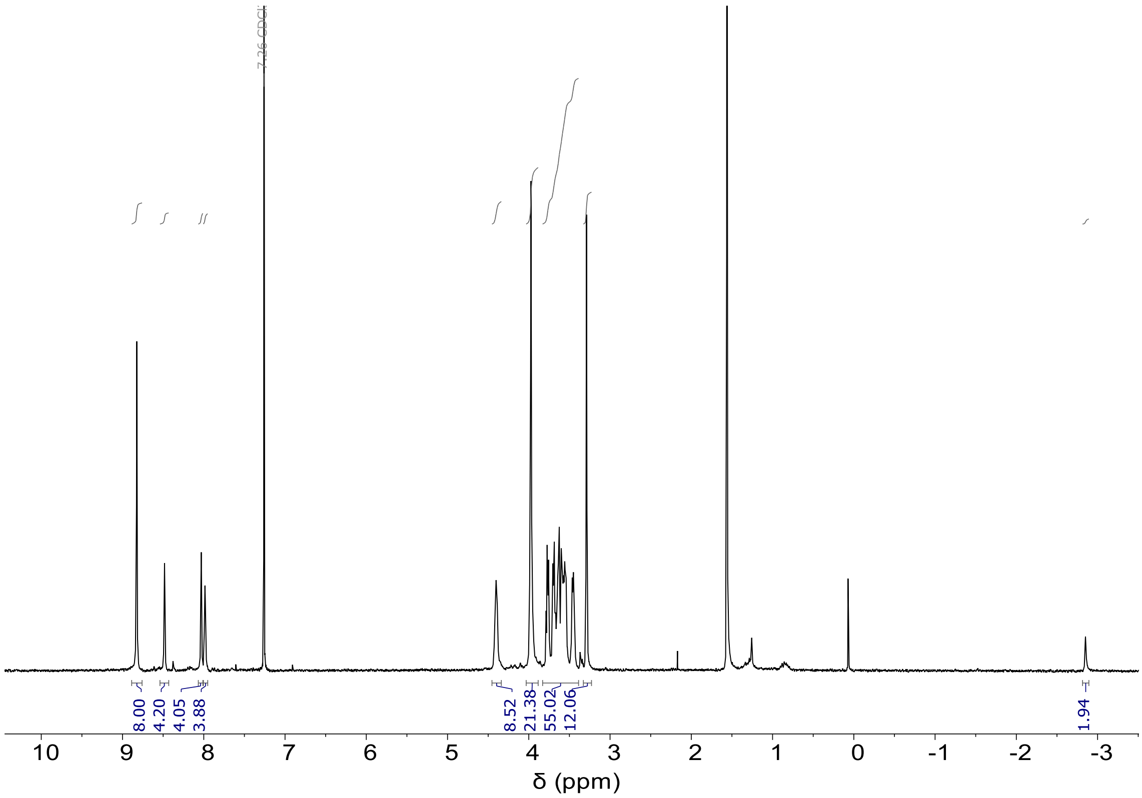

^{1}H NMR spectra of compound ***6***[A] in $CDCl_3$ (298K, 300 MHz).

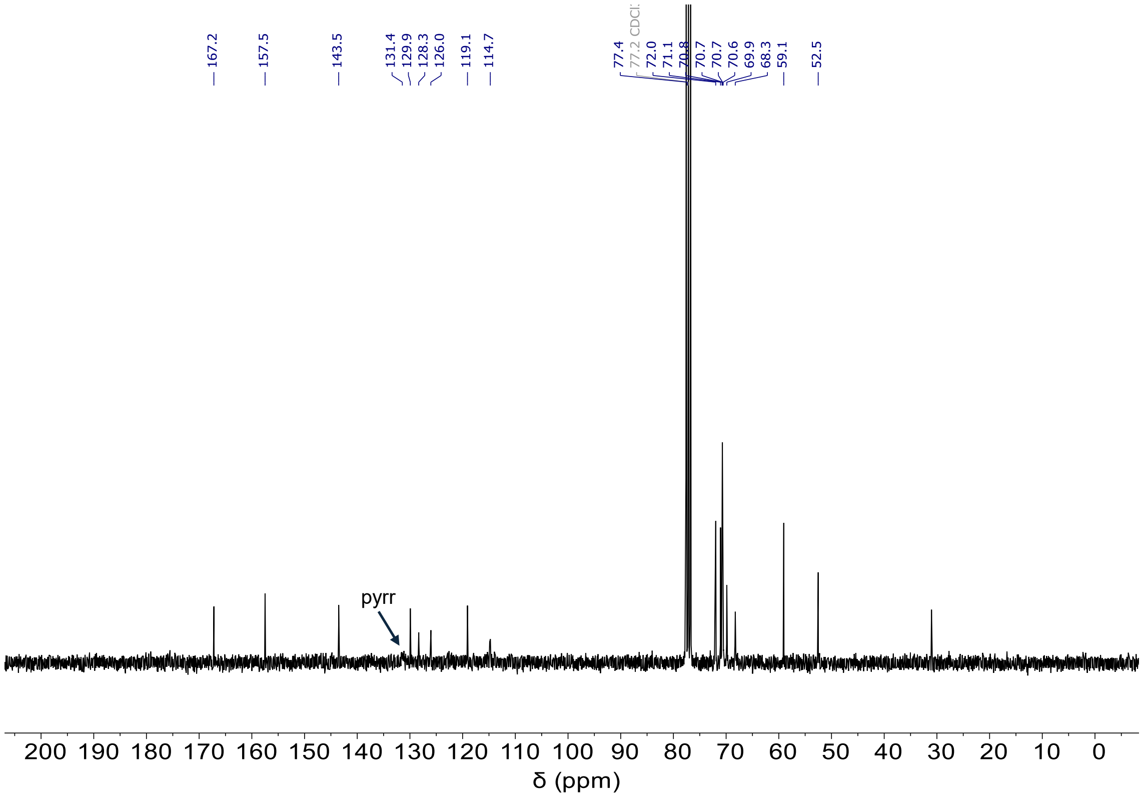

^{13}C NMR spectra of compound ***6***[A] in $CDCl_3$ (298K, 75 MHz).

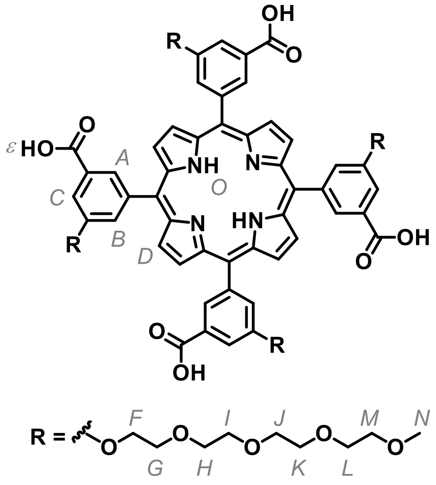

P_{2H}[A]. A solution of tetraester porphyrin **6**[A] (0.25 g, 0.15 mmol, 1 eq.) in THF (15 mL) was added to a solution of KOH (0.84 g, 14.95 mmol, 100 eq.) in H_2O (2 mL). The mixture was heated to reflux overnight under magnetic stirring, resulting in the formation of an insoluble purple precipitate. The solvent was removed by decantation, and the precipitate was sonicated in $CHCl_3$ to disperse the aggregate. The resulting suspension was then extracted with acidified H_2O (adjusted to pH 4 with HCl) and $CHCl_3$ until a colorless aqueous phase was obtained. The organic phase was dried over Na_2SO_4, filtered, and the solvent was removed under reduced pressure. A purple solid was obtained. Yield: 98% (0.24 g, 0.15 mmol).

^{1}H NMR (500 MHz, DMSO-D_6) δ (ppm) = 13.31 (s, 4H, H^{ε}), 8.89 (s, 8H, H^D), 8.29 (m, 4H, H^A), 8.07 (m, 4H, H^B), 7.93 (m, 4H, H^C), 4.39 (m, 8H, H^F), 3.85 (m, 8H, H^G), 3.65-3.40 (m, 48H, $H^{H\text{-}M}$), 3.08 (m, 12H, H^N), -2.96 (s, 2H, H^O).

^{13}C NMR (75 MHz, DMSO-D_6) = δ (ppm) = 167.2, 157.2, 142.6, 131.3 (pyrr), 130.7, 127.5, 124.9, 123.0, 118.9, 71.1, 70.0, 69.80, 69.76, 69.69, 69.45, 69.44, 69.0, 68.0, 57.9.

HRMS (**MALDI**, matrix: DHB): Calculated for $C_{84}H_{103}N_4O_{28}$ $[M+H]^+$ = 1615.6753, found at *m/z* = 1615.6717.

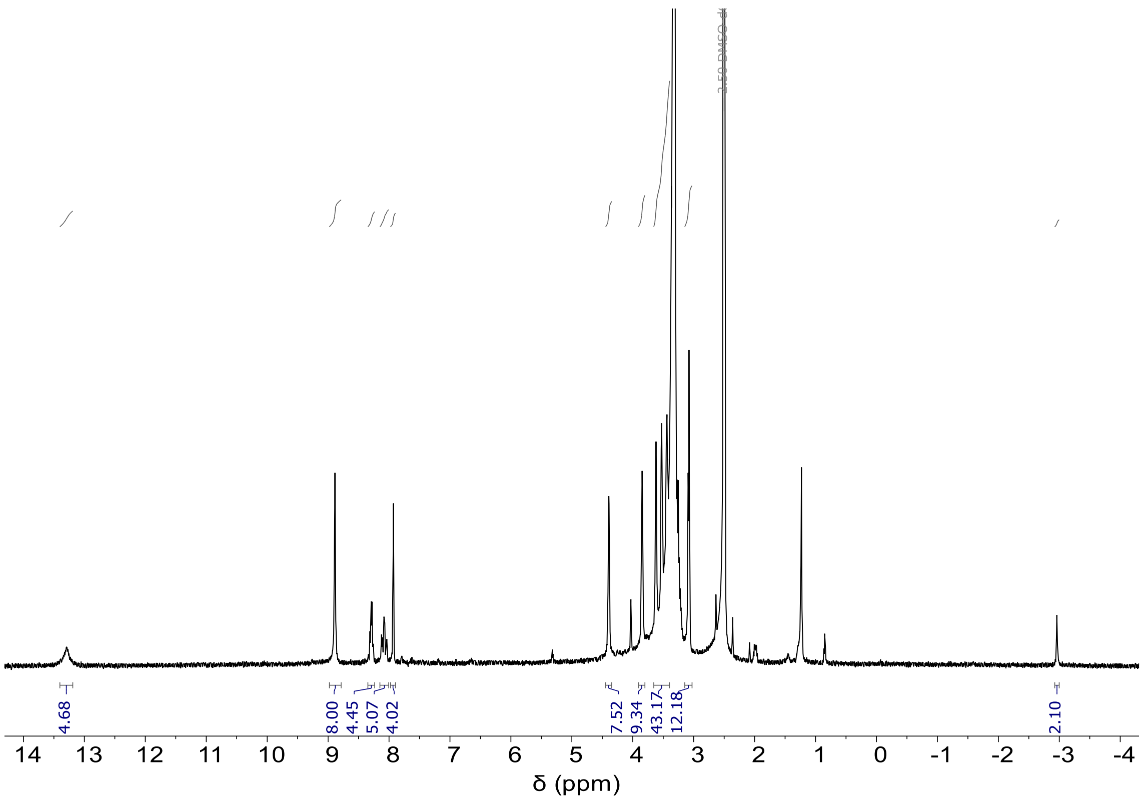

^{1}H NMR spectra of compound $\boldsymbol{P_{2H}}^{A}$ in DMSO-D6 (298K, 500 MHz).

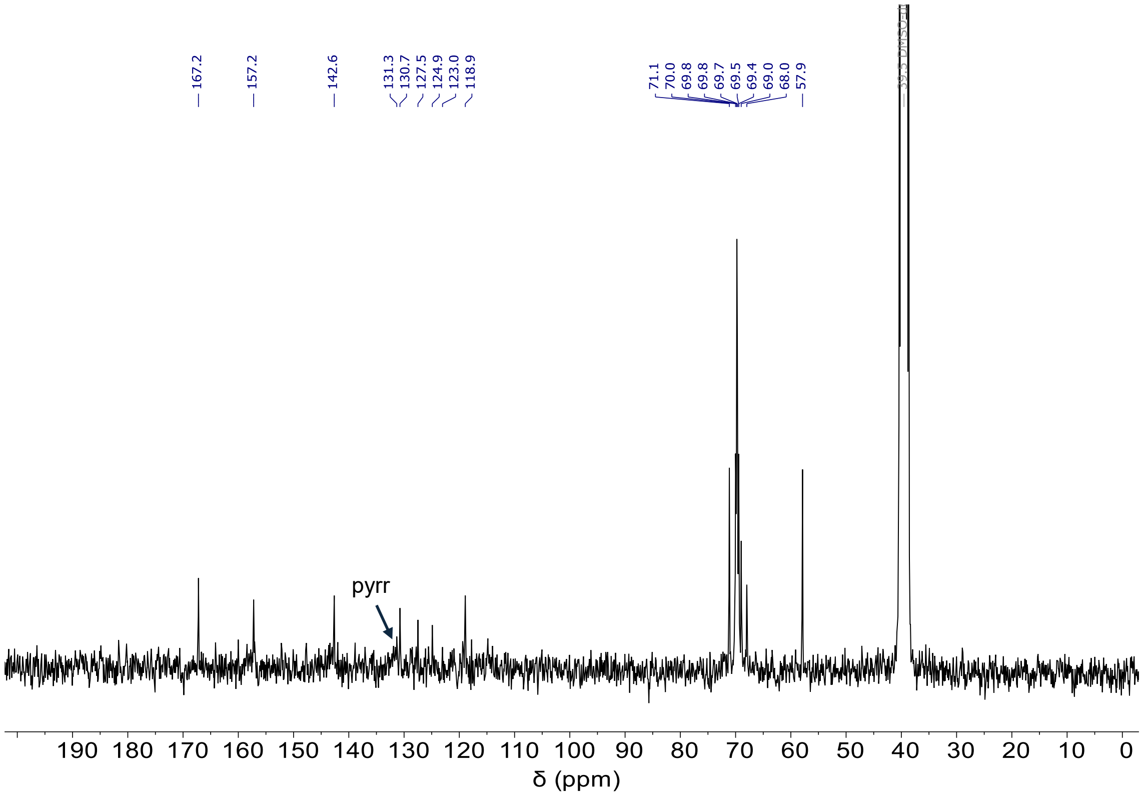

^{13}C NMR spectra of compound $\boldsymbol{P_{2H}}^{A}$ in DMSO-D6 (298K, 75 MHz).

Synthesis of L^O and L^A

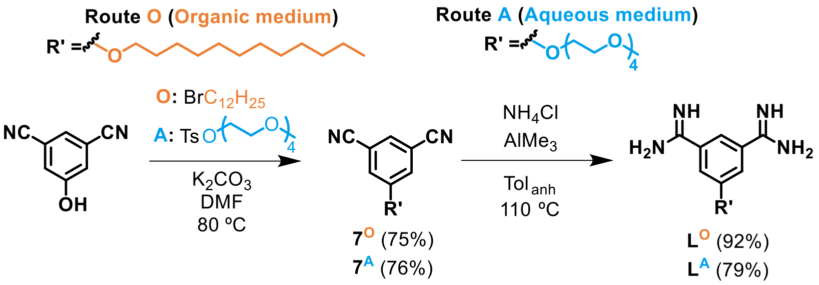

Scheme S3. Synthetic routes **O** (organic) and **A** (aqueous) to prepare the corresponding diamidine linker.

Synthetic route to the O compound set.

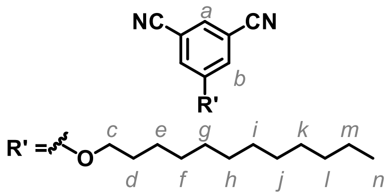

7°. Commercially available 5-hydroxyisophthalonitrile (1.00 g, 6.94 mmol, 1 eq.) was dissolved in DMF (50 mL) and K_2CO_3 (1.92 g, 13.88 mmol, 2 eq.) was added. Then, 1-bromododecane (2.59 g, 10.41 mmol, 1.5 eq.) was injected and the mixture was stirred at 80 ºC overnight. The solvent was removed under reduced pressure with an air stream, and the crude was redissolved in EtOAc and washed with H_2O (30 mL × 3), and 5 M LiCl (20 mL × 5). The organic phase was dried over $MgSO_4$, filtered, and the solvent was removed under reduced pressure. The crude product was purified by silica gel column chromatography (cyclohexane/EtOAc 80:20), affording compound **7°** as a white solid. Yield: 75% (1.62 g, 5.18 mmol).

^{1}H NMR (300 MHz, DMSO-D_6) δ (ppm) = 7.99 (m, 1H, H^a), 7.80 (m, 2H, H^b), 4.08 (t, J = 6.5 Hz, 2H, H^c), 1.71 (m, 2H, H^d), 1.44-1.17 (m, 18H, H^{e-m}), 0.85 (m, 3H, H^n).

^{13}C NMR (75 MHz, DMSO-D_6) δ (ppm) = 159.1, 127.7, 123.0, 117.0, 113.8, 68.9, 31.3, 29.00, 28.99, 28.94, 28.93, 28.7, 28.6, 28.2, 25.2, 22.1, 13.9.

HRMS (**APCI+**): Calculated for $C_{20}H_{29}N_2O$ $[M+H]^+$ = 313.2274, found at m/z = 313.2272.

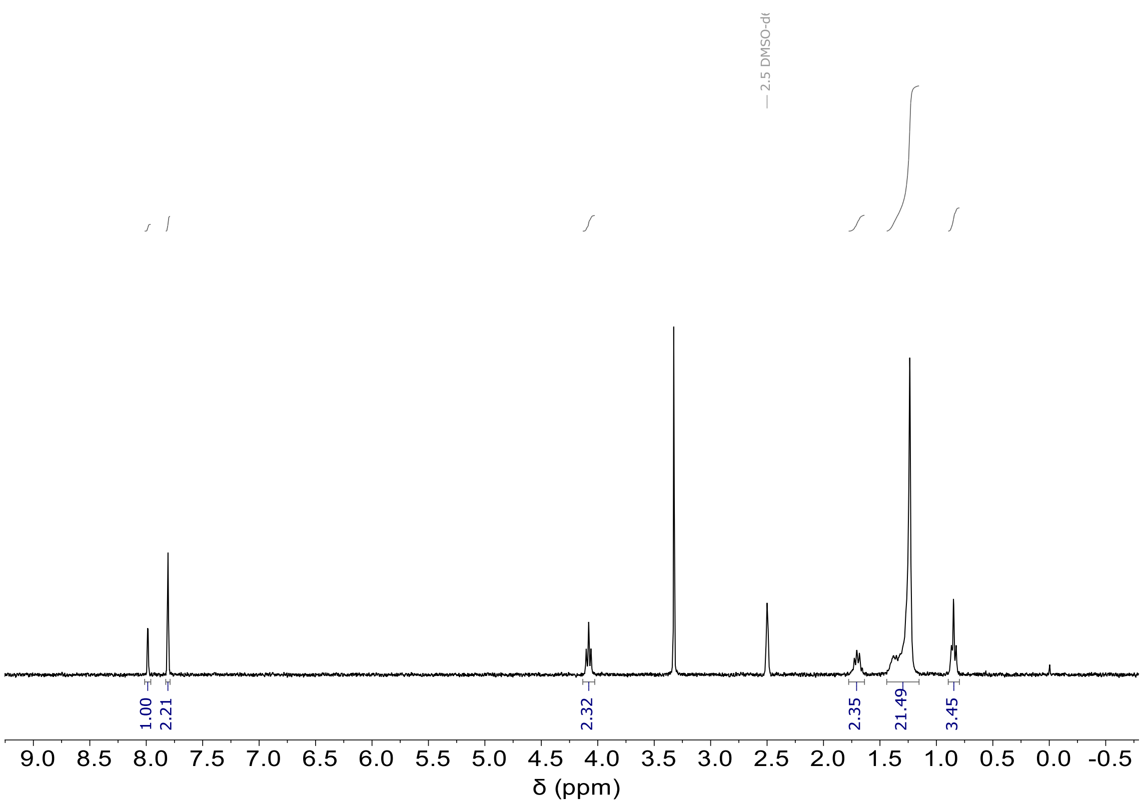

^{1}H NMR spectra of compound **7**[O] in DMSO-D_6 (298K, 300 MHz).

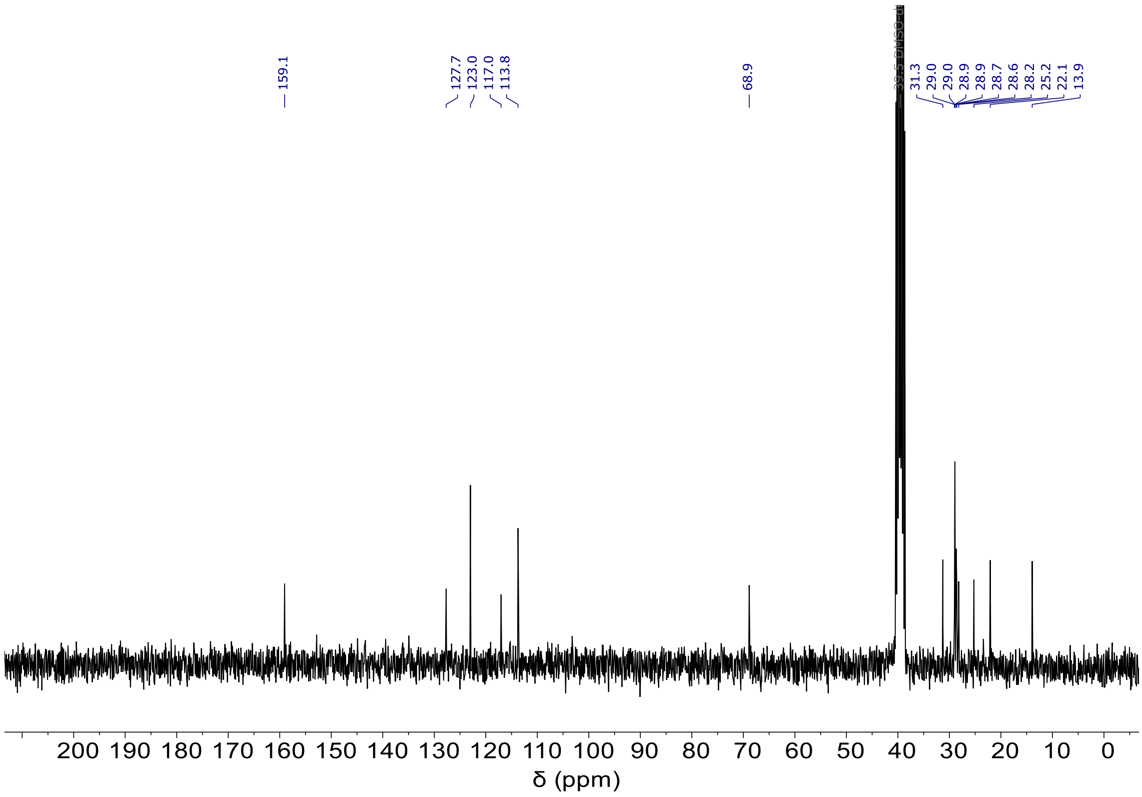

^{13}C NMR spectra of compound **7**[O] in DMSO-D_6 (298K, 75 MHz).

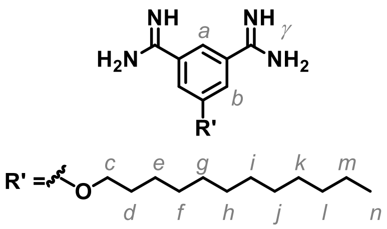

L[O]. In a self-sealing vial (previously dried), NH_4Cl (1.36 g, 25.37 mmol, 15 eq.) was added, and vacuum/argon cycles (×3) were performed. A 2 M solution of $AlMe_3$ in toluene (13 mL, 24.37 mmol, 7 eq.) was added at 0 ºC. After stirring for 30 minutes, compound **7**[O] (1.32 g, 4.23 mmol, 1 eq.) dissolved in anhydrous toluene (11 mL) was added at room temperature. 15 minutes later, the reaction mixture was heated to 110 ºC until complete conversion, as confirmed by ^1H NMR. Cold MeOH (50 mL) was then added at 0 ºC, and the mixture was filtered through a celite plug, washing with MeOH. The solvent from the filtrate was removed under reduced pressure. Next, the residue was redissolved in a 0.3 M NaOH solution and extracted repeatedly with butanol (1 mL portions) until no compound was detected by TLC. The organic phase was dried over $MgSO_4$, filtered, and the solvent was removed under reduced pressure. White solid. Yield: 92% (1.35 g, 3.90 mmol).

^{1}H NMR (500 MHz, DMSO-D_6) δ (ppm) = 9.54 (broad s, 6H, H^{γ}), 8.09 (m, 1H, H^a), 7.71 (d, J = 1.5z Hz, 2H, H^b), 4.16 (m, J = 6.4z Hz, 2H, H^c), 1.76 (m, 2H, H^d), 1.49-1.17 (m, 18H, $H^{e\text{-}m}$), 0.85 (t, J = 6.7z Hz, 3H, H^n).

^{13}C NMR (75 MHz, DMSO-D_6) δ (ppm) = 176.9, 163.3, 158.5, 134.5, 118.1, 115.8, 68.0, 31.3, 29.1, 29.0, 28.8, 28.7, 28.6, 25.5, 25.0, 22.1, 13.9.

HRMS (**APCI+**): Calculated for $C_{20}H_{35}N_4O$ $[M+H]^+$ = 347.2811, found at m/z = 347.2811.

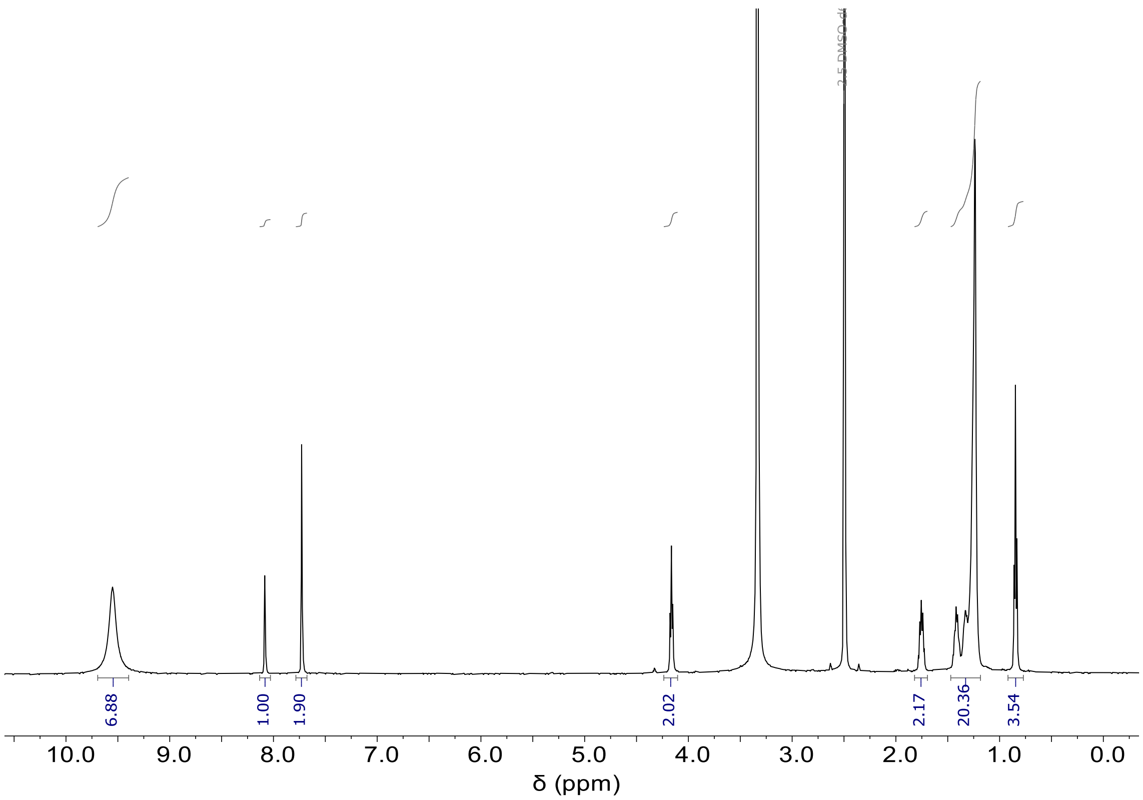

^{1}H NMR spectra of compound **L**^{O} in DMSO-D$_6$ (298K, 500 MHz).

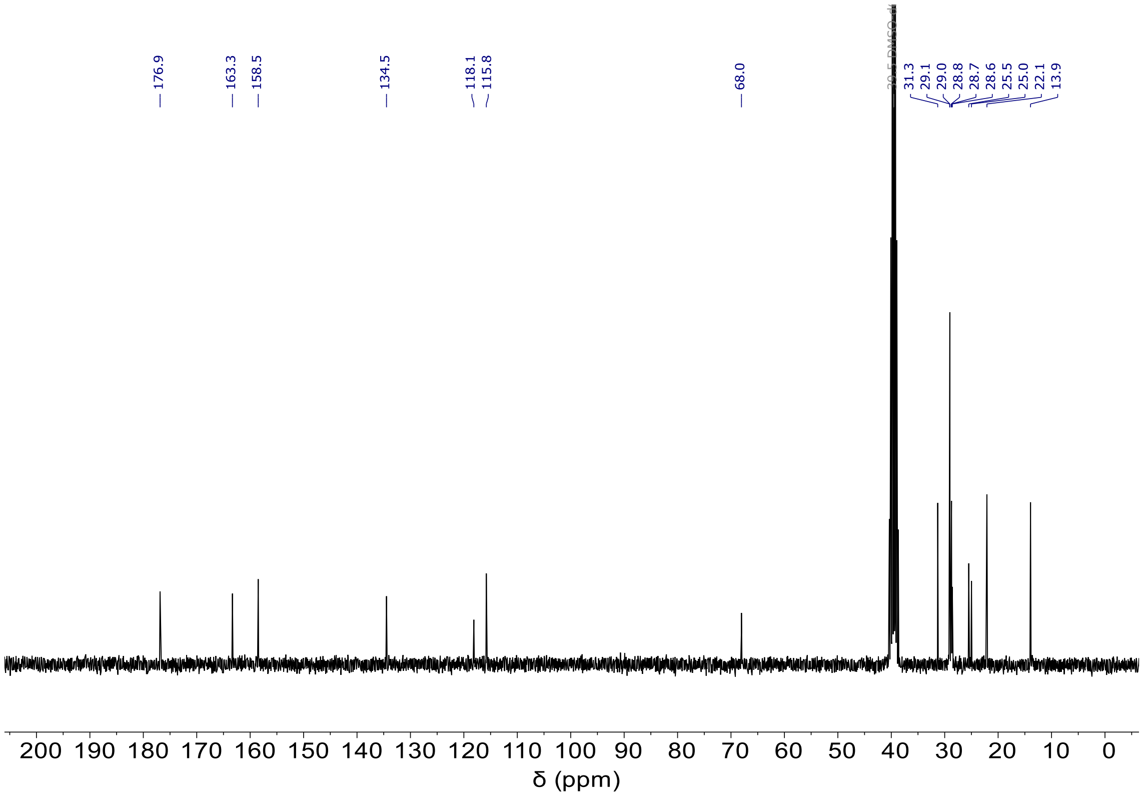

^{13}C NMR spectra of compound **L**^{O} in DMSO-D$_6$ (298K, 75 MHz).

Synthetic route to the A compound set.

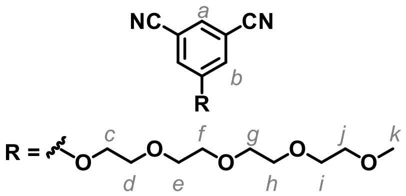

7[A]. Commercial 5-hydroxyisophthalonitrile (1.00 g, 6.94 mmol, 1 eq.) was dissolved in DMF (50 mL), and K_2CO_3 (1.92 g, 13.88 mmol, 2 eq.) was added. Then, tosylate derivative **2** (2.26 g, 6.24 mmol, 0.9 eq.) was incorporated into the reaction mixture, which was stirred overnight at 80 ºC. The solvent was removed under reduced pressure with an air stream, and the crude was redissolved in EtOAc and washed with H_2O (40 mL × 3). The organic phase was dried over $MgSO_4$, filtered, and the solvent was removed under reduced pressure. The crude was purified by silica gel column chromatography (cyclohexane/EtOAc 100:0 to 20:80), affording compound **7[A]** as a yellow oil. Yield: 76% (1.75 g, 5.25 mmol).

^{1}H NMR (300 MHz, $CDCl_3$) δ (ppm) = 7.51 (t, *J* = 1.4 Hz, 1H, H^a), 7.42 (d, *J* = 1.4 Hz, 2H, H^b), 4.19 (m, 2H, H^c), 3.88 (m, 2H, H^d), 3.75-3.60 (m, 12H, $H^{e\text{-}j}$), 3.55 (m, 3H, H^k).

^{13}C NMR (75 MHz, DMSO-D_6) δ (ppm) = 158.9, 127.9, 123.1, 117.0, 113.7, 71.3, 69.9, 69.81, 69.78, 69.77, 69.6, 68.54, 68.46, 58.0.

HRMS (**APCI+**): Calculated for $C_{17}H_{23}N_2O_5$ $[M+H]^+$ = 335.1601, found at *m/z* = 335.1606.

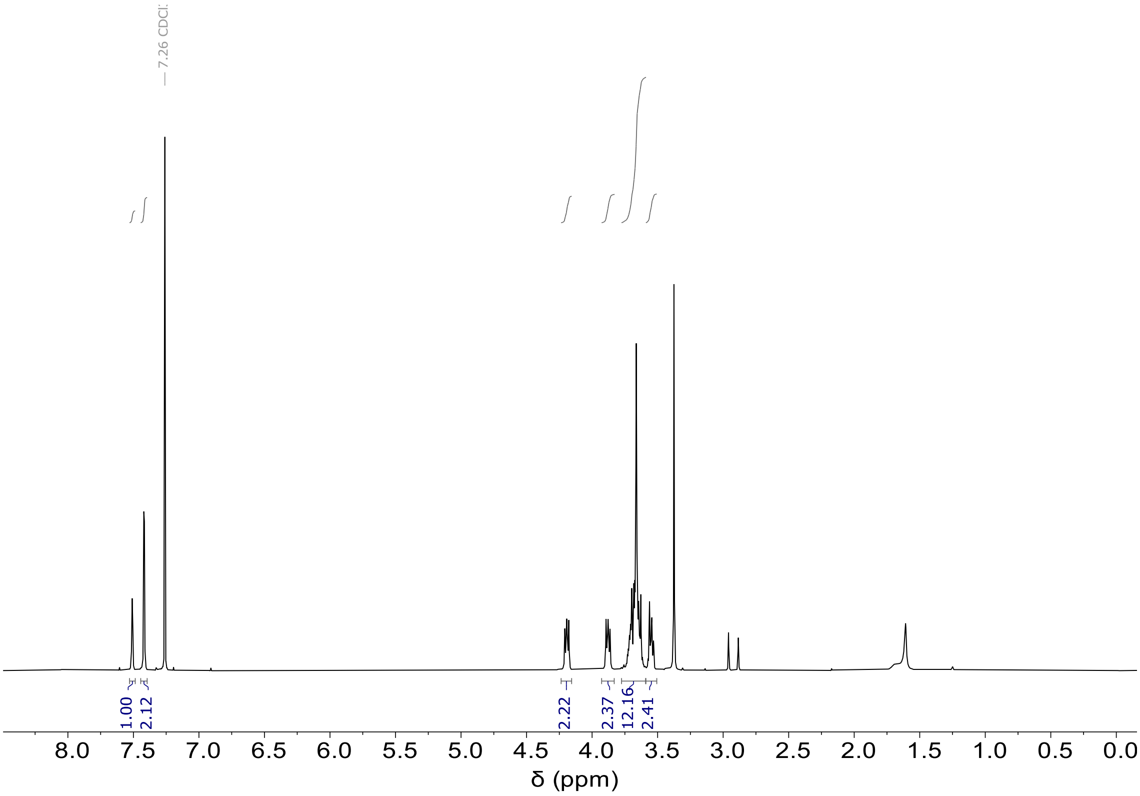

^{1}H NMR spectra of compound **7**[A] in $CDCl_3$ (298K, 300 MHz).

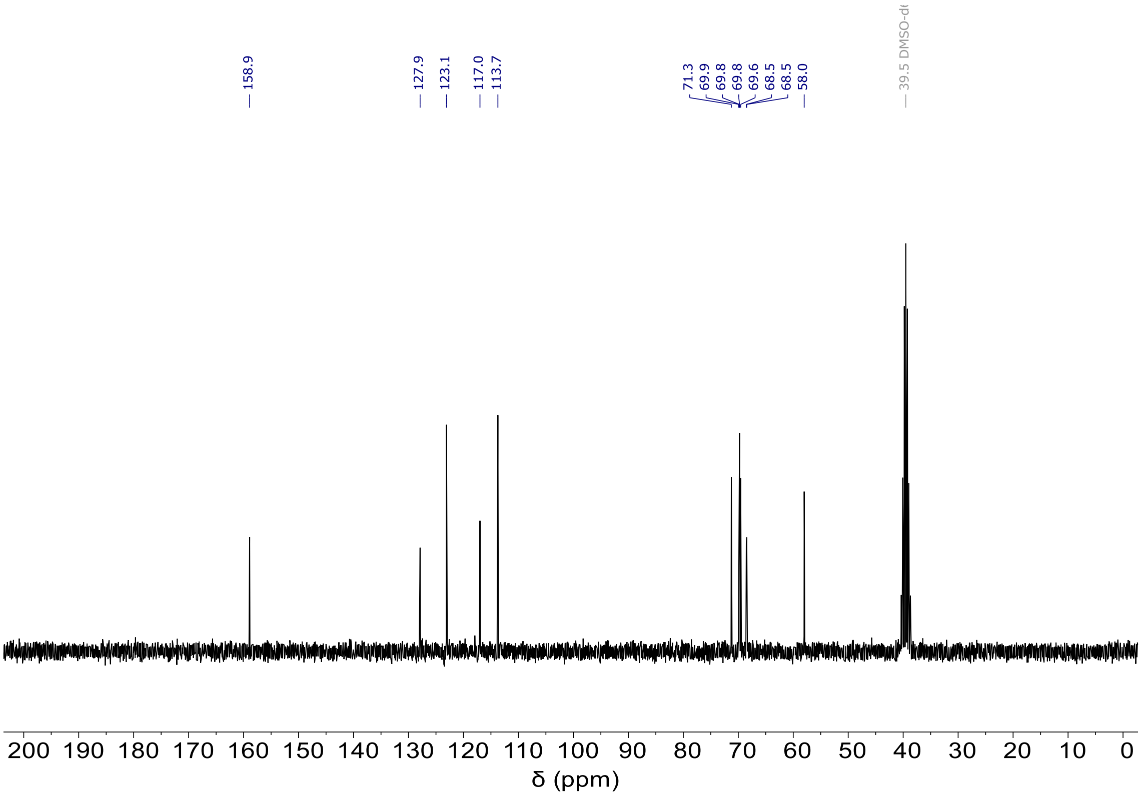

^{13}C NMR spectra of compound **7**[A] in DMSO-D_6 (298K, 75 MHz).

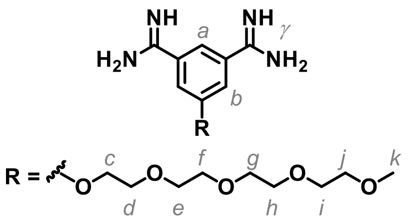

L[A]. In a self-sealing vial (previously oven dried), NH_4Cl (0.49 g, 9.17 mmol, 15 eq.) was added, and vacuum/argon cycles (×3) were performed. At 0 ºC, a 2 M solution of $AlMe_3$ in toluene (1.8 mL, 3.67 mmol, 6 eq.) was added. After 30 minutes stirring, compound **7**[A] (0.20 g, 0.61 mmol, 1 eq.) dissolved in anhydrous toluene (2.6 mL) was added at room temperature. After 15 minutes, the reaction mixture was heated to 110 ºC until complete conversion, as confirmed by ^{1}H NMR. Cold MeOH (15 mL) was then added at 0 ºC, and the mixture was filtered through a celite plug, washing with MeOH. The solvent from the filtrate was removed under reduced pressure. Next, the residue was redissolved in a 0.3M NaOH solution and extracted repeatedly with butanol (1 mL portions) until no compound was detected by TLC. The organic phase was dried over $MgSO_4$, filtered, and the solvent was removed under reduced pressure. White solid. Yield: 79% (0.18 g, 0.48 mmol).

^{1}H NMR (300 MHz, DMSO-D_6) δ (ppm) = 9.51 (m, 6H, $H^γ$), 7.82 (t, J = 1.7 Hz, 1H, H^a), 7.71 (d, J = 1.7 Hz, 2H, H^b), 4.29 (m, 2H, H^c), 3.80 (m, 2H, H^d), 3.64-3.37 (m, 15H, H^{e-k}).

^{13}C NMR (125 MHz, DMSO-D_6) δ (ppm) = 162.7, 158.1, 135.7, 117.9, 115.1, 71.3, 69.9, 69.82, 69.82, 69.77, 69.6, 68.8, 67.5, 58.0.

HRMS (**ESI+**): Calculated for $C_{17}H_{29}N_4O_5$ $[M+H]^+$ = 369.2132, found at m/z = 369.2128.

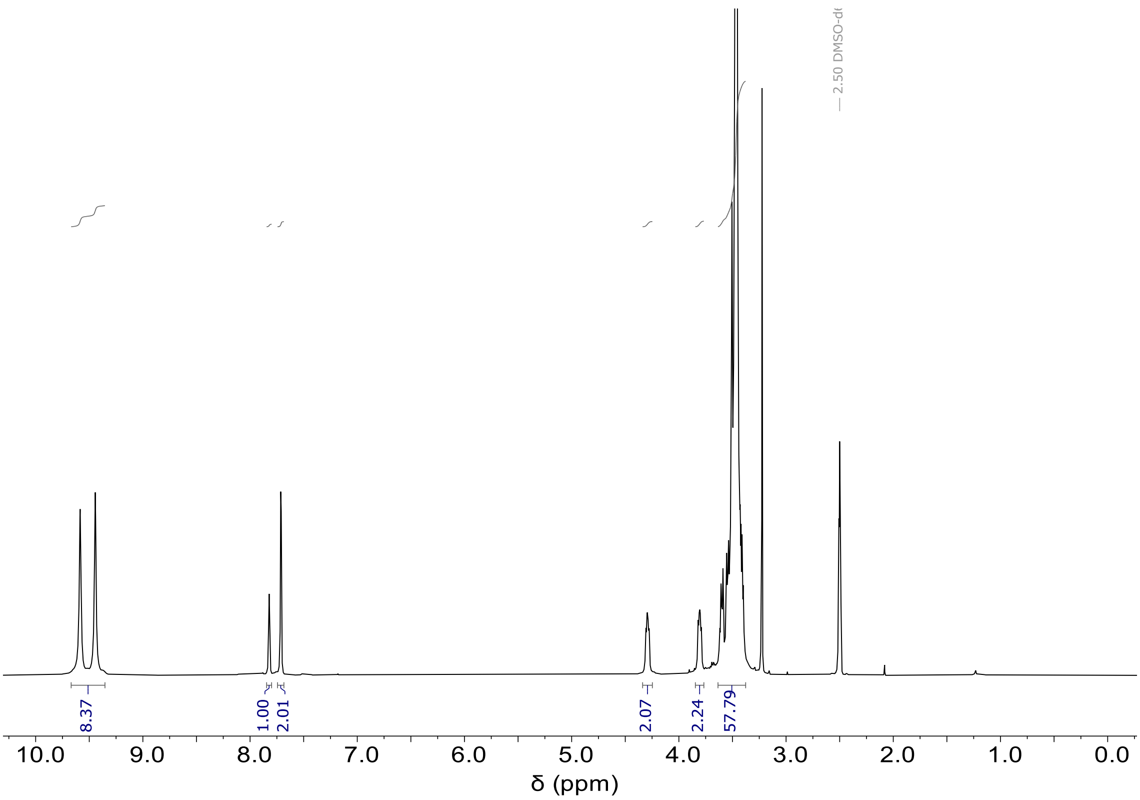

^{1}H NMR spectra of compound **L*A*** in DMSO-D$_6$ (298K, 300 MHz).

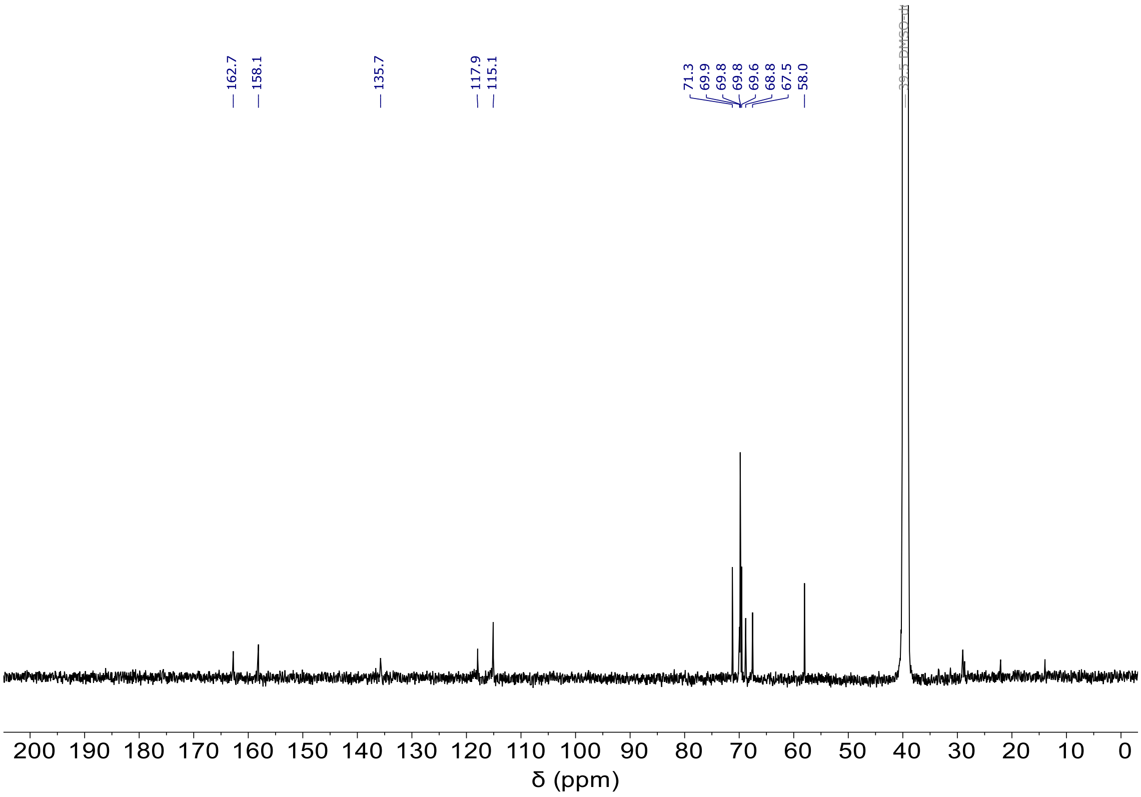

^{13}C NMR spectra of compound **L*A*** in DMSO-D$_6$ (298K, 125 MHz).

S1. ^{1}H NMR Titration Experiments

As shown in ***Figure S1***, the self-assembly of $\mathbf{P_{2H}{}^{O}{}_2{\cdot}L^{O}{}_4}$ (left) and $\mathbf{P_{Zn}{}^{O}{}_2{\cdot}L^{O}{}_4}$ (right) systems was first confirmed through a set of ^{1}H NMR experiments in which the proportion of each **P** and **L** component was varied from 1:0 (purple) to 0:1 (green). At a 1:2 ratio (orange), a single set of proton signals is detected for both assemblies, that differ from those of the individual **P** and **L** components and integrate as expected for a 2+4 complex. The protons involved in the amidinium-carboxylate interaction (ε+γ) are observed around 13-14 ppm. Additionally, the amidinium proton not participating in the interaction (δ) appears at 8.8 ppm, as a broader and solvent-dependent signal. It is also observed that, upon complexation, all proton signals corresponding to **P** shift upfield, whereas those of the **L** component shift downfield. Moreover, the clear multiplicity observed for the phenyl protons of the **P** units located closest to the porphyrin core (*A* and *B* protons, at 8.5-8.0 ppm), is lost in the bound state, which is tentatively attributed to a restricted rotation around the *meso*-bonds. In addition, for $\mathbf{P_{2H}{}^{O}{}_2{\cdot}L^{O}{}_4}$, an extra set of signals (*H*) appears at negative chemical shifts around -3 ppm, corresponding to the NH protons of the pyrrole units positioned within the porphyrin core.

By analyzing intermediate proportions, where there is an excess of **P** (between the 1:0 and 1:2 ratios) or **L** (between the 1:2 and 0:1 ratios), two distinct exchange dynamic regimes between bound (orange) and unbound (purple for **P** and green for **L**) units are identified. For **P**, a slow exchange process is observed on the NMR relaxation time scale. As the amount of **L** increases, the intensity of the bound porphyrin signals (orange) rises while that of the free porphyrin signals (purple) decreases, until they disappear at the 1:2 ratio. In contrast, **L** exhibits fast NMR exchange, as increasing the amount of **P** leads to a gradual shift of the signals, resulting in an averaged state (green). This difference may arise from the fact that, in the assembled state, each **L** forms two binding interactions within the assembly, whereas each **P** unit establishes four interactions, thereby influencing their exchange dynamics.

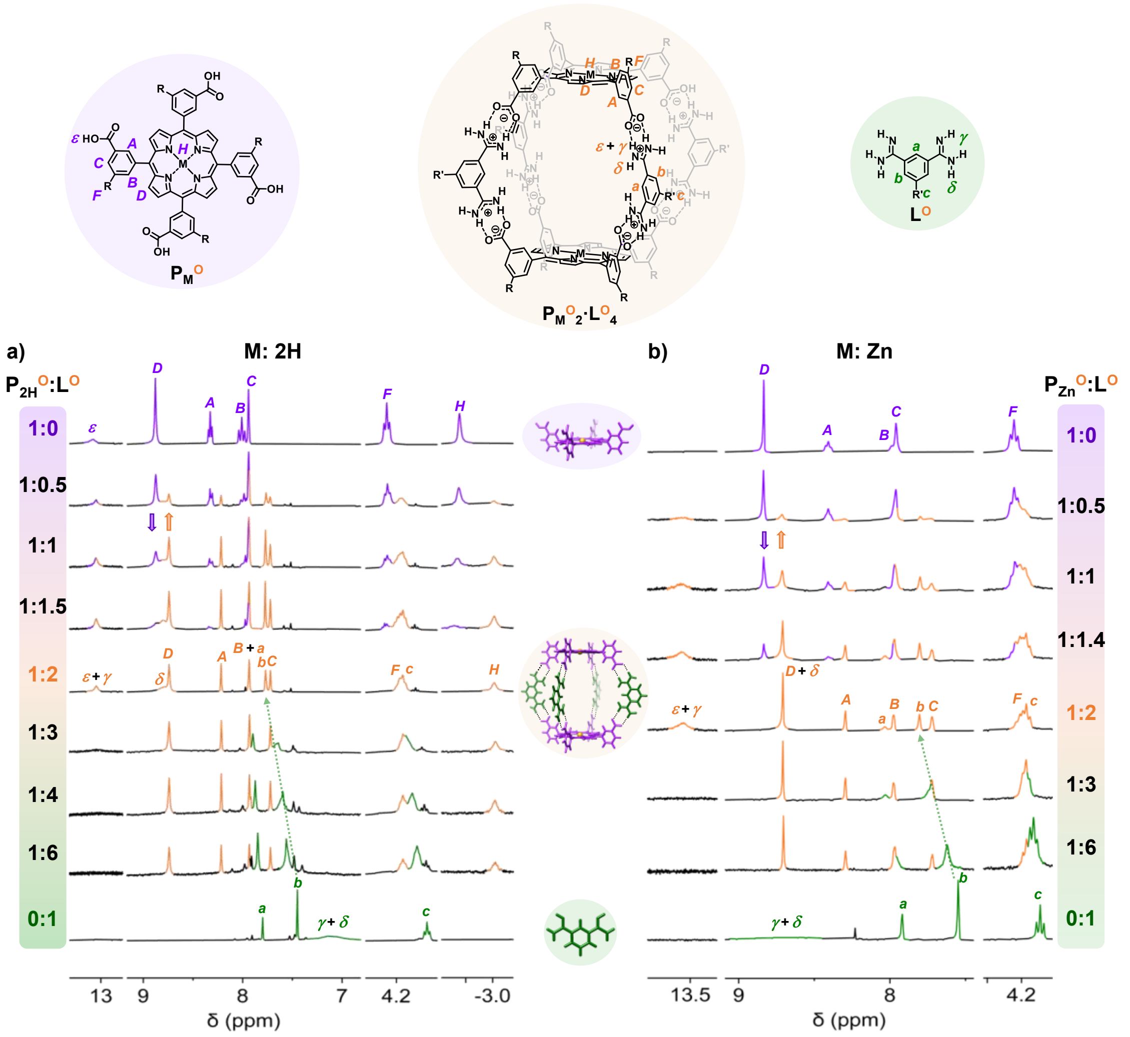

Figure S1. Selected regions of the ¹H NMR spectra in THF-D8/DMSO-D6 **a)** 25:75 and **b)** 75:25 mixtures, obtained by varying the proportion of **a)** $\mathbf{P_{2H}^{O}:L^{O}}$ and **b)** $\mathbf{P_{Zn}^{O}:L^{O}}$. The signals marked in purple and green correspond to the free **P** and **L** subunits, respectively, while those in orange correspond to the self-assembled subunits ($\mathbf{P^{O}_2 \cdot L^{O}_4}$). Proportions were obtained from solutions of **a)** $[\mathbf{P_{2H}^{O}}] = [\mathbf{L^{O}}]$ = 1.8 mM and **b)** $[\mathbf{P_{Zn}^{O}}] = [\mathbf{L^{O}}]$ = 6.9 mM.

S2. Temperature-dependent ^{1}H NMR experiments at different P_{Zn}^{O}:L^{O} ratios

To explore further the dynamics observed at substoichiometric ratios of **P** and **L**, temperature dependent ^{1}H NMR experiments were conducted, attempting to switch from slow to fast NMR exchange for the **P** component, or from fast to slow NMR exchange for the **L** units, respectively. As shown in ***Figure S2*****a**, the slow exchange observed for **P** signals persists upon heating from 298 K to 333 K, although some signal broadening is observed, for instance, in the amidinium-carboxylate interaction signals ($\varepsilon + \gamma$), as well as in the aromatic signals around 7.7-9.0 ppm. Likewise, the fast exchange exhibited by **L** remains when a mixture containing of $\mathbf{P_{Zn}^{O}}$ and free $\mathbf{L^{O}}$ was cooled from 298 K to 263 K (***Figure S2*****c**). Furthermore, signal sharpening is observed due to the temperature decrease, particularly in the characteristic $\varepsilon + \gamma$ signals corresponding to the supramolecular interaction and, for example, in the *a* proton of the $\mathbf{L^{O}}$ unit at around 8.0 ppm.

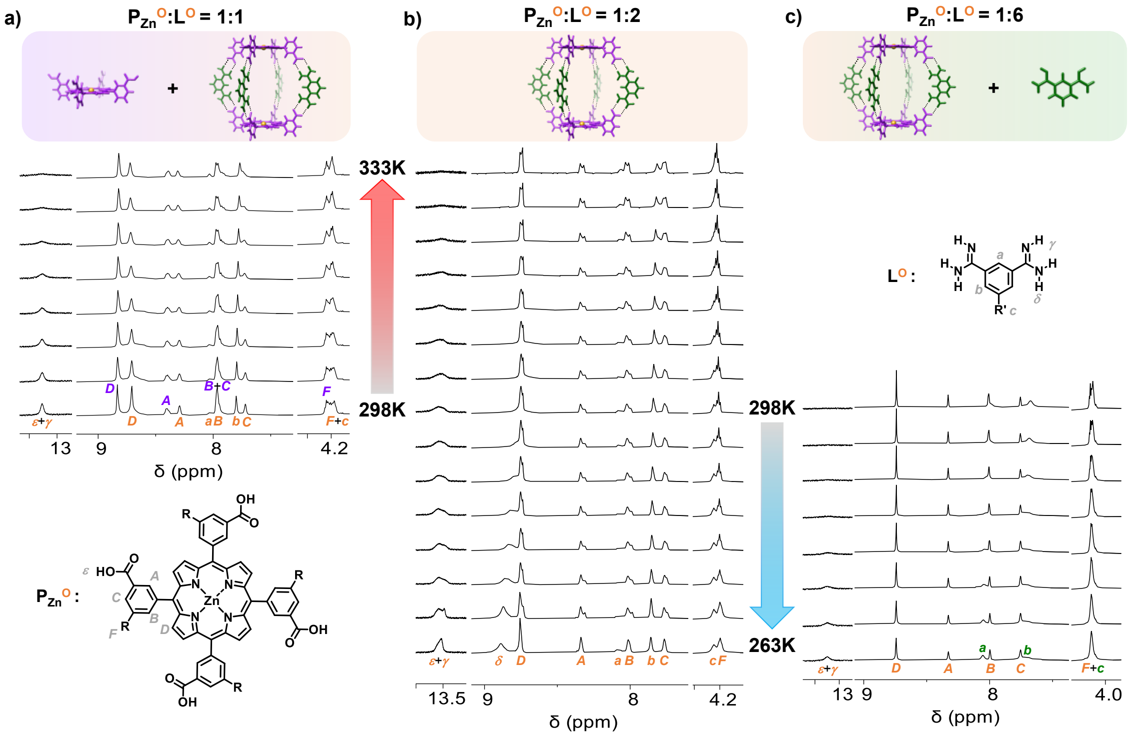

Figure S2. ^{1}H NMR spectra of $\mathbf{P_{Zn}^{O}}$ + $\mathbf{L^{O}}$ mixtures at ratios: **a**) 1:1 (excess **P**), **b**) 1:2 ($\mathbf{P_{Zn}^{O}{}_2 \cdot L^{O}{}_4}$), and **c**) 1:6 (excess **L**), in THF-D_8/DMSO-D_6 (75:25) at different temperatures. The mixtures were prepared from stock solutions at [$\mathbf{P_{Zn}^{O}}$] = [$\mathbf{L^{O}}$] = 6.9 mM concentration. The signal assignment applied in ***Figure S1*** was maintained.

Additionally, the stoichiometric 1:2 $\mathbf{P_{Zn}^{O}}$:$\mathbf{L^{O}}$ mixture was cooled from 333 K to 263 K covering the whole range explored for both substoichiometric mixtures, as shown ***Figure S2*****b.** Notably, at high temperatures, the $\varepsilon + \gamma$ signals at 13.5 ppm exhibit significant broadening, but no dissociation was observed, highlighting the robustness of assembly $\mathbf{P_{Zn}^{O}{}_2 \cdot L^{O}{}_4}$. At lower temperatures, the protons corresponding to the amidinium moiety (ε, γ and δ) are sharpened, probably due to the freezing of both the NH_2 and carboxylic group rotation

S3. ^{1}H DOSY NMR experiments at different P_{Zn}^{O}:L^{O} ratios

To further confirm the formation of $\mathbf{P^{O}_2{\cdot}L^{O}_4}$ by an independent method, Diffusion-Ordered Spectroscopy (DOSY) ^{1}H NMR experiments were performed. Spectra were recorded for the individual components, $\mathbf{L^{O}}$ and $\mathbf{P_{Zn}^{O}}$, as well as for their stoichiometric mixture used to generate the assembly $\mathbf{P_{Zn}^{O}{}_2{\cdot}L^{O}_4}$ (1:2). As shown in ***Figure S3***, all signals in the $\mathbf{P_{Zn}^{O}{}_2{\cdot}L^{O}_4}$ sample exhibit the same diffusion behaviour, indicating the presence of a single supramolecular complex. Moreover, when compared to the individual precursors, the diffusion coefficients ($\mathbf{D_i}$) are clearly distinct, with $\mathbf{P_{Zn}^{O}{}_2{\cdot}L^{O}_4}$ displaying the lowest value, consistent with its larger size (see ***Table S1***).

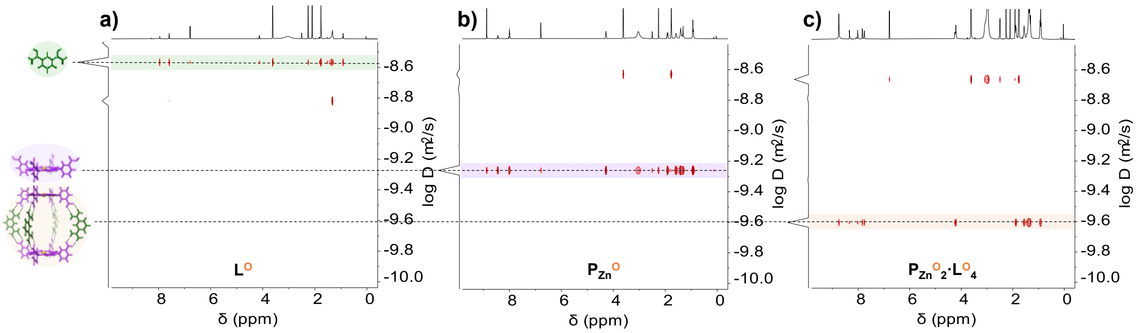

Figure S3. ^{1}H DOSY NMR spectra recorded at 298 K in THF-D$_8$/DMSO-D$_6$ (75:25) of: **a)** $\mathbf{L^{O}}$ (6.9 mM, Δ (D20) = 100 ms, $\delta/2$ (P30) = 1100 μs), **b)** $\mathbf{P_{Zn}^{O}}$ (6.9 mM, Δ (D20) = 150 ms, $\delta/2$ (P30) = 1100 μs), **c)** $\mathbf{P_{Zn}^{O}{}_2{\cdot}L^{O}_4}$ (2.3 mM, Δ (D$_2$0) = 200 ms, $\delta/2$ (P30) = 1300 μs), using mesitylene as an internal reference.

Compound	Relaxation curves	Peak (ppm)	D_i (cm^2/s)	$\bar{D}_i$ (cm^2/s)
$\mathbf{L^{O}}$	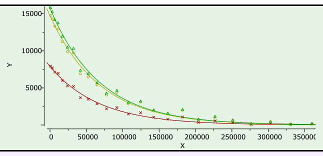	7.97<br>7.62<br>4.16	$1.482{\cdot}10^{-5}$<br>$1.470{\cdot}10^{-5}$<br>$1.477{\cdot}10^{-5}$	$\mathbf{1.476{\cdot}10^{-5}}$<br>$\pm\ 6{\cdot}10^{-8}$
$\mathbf{P_{Zn}^{O}}$	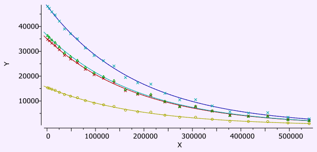	8.76<br>8.35<br>7.89<br>4.18	$5.28{\cdot}10^{-6}$<br>$5.22{\cdot}10^{-6}$<br>$5.30{\cdot}10^{-6}$<br>$5.31{\cdot}10^{-6}$	$\mathbf{5.28{\cdot}10^{-6}}$<br>$\pm\ 4{\cdot}10^{-8}$
$\mathbf{P_{Zn}^{O}{}_2{\cdot}L^{O}_4}$	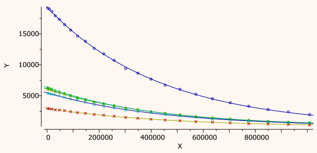	8.23<br>7.92<br>7.74<br>7.66<br>4.17	$2.22{\cdot}10^{-6}$<br>$2.26{\cdot}10^{-6}$<br>$2.38{\cdot}10^{-6}$<br>$2.26{\cdot}10^{-6}$<br>$2.29{\cdot}10^{-6}$	$\mathbf{2.28{\cdot}10^{-6}}$<br>$\pm\ 6{\cdot}10^{-8}$

Table S1. Experimental diffusion coefficients (D_i) obtained from the ^{1}H DOSY NMR spectra at 298 K in THF-D$_8$/DMSO-D$_6$ (75:25) (***Figure S3***).

From the diffusion coefficient (D_i) obtained via the ^{1}H DOSY NMR experiment (***Table S1***), together with the Boltzmann constant (k_B), the experimental temperature (T), and the solvent viscosity (η), the hydrodynamic radius of $\mathbf{P_{Zn}^{O}{}_2{\cdot}L^{O}_4}$ can be estimated using the Stokes-Einstein

equation[8] for spherical objects (***Figure S4a***). This calculation yields a radius of 1.13 nm, which agrees reasonably well with the radius (1.4 nm) obtained from the computational model (***Figure S4b***), considering that $\mathbf{P_{Zn}{}^{O}{}_{2}\cdot L^{O}{}_{4}}$ is not a perfect sphere and features a solvent-accessible internal cavity.

a)

$$r_i = \frac{k_B \cdot T}{6 \cdot \pi \cdot \eta \cdot D_i} \approx \frac{1.38 \cdot 10^{-23}\left(\frac{J}{K}\right) \cdot 298\ (K)}{6 \cdot \pi \cdot 8.44 \cdot 10^{-4}(Pa \cdot s) \cdot 2.28 \cdot 10^{-10}\left(\frac{m^2}{s}\right)} \approx 1.13\ nm$$

b)

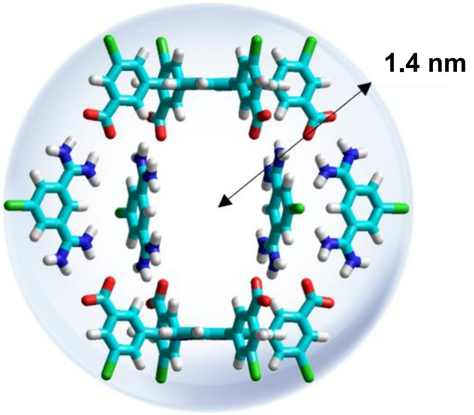

Figure S4. **a**) Hydrodynamic radius calculation of $\mathbf{P_{Zn}{}^{O}{}_{2}\cdot L^{O}{}_{4}}$ via the Stokes-Einstein equation. The viscosity used corresponds to the weighted average viscosity of the non-deuterated solvent mixture (THF/DMSO 75:25) at 298K. **b**) Computational model of the $\mathbf{P_{Zn}{}^{O}{}_{2}\cdot L^{O}{}_{4}}$ system without solubilizing chains. The resulting structure exhibits an approximate radius of 1.4 nm.

The slow exchanges behaviour observed in the ^{1}H NMR spectra shown in ***Figure S1***, when an excess of $\mathbf{P_{Zn}^{O}}$ was present in the sample, enabled the differentiation of $\mathbf{P_{Zn}^{O}}_2\mathbf{\cdot L^{O}}_4$ and $\mathbf{P_{Zn}^{O}}$ in the same sample. Indeed, the ^{1}H DOSY NMR experiment of a 1:1 mixture of $\mathbf{P_{Zn}^{O}}$ and $\mathbf{L^{O}}$ (***Figure S5***) confirmed that the free and bound $\mathbf{P_{Zn}^{O}}$ species diffuse independently (***Table S2***).

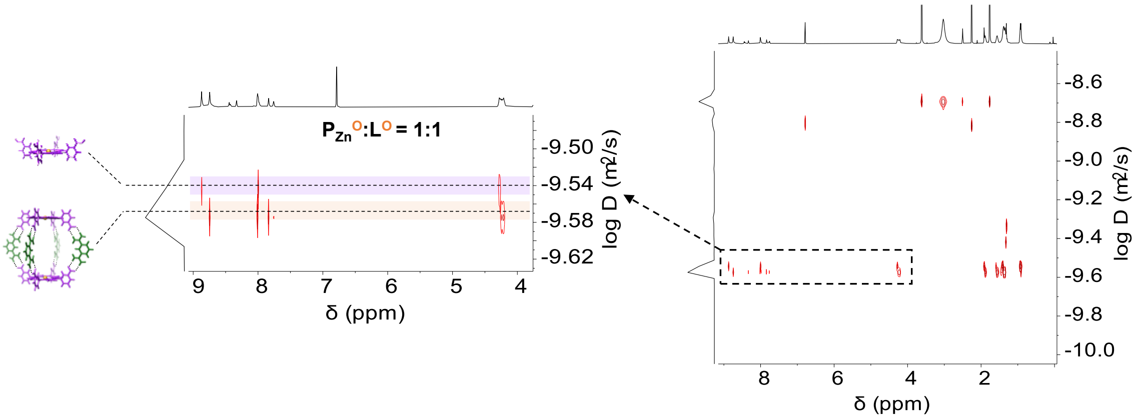

Figure S5. ^{1}H DOSY NMR spectra recorded at 298 K in THF-D_8/DMSO-D_6 (75:25) of $\mathbf{P_{Zn}^{O}}$:$\mathbf{L^{O}}$ mixture at ratio 1:1, from stock solutions at [$\mathbf{P_{Zn}^{O}}$] = [$\mathbf{L^{O}}$] = 6.9 mM concentration, Δ (D20) = 200 ms, $\delta/2$ (P30) = 1300 μs, using mesitylene as an internal reference.

Compound	Relaxation curves	Peak (ppm)	D_i (cm^2/s)	$\overline{D}_i$ (cm^2/s)
$\mathbf{P_{Zn}^{O}}$	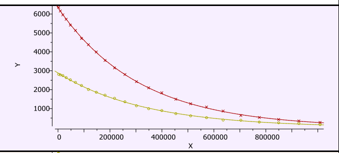	8.76<br>8.35	$3.16\cdot10^{-6}$<br>$2.96\cdot10^{-6}$	$\mathbf{3.07\cdot10^{-6}}$<br>$\pm 1.4\cdot10^{-7}$
$\mathbf{P_{Zn}^{O}}_2\mathbf{\cdot L^{O}}_4$	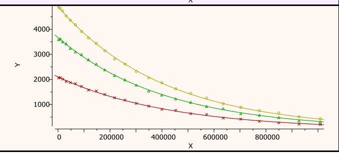	8.23<br>7.74<br>7.66	$2.31\cdot10^{-6}$<br>$2.44\cdot10^{-6}$<br>$2.41\cdot10^{-6}$	$\mathbf{2.39\cdot10^{-6}}$<br>$\pm 7\cdot10^{-8}$

Table S2. Experimental values of diffusion coefficients (D_i) obtained from the ^{1}H DOSY NMR spectra of $\mathbf{P_{Zn}^{O}}$:$\mathbf{L^{O}}$ mixture at ratio 1:1 in THF-D_8/DMSO-D_6 (75:25) at 298 K (***Figure S5***).

S4. High-resolution mass spectrometry analysis of $\mathbf{P_{2H}^{O}{}_{2}\cdot L^{O}{}_{4}}$

Additionally, the supramolecular system $\mathbf{P_{2H}^{O}{}_{2}\cdot L^{O}{}_{4}}$ was characterized by high-resolution electrospray ionization mass spectrometry (HR-ESI-MS) in positive mode at low temperature (273K) to enhance the detection of multiply charged ions. The $\mathbf{P_{2H}^{O}{}_{2}\cdot L^{O}{}_{4}}$ complex, with a calculated mass of 3766.2168 for $C_{224}H_{292}N_{24}O_{28}$ $[M]^{+}$, was detected in the HR-ESI mass spectrum. As shown in ***Figure S6*a**, a peak at *m/z* 1885.1167 with a 2+ charge was identified, matching the theoretical isotopic distribution (***Figure S6*b**) and corresponding to half the $[M]^{+}$ mass due to its doubly charged state. Furthermore, the spectrum in ***Figure S6*a** also displays peaks resulting from the sequential loss of **L** units, related to $\mathbf{P^{O}{}_{2}\cdot L^{O}{}_{3}}$, $\mathbf{P^{O}{}_{2}\cdot L^{O}{}_{2}}$, and $\mathbf{P^{O}{}_{2}\cdot L^{O}{}_{1}}$ observed at *m/z* 1711.9833 (2+), 1538.8451 (2+), and 2729.4068 (+), respectively.

a)

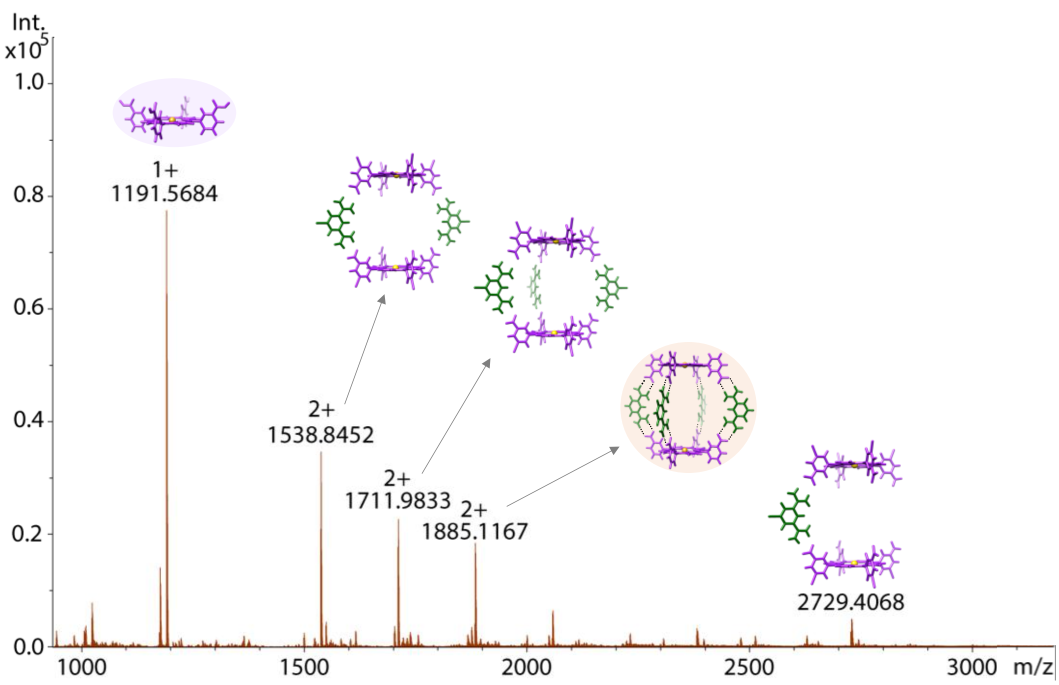

b)

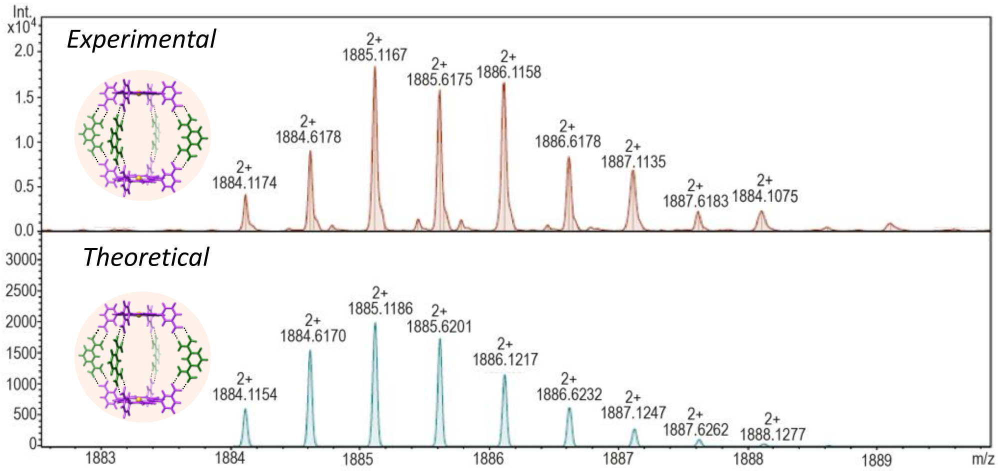

Figure S6. **a**) HRMS (ESI +) spectrum of $\mathbf{P_{2H}^{O}{}_{2}\cdot L^{O}{}_{4}}$ recorded at 273 K in a THF/DMSO 10:1 mixture. **b**) Comparison between the experimental (top) and theoretical (bottom) isotopic distribution of the parent ion $[M]^{2+}$.

S5. ^{1}H NMR experiments at different solvent compositions and concentrations

The stability of the **$P_2\cdot L_4$** complex was evaluated in different THF-D_8/DMSO-D_6 mixtures. As shown in ***Figure S7***, the **$P_{Zn}{}^{O}{}_2\cdot L^{O}{}_4$** assembly remains stable in THF-rich environments; however, its solubility decreases when the THF content exceeds 90%. Similarly, ***Figure S8*** illustrates that **$P_{2H}{}^{O}{}_2\cdot L^{O}{}_4$** is well-tolerated in DMSO-rich media, though precipitation begins at DMSO concentrations above 90%. In both cases, no evidence of complex dissociation was observed, with only minor chemical shift variations. Note that outside the ranges shown in ***Figures S7*** and ***S8***, a purple solid crashed out when both components are combined in stoichiometric amounts. This phenomenon could be due to either the low solubility of the assembly or the formation of higher-order assemblies under these conditions.

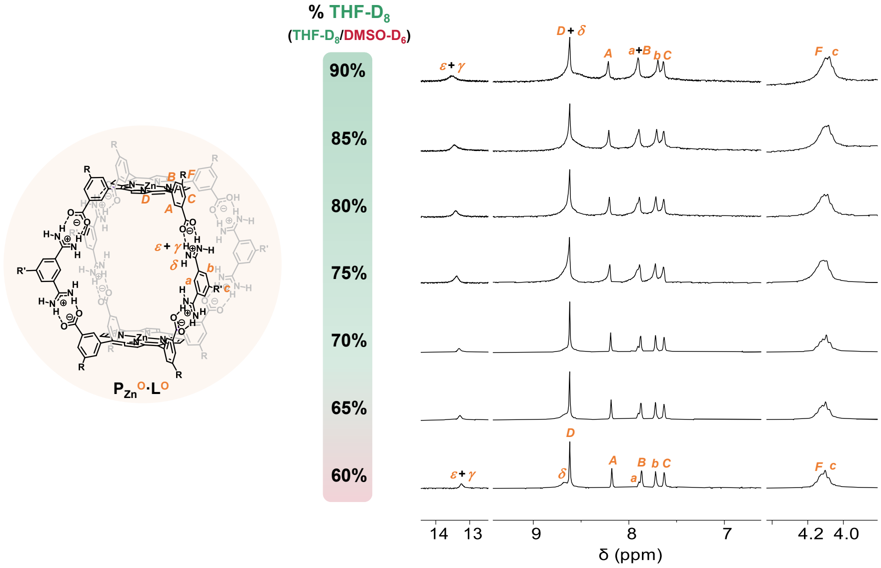

Figure S7. Selected regions of the ^{1}H NMR spectra of **$P_{Zn}{}^{O}{}_2\cdot L^{O}{}_4$** (1mM) recorded in THF-D_8/DMSO-D_6 mixtures, ranging from 90:10 (top) to 60:40 (bottom) at 298 K. For ^{1}H signal assignment see ***Figure S1***.

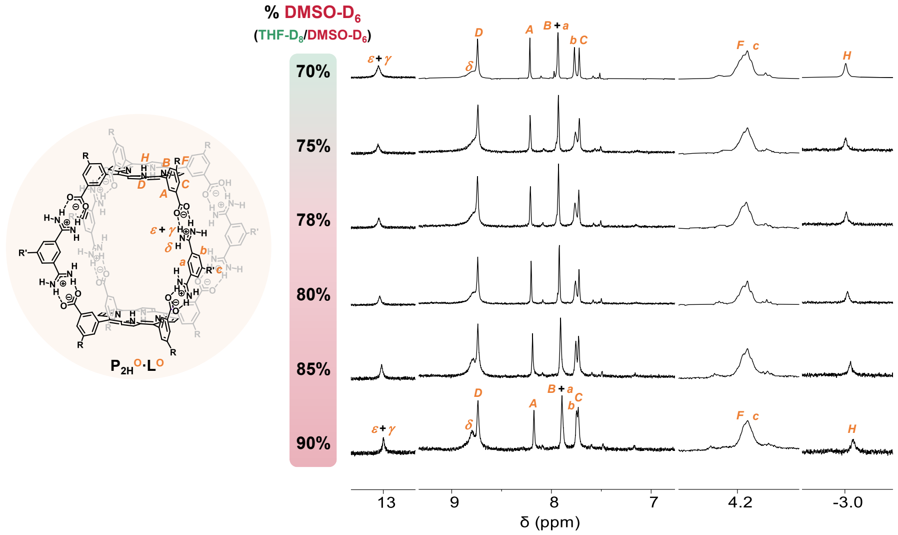

Figure S8. Selected regions of the ^{1}H NMR spectra of **$P_{2H}{}^{O}{}_2\cdot L^{O}{}_4$** (0.1mM) recorded in THF-D_8/DMSO-D_6 mixtures, ranging from 30:70 (top) to 10:90 (bottom) at 298 K. For ^{1}H signal assignment see ***Figure S1***.

To further evaluate the stability of the assembly, a series of ^1H NMR spectra were recorded at various concentrations (***Figure S9***), ranging from 5·mM to 10·µM of $\mathbf{P_{2H}{}^{O}{}_2{\cdot}L^{O}{}_4}$, the latter concentration corresponding to the experimental detection limit of the NMR equipment. The signals corresponding to the bound subunits within the supramolecular complex remained visible across the entire concentration range, although the **L** resonances exhibited slight shifts and/or broadening. However, these shifts are quite small (ca. 0.1-0.05 ppm), certainly smaller than the 0.3-0.5 shifts expected for full dissociation, as demonstrated by the comparison with the reference spectra at the bottom of ***Figure S9*** at the same concentration. This indicates that the [2+4] assembly is stable across a wide concentration range. Only the exchangeable protons are seen to sharpen as the concentration is decreased. Please note that in the ^1H NMR spectrum recorded at 10 µM, new peaks appeared around 8.5 ppm, corresponding to the stabilizing agents from the deuterated solvents as a result of the high dilution.

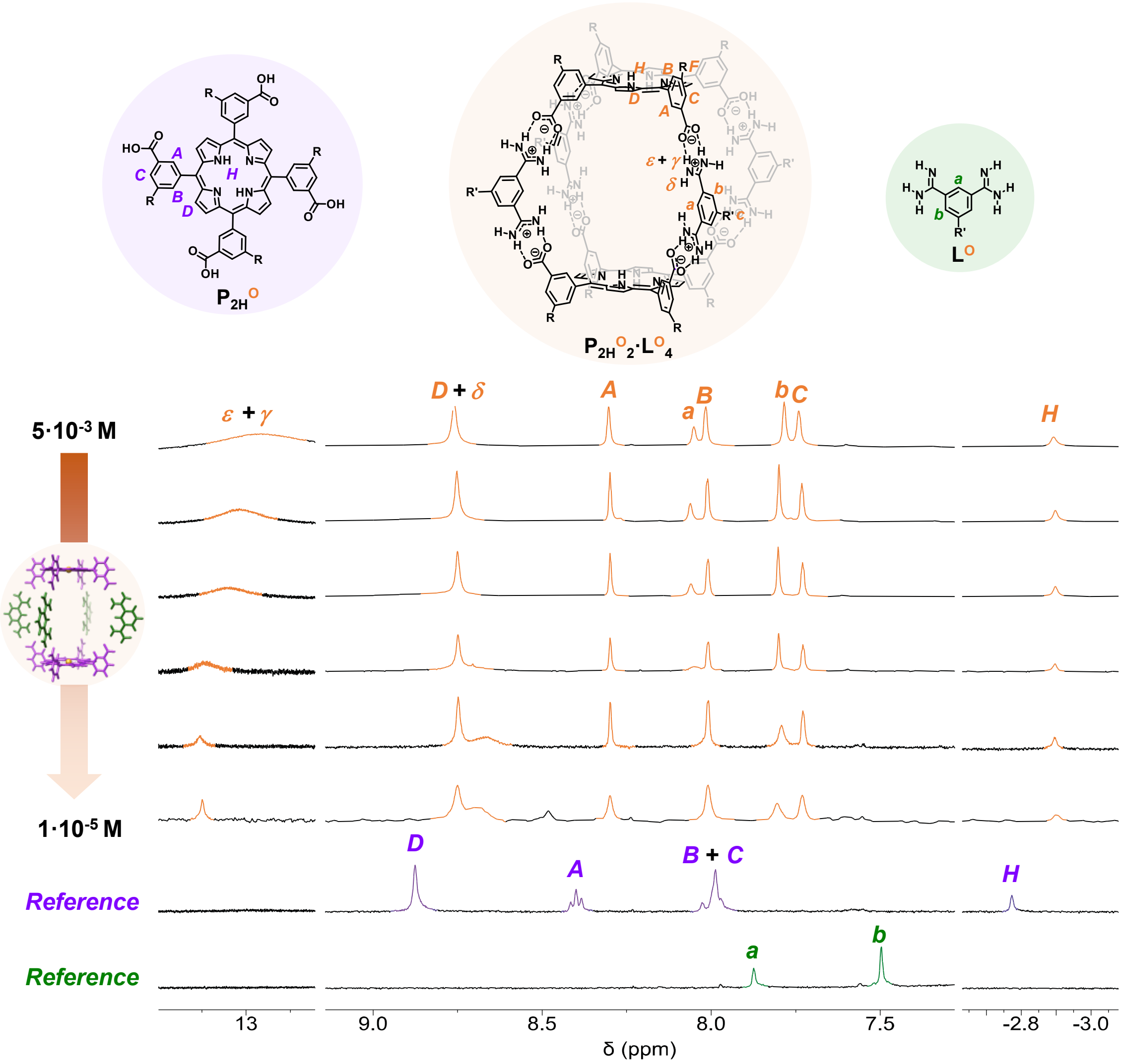

Figure S9. ^1H NMR spectra of $\mathbf{P_{2H}{}^{O}{}_2{\cdot}L^{O}{}_4}$ recorded from 5·mM (top) to 10·μM (bottom) in THF-D8/DMSO-D6 (75:25) at 298 K. The ^1H NMR spectra of $\mathbf{P_{2H}{}^{O}}$ and $\mathbf{L^{O}}$ recorder under the same diluted conditions was included as reference at the bottom.

S6. Absorption and emission experiments

In addition to NMR experiments, UV-Vis and fluorescence measurements were carried out to study the system using more sensitive techniques at lower concentrations (***Figure S10***). In both cases, the linker showed no detectable signal, and no significant differences were observed between the spectra of $\mathbf{P_{Zn}^{O}}$ and the $\mathbf{P_{Zn}^{O}{}_{2}\cdot L^{O}{}_{4}}$ system (absorption and emission spectra basically overlap). Considering that at a concentration of 10^{-5} M the NMR spectrum clearly displays the characteristic signals of the assembly (see ***Figure S9***), while the absorption spectrum at the same concentration does not, these results indicate that ensemble formation does not alter significantly the porphyrin photophysical properties.

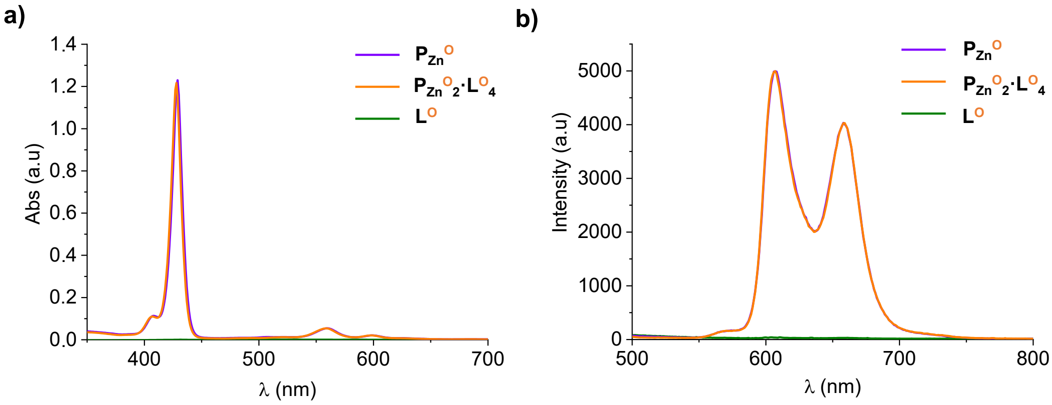

Figure S10. Comparison of **a)** UV-Vis absorption (1 cm cell) and **b)** fluorescence emission (λ_{exc} = 429 nm) spectra of $\mathbf{P_{Zn}^{O}}$ in purple ([$\mathbf{P_{Zn}^{O}}$] = $3\cdot10^{-6}$ M), $\mathbf{L^{O}}$ in green ([$\mathbf{L^{O}}$] = $6\cdot10^{-6}$ M), and $\mathbf{P_{Zn}^{O}{}_{2}\cdot L^{O}{}_{4}}$ in orange ([$\mathbf{P_{Zn}^{O}{}_{2}\cdot L^{O}{}_{4}}$] = $1.5\cdot10^{-6}$ M), dissolved in a THF/DMSO mixture (75:25).

S7. Calculation of thermodynamic parameters: K_{a2}, K_C, K_T and *EM*.

Due to the high stability exhibited by the $P^{O}_2 \cdot L^{O}_4$ system in both temperature- and concentration-dependent experiments (see sections S2 and S5), and in order to determine the total association constant (K_T) of the $P_{2H}{}^{O}{}_2 \cdot L^{O}_4$ complex, as will be discussed below, a competition experiment was designed using benzoic acid (**B**) as competitor for the $P_2 \cdot L_4$ complex, as shown in ***Figure S11***.

(1) 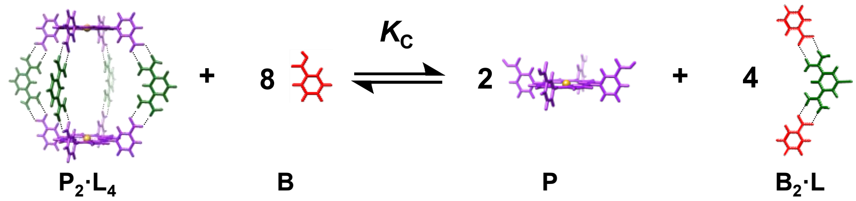

$$K_C = \frac{[P]^2 \cdot [B_2 \cdot L]^4}{[P_2 \cdot L_4] \cdot [B]^8}$$

Figure S11. Scheme of a competition experiment in which benzoic acid (**B**) promotes the dissociation of $P_2 \cdot L_4$, forming free **P** and the $B_2 \cdot L$ complex. The equilibrium constant of this competition is represented by K_C.

As illustrated in ***Figure S12***, large amounts of benzoic acid (**B**) were required to observe significant dissociation of the $\mathbf{P_{2H}{}^{O}{}_2 \cdot L^{O}{}_4}$ assembly in the ^{1}H NMR spectra (30-100 equivalents).

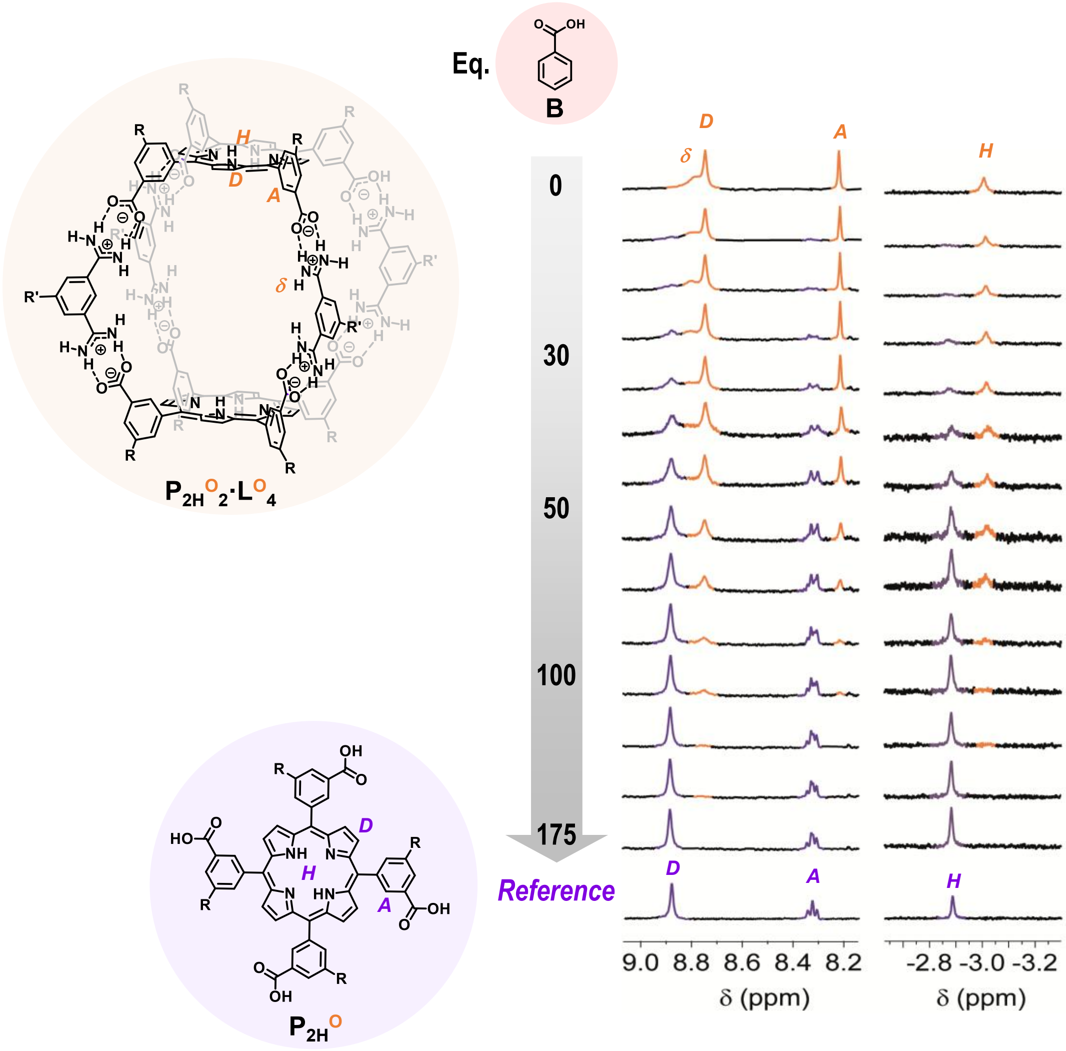

Figure S12. Competition experiment of $\mathbf{P_2 \cdot L_4}$ against benzoic acid monitored by ^{1}H NMR performed at a constant concentration of $\mathbf{P_{2H}{}^{O}{}_2 \cdot L^{O}{}_4}$ ($1 \cdot 10^{-4}$ M) in THF-D_8/DMSO-D_6 mixture (25:75) at 298 K. The ^{1}H NMR spectrum of $\mathbf{P_{2H}{}^{O}}$ recorder under the same conditions was included as reference at the bottom.

Upon formation of the $\mathbf{B_2 \cdot L}$ complex, free **P** remains. Since **P** undergoes slow exchange between the bound and free states, its signal can be integrated (*A* and *H* protons), allowing for the quantification of all species present at equilibrium. This enables the calculation of the competition constant as K_C = 14 ± 4 M^{-3}. Complete dissociation of the $\mathbf{P_{2H}{}^{O}{}_2 \cdot L^{O}{}_4}$ complex requires more than 170 equivalents of **B**, highlighting the high cooperativity of this multicomponent system.

On the other hand, we determined the association constant of the **B₂·L** complex ($K_{a2} = K_1 \cdot K_2$; see ***Figure S13***) formed during the competition experiment (see ***Figure S11***), since it is required for the calculation of K_T, as it will be discussed later.

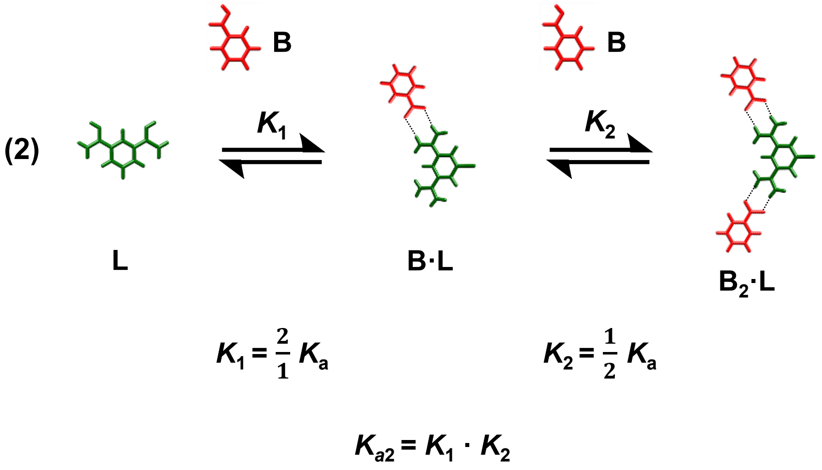

Figure S13. Scheme for the determination of K_{a2} and K_a from a titration experiment, in which benzoic acid (**B**) is associated with **L** in two successive steps to form the **B₂·L** complex.

Hence, a titration experiment between **L**^O and **B** was designed and executed (***Figure S14***, left), revealing a fast exchange between all the species.

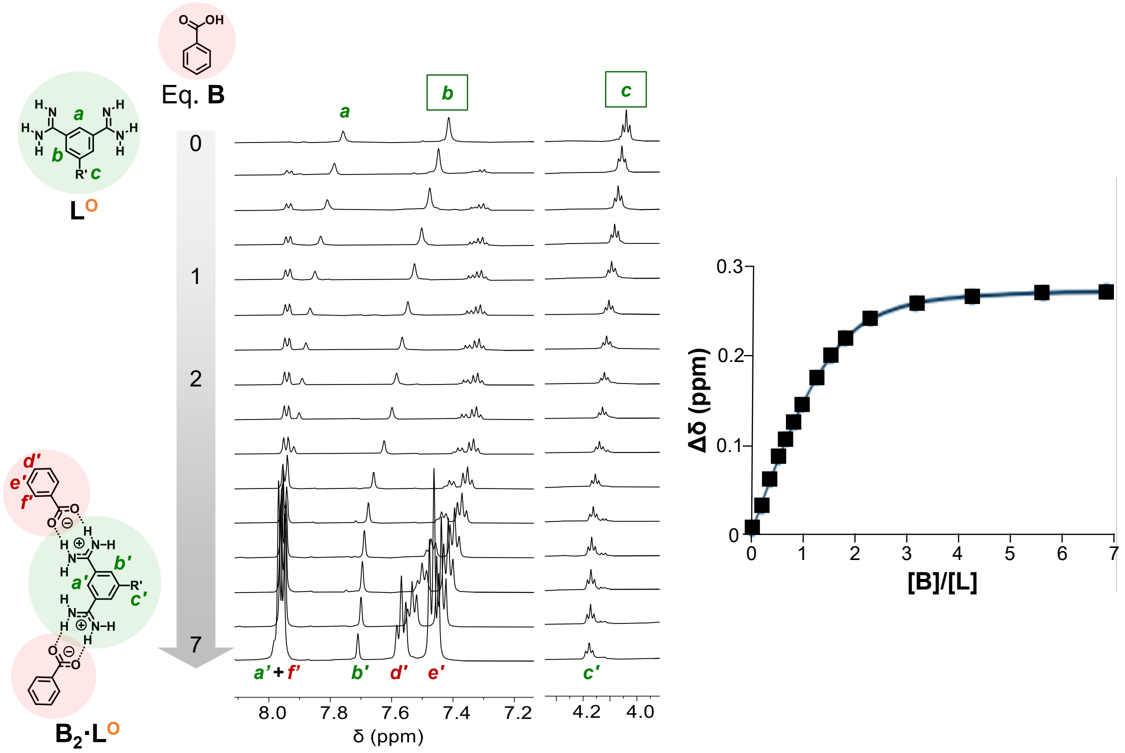

Figure S14. Titration experiment of **L**^O with increasing amounts of benzoic acid (**B**) monitored by ^{1}H NMR and performed at a constant concentration of **L**^O ($7.4 \cdot 10^{-4}$ M) in THF-D₈/DMSO-D₆ (25:75) at 298 K. The chemical shift of the framed signals (*b* and *c*), as a function of B equivalents, was used to determine the association constants K_1 and K_2 by fitting the data to a 1:2 (**L**:**B**) binding model.

The chemical shift changes of signals *b* and *c* (***Figure S14***) were fitted to a 1:2 model, yielding association constants for the first and second binding events of $\boldsymbol{K_1}$ = 1.55·10^4 M^{-1} and $\boldsymbol{K_2}$ = 4.25·10^3 M^{-1}. This means that the the association constant of the **B₂·L**^{O} complex is $\boldsymbol{K_{a2}} = \boldsymbol{K_1}\cdot\boldsymbol{K_2}$ = (6.6 ± 1.0)·10^7 M^{-2}. Based on the value of $\boldsymbol{K_1}$ and considering statical factors (see ***Figure S13***), a reference binding constant ($\boldsymbol{K_a}$) for the amidinium-carboxylate interaction can be calculated as $\boldsymbol{K_a}$ = 7.75·10^3 M^{-1}.

Once $\boldsymbol{K_C}$ and $\boldsymbol{K_{a2}}$ were determined, $\boldsymbol{K_T}$ could be calculated, as shown in ***Figure S15***, as $\boldsymbol{K_T}$ = ($\boldsymbol{K_{a2}}$)^4/ $\boldsymbol{K_C}$, affording a value of $\boldsymbol{K_T}$ = (1.6± 0.5) 10^{30} M^{-5}.

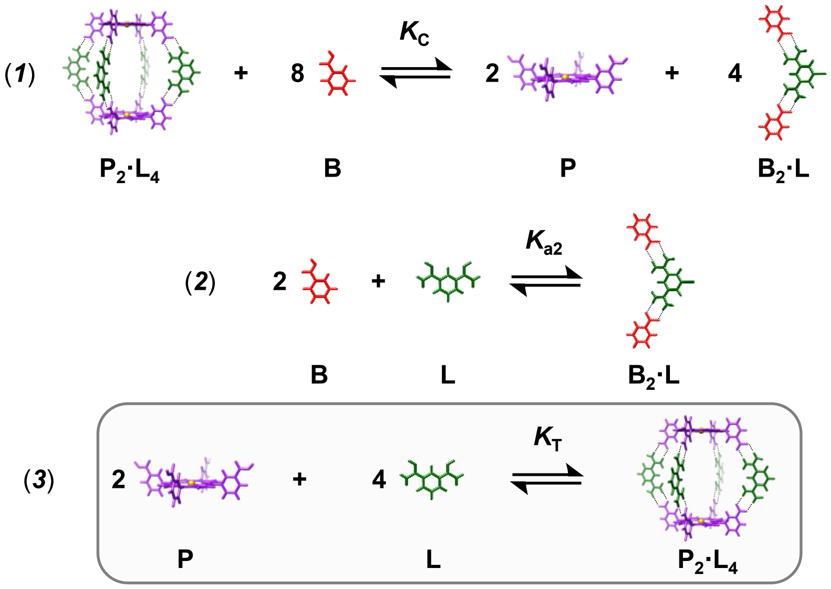

(**3**) = 4 · (**2**) - (**1**) $\qquad K_T = \frac{(K_{a2})^4}{K_C}$

Figure S15. Scheme illustrating the relationship between the different equilibria and the calculation of $\boldsymbol{K_T}$ from $\boldsymbol{K_{a2}}$ and $\boldsymbol{K_C}$.

Figures S16 and ***S17*** represent two plausible different self-assembly pathways along the formation of the $\mathbf{P_2{\cdot}L_4}$ complex. In each pathway, each binding event can be associated to the binding constant of a single amidinium-carboxylate interaction ($\boldsymbol{K_a}$) that is corrected by the corresponding statistical factor. This reference binding constant was previously estimated as half the value of the first association constant between a carboxylic acid compound and a ditopic amidine (see above). The multiplication of the statistical factors arising from the eight successive binding events involved in the formation of the ensemble results in a global statistical factor of 32. In addition, along the formation of the $\mathbf{P_2{\cdot}L_4}$ complex, three intramolecular cycles are generated through amidinium-carboxylate interactions. Each of these cyclization events ($\boldsymbol{EM_1}$, $\boldsymbol{EM_2}$ and $\boldsymbol{EM_3}$ in ***Figure S16*** or $\boldsymbol{EM'_1}$, $\boldsymbol{EM'_2}$ and $\boldsymbol{EM'_3}$ in ***Figure S17***) is associated with an effective molarity (*EM*) value, reflecting the intramolecular nature and cooperativity of the assembly process. We expect the *EM* values to increase as the whole assembly is being formed, meaning that $\boldsymbol{EM_1} < \boldsymbol{EM_2} < \boldsymbol{EM_3}$, and $\boldsymbol{EM'_1} < \boldsymbol{EM'_2} < \boldsymbol{EM'_3}$.

Consequently, the total association constant (***K*$_T$**) can be related to the statistical factors, the *EM* values and the binding constant of a single amidinium-carboxylate interaction (***K*$_a$**) as: ***K*$_T$** = 32·$\boldsymbol{K_a}^8 \cdot \overline{\boldsymbol{EM}}^3$.[9] Based on this model, $\overline{\boldsymbol{EM}}$, the mean *EM* value of the 3 cooperative cyclization process leading to **$P_{2H}{}^{O}{}_2$·$L^{O}{}_4$**, was estimated to be $\overline{\boldsymbol{EM}}$ = 0.157 M.

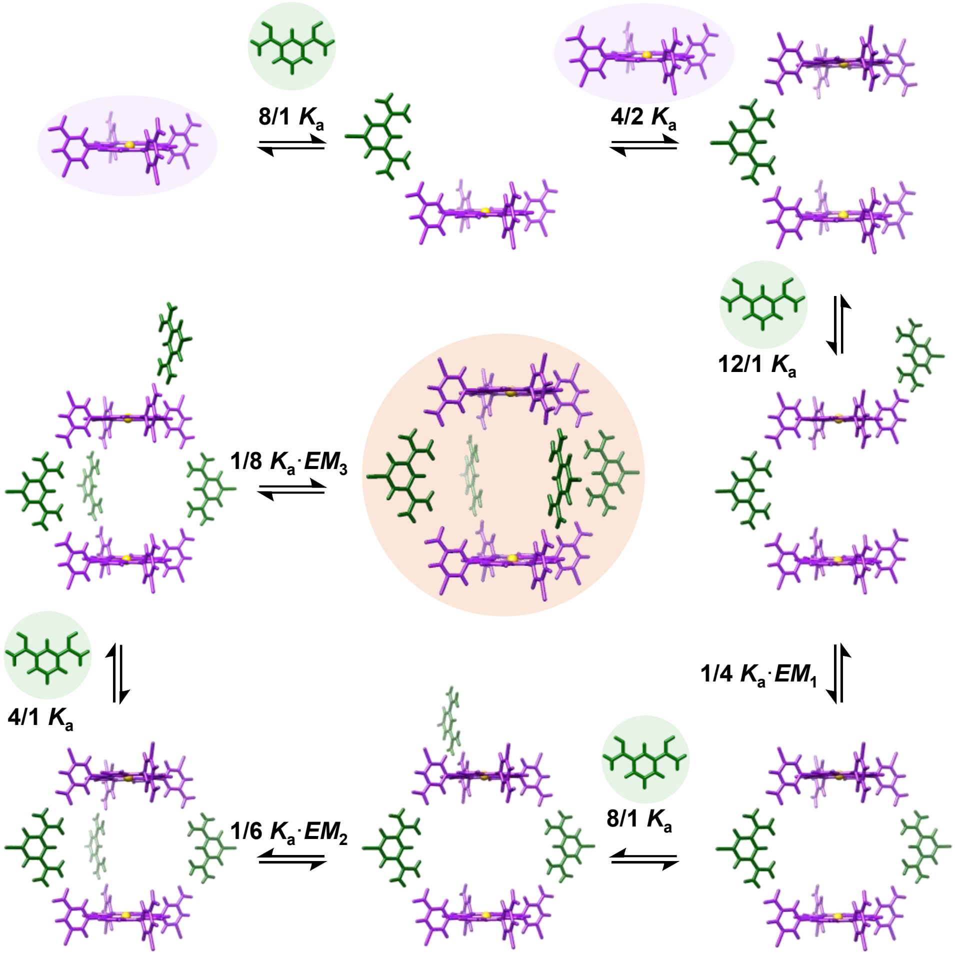

$$\boldsymbol{K_T} = \frac{8\cdot4\cdot12\cdot1\cdot8\cdot1\cdot4\cdot1}{1\cdot2\cdot1\cdot4\cdot1\cdot6\cdot1\cdot8}\, K_a{}^{8}\cdot EM_1\cdot EM_2\cdot EM_3 = \mathbf{32}\cdot\boldsymbol{K_a}^{8}\cdot\overline{\boldsymbol{EM}}^{3}$$

***Figure S16**.* Schematic representation of the different equilibria involved in the stepwise formation of the **P_2·L_4** supramolecular assembly. The global association constant K_T was related to the reference binding constant between carboxylate and amidinium functions (K_a) and the derived statistical correction factor (32). Note that the effective molarity *EM* shown in the final equation is the average value of the 3 different *EMs* (EM_1-EM_3) present in these equilibria.

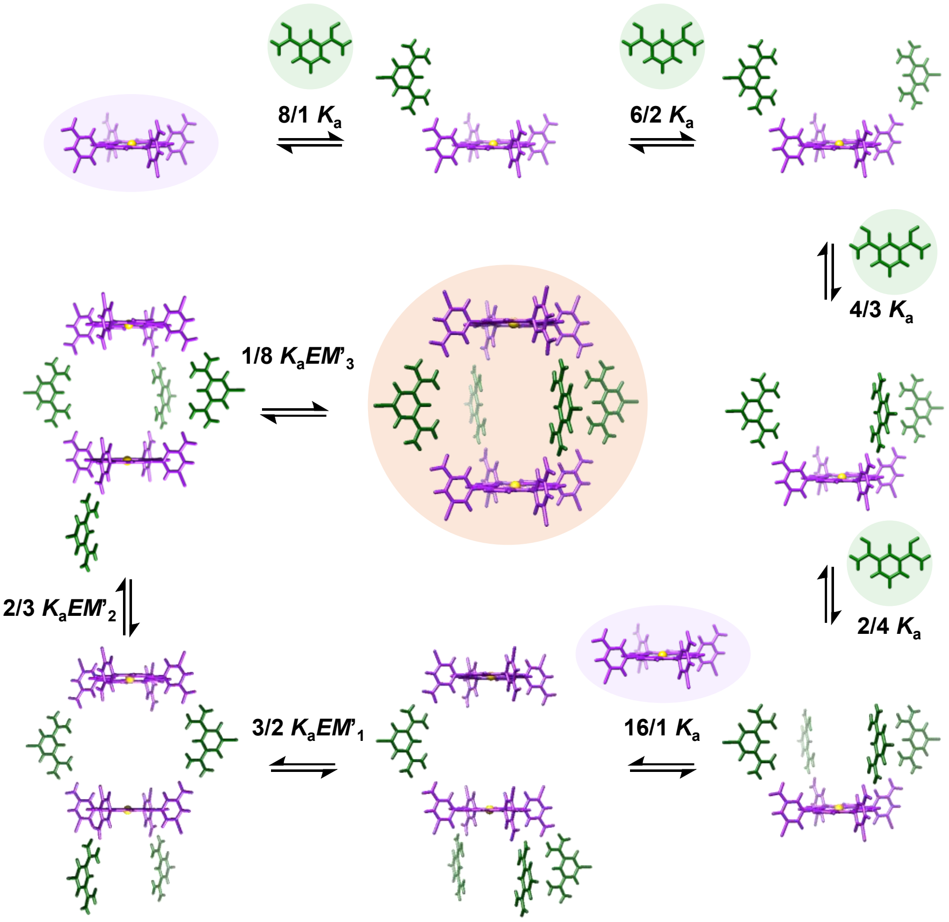

$$K_T = \frac{8 \cdot 6 \cdot 4 \cdot 2 \cdot 16 \cdot 3 \cdot 2 \cdot 1}{1 \cdot 2 \cdot 3 \cdot 4 \cdot 1 \cdot 2 \cdot 3 \cdot 8} K_a{}^8 \cdot EM'_1 \cdot EM'_2 \cdot EM'_3 = \mathbf{32 \cdot K_a{}^8 \cdot \overline{EM}'^3}$$

Figure S17. Schematic representation of the different equilibria involved in the stepwise formation of the $\mathbf{P_2 \cdot L_4}$ supramolecular assembly. The global association constant K_T was related to the reference binding constant between carboxylate and amidinium functions (K_a) and the derived statistical correction factor (32). Note that the effective molarity *EM* shown in the final equation is the average value of the 3 different *EMs* (EM'_1-EM'_3) present in these equilibria.

S8. Self-assembly in protic solvents and aqueous environments

As a logical step toward the formation of the $P_2{\cdot}L_4$ complex in an aqueous environment, the assembly of **P**^{O} and **L**^{O} in MeOH, a polar protic solvent, was tested. ^{1}H NMR titration experiments (analogous to those shown in ***Figure S1***) were performed in various mixtures of CD_3OD with either THF-D_8 (***Figure S18***) or DMSO-D_6, obtaining similar results. The molar ratio of the **P**^{O} and **L**^{O} components was varied from 1:0 (purple) to 0:1 (green). At a 1:2 **P**:**L** stoichiometry (orange), the spectra revealed a single, well-defined set of signals for the assembly that are different from those of the individual **P** and **L** components. However, due to deuterium exchange of the labile protons involved in the amidinium-carboxylate interaction and the inner pyrrolic positions, some signals are either not observed or significantly broadened, as seen for the β-pyrrolic signal (*D*). Besides, partial precipitation was observed when approaching the stoichiometric 1:2 ratio. Consequently, the use of polar protic solvents hinders the detailed characterization of the self-assembly process.

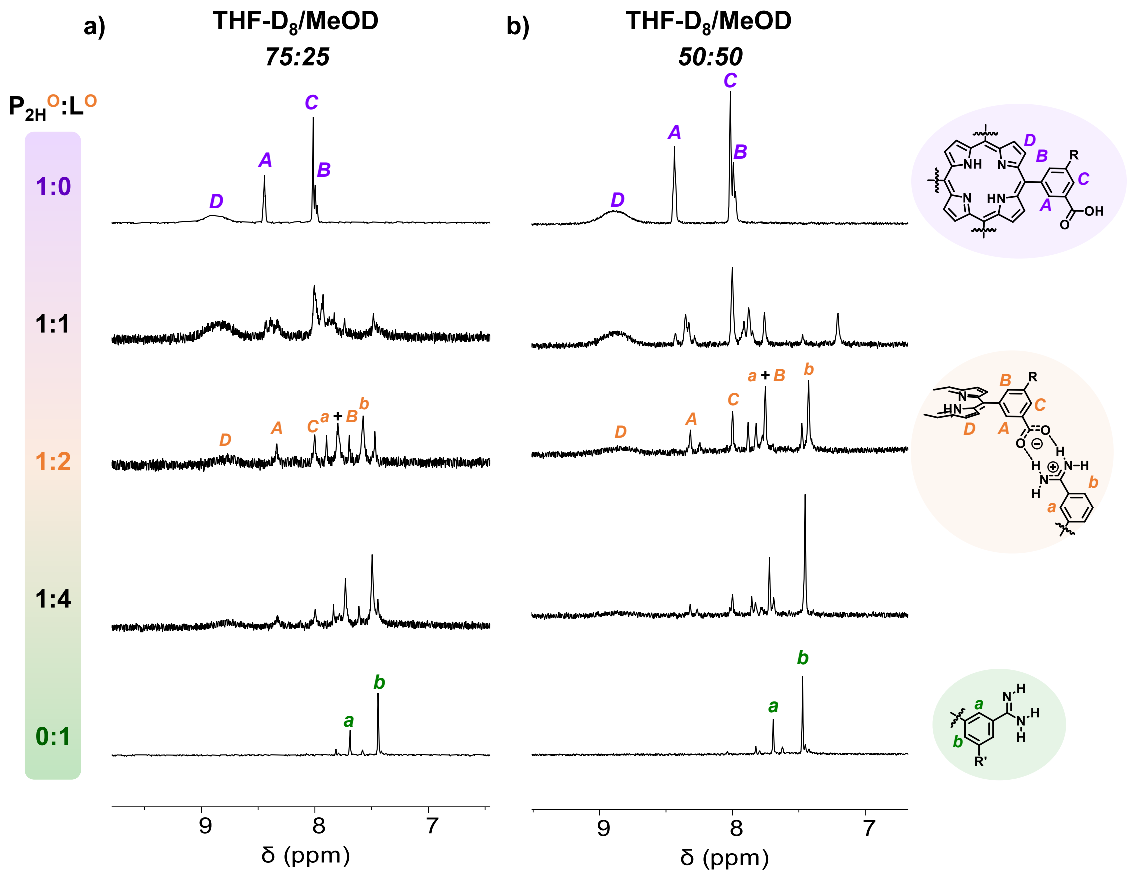

Figure S18. Aromatic regions of the ^{1}H NMR spectra in THF-D_8/MeOD mixture **a)** 75:25, and **b)** 50:50 obtained by varying the $\mathbf{P_{2H}}^{O}$:**L**^{O} ratio. Proportions were obtained from stock solutions of [$\mathbf{P_{2H}}^{O}$] = [**L**^{O}] = 0.6 mM.

A set of ^{1}H NMR experiments was then carried out to confirm the self-assembly of $\mathbf{P^{A}_{2}{\cdot}L^{A}_{4}}$ in aqueous environments, varying the proportion of $\mathbf{P^{A}}$ and $\mathbf{L^{A}}$ components from 1:0 (purple) to 0:1 (green), as shown in ***Figure S19***. Due to the broad signals observed in pure D_2O (see below), THF-D_8 was used as cosolvent. Starting from $\mathbf{P^{A}}$ (top spectrum) and going down in the set of spectra by increasing the relative concentration of $\mathbf{L^{A}}$, very weak, but measurable and reproducible changes were observed in the aromatic $\mathbf{P^{A}}$ protons, especially those labelled as **A**. Unfortunately, the presence of D_2O in the medium leads to the disappearance of exchangeable proton signals, such as those of the amidinium–carboxylate interactions or the NH protons of the porphyrin core, as well as to a broadening of the β-pyrrolic signals (*D*). By analyzing intermediate proportions, two distinct exchange regimes between bound (blue) and unbound units (purple for $\mathbf{P^{A}}$ and green for $\mathbf{L^{A}}$) were identified, although we must admit that not as clearly as for $\mathbf{P^{O}_{2}{\cdot}L^{O}_{4}}$ (see *Section S1*). For $\mathbf{P^{A}}$, a slow exchange process seems to be taking place at the NMR timescale (although only observable for the **A** signal). In contrast, $\mathbf{L^{A}}$ undergoes again fast exchange (*a* and *b*).

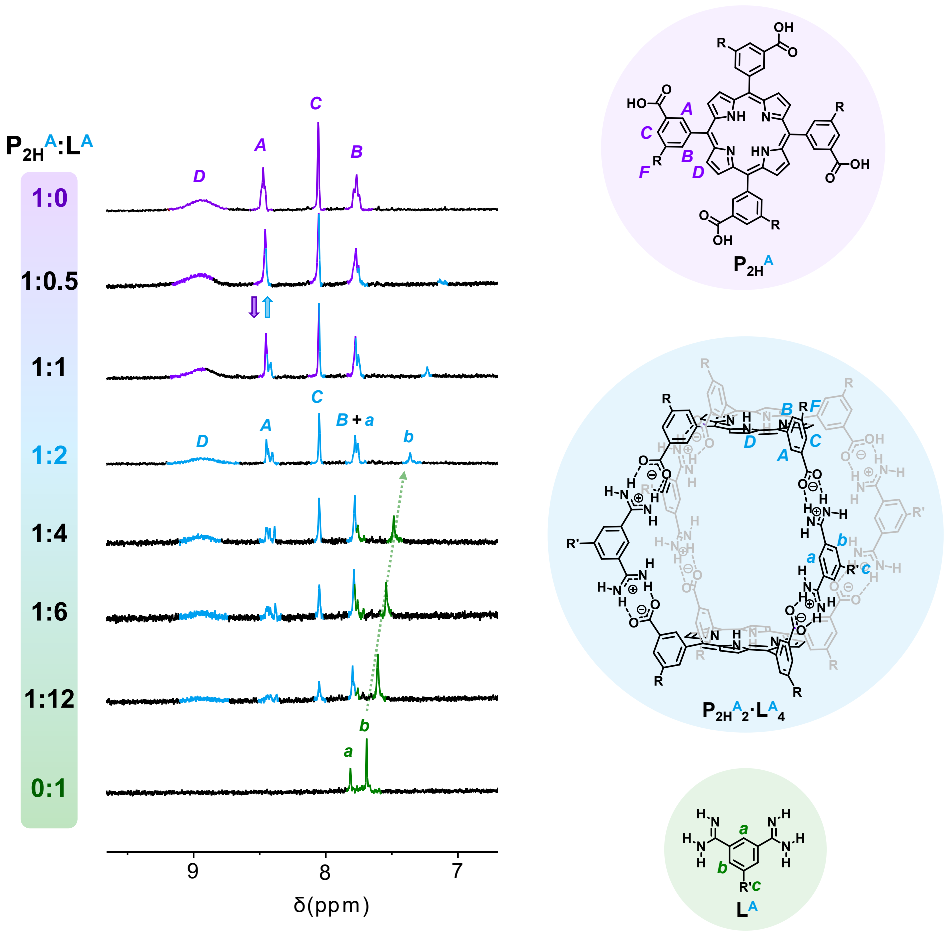

Figure S19. Selected regions of the ^{1}H NMR spectra in D_2O/THF-D_8 mixture (50:50) with 2 mM $NaHCO_3$, obtained by varying the $\mathbf{P_{2H}^{A}}$:$\mathbf{L^{A}}$ ratio. The signals marked in purple and green correspond to the free $\mathbf{P^{A}}$ and $\mathbf{L^{A}}$ subunits, respectively, while those in blue correspond to the self-assembled subunits ($\mathbf{P_{2H}^{A}{}_{2}{\cdot}L^{A}_{4}}$). Proportions were obtained from solutions of [$\mathbf{P_{2H}^{A}}$] = [$\mathbf{L^{A}}$] = 0.6 mM.

Finally, self-assembly of $\mathbf{P^A_2{\cdot}L^A_4}$ was further investigated in 100% D_2O using different buffers at varying concentrations (10 mM phosphate or 100 mM borate). As shown in ***Figure S20***, reproducible spectral changes were observed under all solvent conditions. The most pronounced chemical shift variations occur for the signals of $\mathbf{L^A}$ (*a* and *b*). However, the slow exchange observed previously for $\mathbf{P^A}$ was not detected in these buffered aqueous solutions, which provided rather broad NMR signals. DOSY NMR experiments in these buffered conditions were not helpful either in the analysis of the $\mathbf{P^A_2{\cdot}L^A_4}$ complex size.

Therefore, we conclude that, although the chemical shifts experienced by some protons can be regarded as evidence of intermolecular interactions, the quantitative formation of the $\mathbf{P^A_2{\cdot}L^A_4}$ complex in water could not be fully demonstrated.

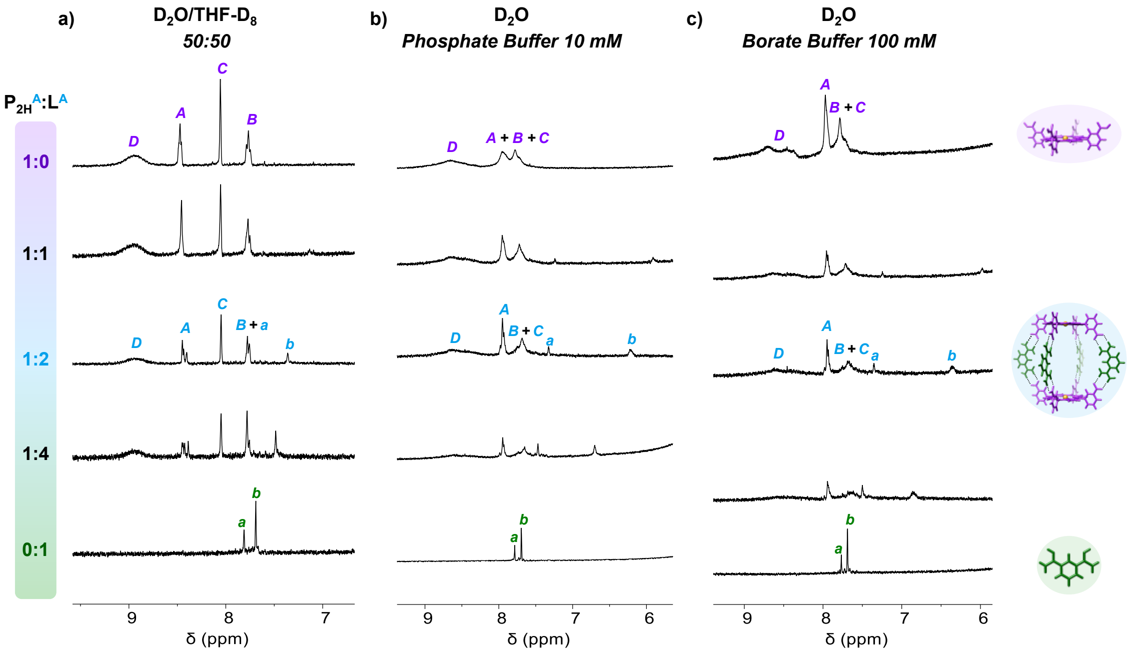

Figure S20. Aromatic regions of the ^1H NMR spectra in **a)** D_2O/THF-D_8 mixture (50:50) with 2 mM $NaHCO_3$, **b)** phosphate buffer 10 nM in D_2O, and **c)** borate buffer 100 mM in D_2O obtained by varying the $\mathbf{P_{2H}^A{:}L^A}$ ratio. Proportions were obtained from solutions of [$\mathbf{P_{2H}^A}$] = [$\mathbf{L^A}$] = 0.6 mM.

In order to confirm that the new **P^A^2·L^A^4** complex was assembled in THF/DMSO mixtures as the parent **P^O^2·L^O^4** complex and to verify that the polyethyleneglycol chains do not interfere with the self-assembly process, a set of experiments were executed in THF-D_8/DMSO-D_6 mixtures (50:50). As shown in ***Figure S21***, the molar ratio of the **P**^{A} and **L**^{A} components was varied from 1:0 (purple) to 0:1 (green). At a 1:2 **P**:**L** stoichiometry (blue), a single set of ^{1}H NMR resonances was detected, which differ from those of the individual precursors and integrate as expected for the **P**$^A_2$**·L**$^A_4$ supramolecular complex. Additionally, the previously observed dynamic behavior was maintained; specifically, the system exhibits slow exchange on the NMR timescale in the presence of an excess of **P**^{A}, whereas fast exchange is observed when the **L**^{A} is in excess.

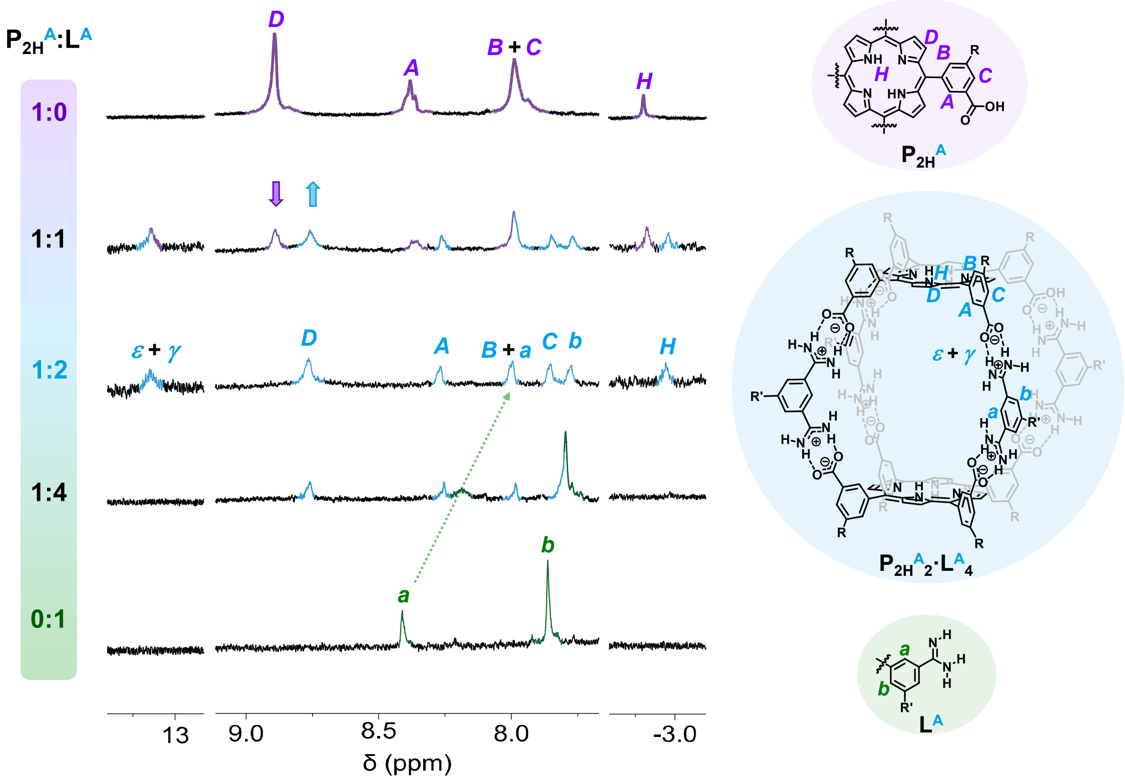

Figure S21. Selected regions of the ^{1}H NMR spectra in THF-D_8/DMSO-D_6 mixture (50:50) with 10 mM $NaHCO_3$, obtained by varying the **P**$_{2H}^A$:**L**^{A} ratio. Proportions were obtained from stock solutions of [**P**$_{2H}^A$] = [**L**^{A}] = 0.6 mM.

In order to study the stability of the **P**$^A_2$**·L**$^A_4$ complex in aqueous media, a titration was performed by adding increasing amounts of D_2O to a THF-D_8/DMSO-D_6 (50:50) solution of the assembly. As shown in ***Figure S22***, the complex begins to disassemble at approximately 10% D_2O content, characterized by slow exchange on the NMR timescale between the assembly and the free **P**^{A}. Upon reaching 20% D_2O, the complex is completely dissociated, with the resulting spectrum overlapping perfectly with those of the unbound components at the same conditions.

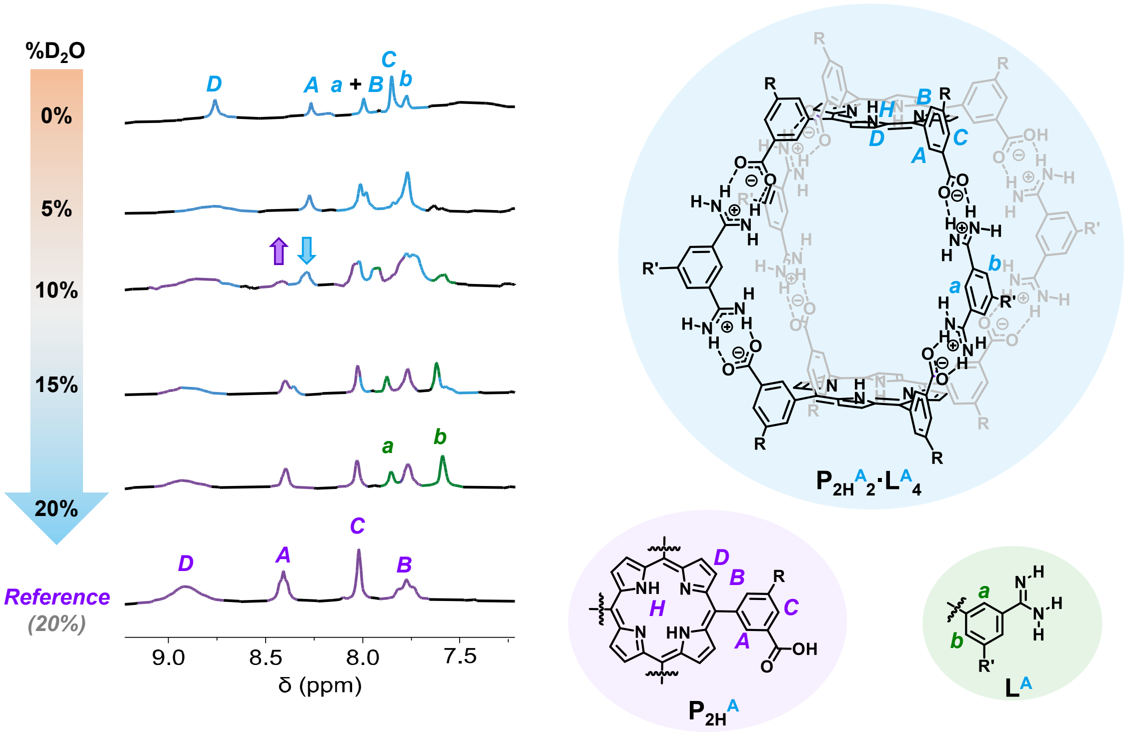

Figure S22. Selected regions of the ^{1}H NMR spectra of **$P_{2H}{}^{A}{}_{2}\cdot L^{A}{}_{4}$** (0.1mM) recorded in THF-D8/DMSO-D6 (50:50) mixture with increasing amounts of D_2O, ranging from 0% (top) to 20% (bottom) at 298 K. The ^{1}H NMR spectra of **$P_{2H}{}^{A}$** recorder under the same conditions (20% D_2O), is included as reference at the bottom of the stack.

S9. Host-Guest chemistry

To demonstrate the presence of an internal cavity within the $P_{Zn}{}^{O}{}_2\cdot L^{O}{}_4$ container with the ability to encapsulate guest molecules, a bidentate pyridine-based ligand, **di(pyridin-4-yl)buta-1,3-diyne** (**G**), was selected as a model guest. Based on computational models, the distance between the two Zn centers in the assembly was estimated to be approximately 1.7 nm. Given the typical Zn···N coordination distances and the linear geometry of **G** (approximately 1.2 nm in length),[10] this guest was considered a good fit for the cavity.

To determine the association constant of the host-guest complex, a titration experiment was performed by gradually adding increasing amounts of **G** to a fixed concentration of $P_{Zn}{}^{O}{}_2\cdot L^{O}{}_4$ in THF-D_8/DMSO-D_6 mixture (75:25), while monitoring the experiment by ^{1}H NMR spectroscopy (***Figure S23***).

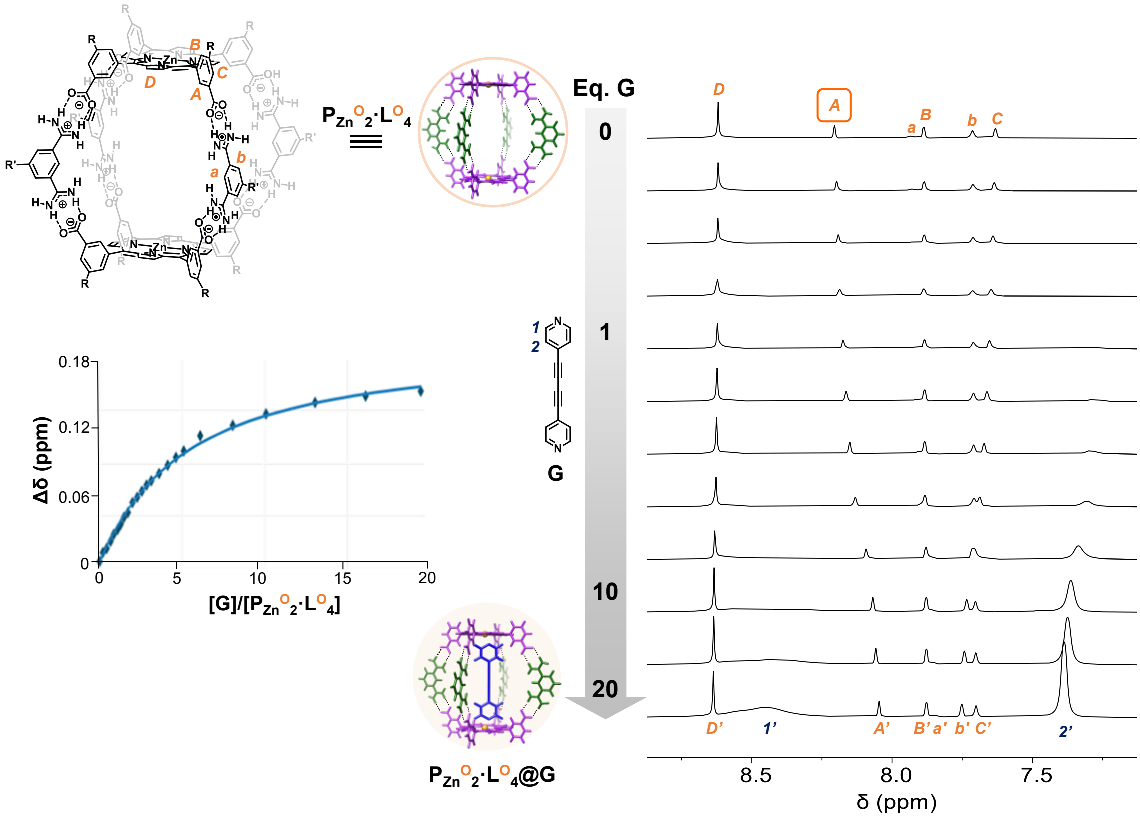

Figure S23. Titration experiment of $P_{Zn}{}^{O}{}_2\cdot L^{O}{}_4$ complex with increasing amounts of **G** monitored by ^{1}H NMR performed at a constant concentration of $1.2\cdot10^{-3}$ M in THF-D_8/DMSO-D_6 mixture (75:25) at 298 K. The chemical shift of the framed signals (*A*), as a function of **G** equivalents, was used to determine the association constants K_a by fitting the data to a 1:1 binding model.

A fast exchange at the NMR time scale was observed between free and bound species. The chemical shift changes of signal *A* were successfully fitted to a 1:1 binding isotherm, yielding an association constant K_a = 220 M^{-1}. Although this is a modest binding constant, it is attributed to competition by the solvent, as the coordinating nature of the solvent interferes with guest binding at the metal centers. In fact, under these same conditions, no measurable association constant

could be determined for the interaction between a reference TPP_{Zn} and pyridine. It is also important to mention that, under the same conditions, the monotopic $\mathbf{P_{Zn}}$ and di(pyridin-4-yl)buta-1,3-diyne, on one hand, or the shorter non-matching 4,4´-bipyridine guest and $\mathbf{P_2 \cdot L_4}$, on the other, did not show any evidence of association.

Supplementary References

1. Revvity Signals Software Inc. *ChemDraw 23.1.1*, ChemDraw Professional, Waltham, MA, USA, 2024.

2. Hypercube. *HyperChem Professional*, Hypercube Inc., Gainesville, FL, USA, 2007.

3. Pettersen, E. F.; Goddard, T. D.; Huang, C. C.; Couch, G. S.; Greenblatt, D. M.; Meng, E. C.; Ferrin, T. E. UCSF Chimera-A Visualization System for Exploratory Research and Analysis. *J. Comput. Chem*. **2004**, *25*, 1605-1612.

4. Ashton, P. R.; Anderson, D. W.; Brown, C. L.; Shipway, A. N.; Stoddart, J. F.; Tolley, M. S. The Synthesis and Characterization of a New Family of Polyamide Dendrimers. *Chem. Eur. J.* **1998**, *4*, 781-795.

5. Wolfe, A.L.; Duncan, K. K.; Lajiness, J. P.; Zhu, K.; Duerfeldt, A. S.; Boger, D. L. A Fundamental Relationship between Hydrophobic Properties and Biological Activity for the Duocarmycin Class of DNA-Alkylating Antitumor Drugs: Hydrophobic-Binding-Driven Bonding. *J. Med. Chem.* **2013**, *56*, 6845-6857.

6. Cauble, D.F.; Lynch, V.; Krische, M.J. Studies on the Enantioselective Catalysis of Photochemically Promoted Transformations: “Sensitizing Receptors” as Chiral Catalysts. *J. Org. Chem.* **2003**, *68*, 15-21.

7. Olive, A.G.L.; Parkan, K; Givelet, C.; Michl, J. Covalent Stabilization: A Sturdy Molecular Square from Reversible Metal-Ion-Directed Self-Assembly. *J. Am. Chem. Soc.* **2011**, *133*, 20108-20111.

8. Einstein, A. Eine neue Bestimmung der Moleküldimensionen. *Ann. Phys*. **1906**, *324*, 289-306.

9. Hogben, H. J.; Sprafke, J. K.; Hoffmann, M.; Pawlicki, M.; Anderson, H. L. Stepwise Effective Molarities in Porphyrin Oligomer Complexes: Preorganization Results in Exceptionally Strong Chelate Cooperativity. *J. Am. Chem. Soc.* **2011**, *133*, 20962-20969.

10. Ozores, H. L.; Amorín, M.; Granja, J. R. Self-Assembling Molecular Capsules Based on α,γ-Cyclic Peptides. *J. Am. Chem. Soc.* **2017**, *139*, 776-784.
